# AI models collapse when trained on recursively generated data

**DOI:** 10.1038/s41586-024-07566-y

**Published:** 2024-07-24

**Authors:** Ilia Shumailov, Zakhar Shumaylov, Yiren Zhao, Nicolas Papernot, Ross Anderson, Yarin Gal

**Affiliations:** 1 OATML, Department of Computer Science, University of Oxford, Oxford, UK; 2 Department of Applied Mathematics and Theoretical Physics, University of Cambridge, Cambridge, UK; 3 Department of Electrical and Electronic Engineering, Imperial College London, London, UK; 4 University of Toronto, Toronto, Ontario Canada; 5 Vector Institute, Toronto, Ontario Canada; 6 Department of Computer Science and Technology, University of Cambridge, Cambridge, UK; 7 School of Informatics, University of Edinburgh, Edinburgh, UK

**Keywords:** Computer science, Computational science

## Abstract

Stable diffusion revolutionized image creation from descriptive text. GPT-2 (ref. ^1^), GPT-3(.5) (ref. ^2^) and GPT-4 (ref. ^3^) demonstrated high performance across a variety of language tasks. ChatGPT introduced such language models to the public. It is now clear that generative artificial intelligence (AI) such as large language models (LLMs) is here to stay and will substantially change the ecosystem of online text and images. Here we consider what may happen to GPT-{*n*} once LLMs contribute much of the text found online. We find that indiscriminate use of model-generated content in training causes irreversible defects in the resulting models, in which tails of the original content distribution disappear. We refer to this effect as ‘model collapse’ and show that it can occur in LLMs as well as in variational autoencoders (VAEs) and Gaussian mixture models (GMMs). We build theoretical intuition behind the phenomenon and portray its ubiquity among all learned generative models. We demonstrate that it must be taken seriously if we are to sustain the benefits of training from large-scale data scraped from the web. Indeed, the value of data collected about genuine human interactions with systems will be increasingly valuable in the presence of LLM-generated content in data crawled from the Internet.

## Abstract (web summary)

Analysis shows that indiscriminately training generative artificial intelligence on real and generated content, usually done by scraping data from the Internet, can lead to a collapse in the ability of the models to generate diverse high-quality output.

## Main

The development of LLMs is very involved and requires large quantities of training data. Yet, although current LLMs^2,4–6^, including GPT-3, were trained on predominantly human-generated text, this may change. If the training data of most future models are also scraped from the web, then they will inevitably train on data produced by their predecessors. In this paper, we investigate what happens when text produced by, for example, a version of GPT forms most of the training dataset of following models. What happens to GPT generations GPT-{*n*} as *n* increases? We discover that indiscriminately learning from data produced by other models causes ‘model collapse’—a degenerative process whereby, over time, models forget the true underlying data distribution, even in the absence of a shift in the distribution over time. We give examples of model collapse for GMMs, VAEs and LLMs. We show that, over time, models start losing information about the true distribution, which first starts with tails disappearing, and learned behaviours converge over the generations to a point estimate with very small variance. Furthermore, we show that this process is inevitable, even for cases with almost ideal conditions for long-term learning, that is, no function estimation error. We also briefly mention two close concepts to model collapse from the existing literature: catastrophic forgetting arising in the framework of task-free continual learning^7^ and data poisoning^8,9^ maliciously leading to unintended behaviour. Neither is able to explain the phenomenon of model collapse fully, as the setting is fundamentally different, but they provide another perspective on the observed phenomenon and are discussed in more depth in the Supplementary Materials. Finally, we discuss the broader implications of model collapse. We note that access to the original data distribution is crucial: in learning tasks in which the tails of the underlying distribution matter, one needs access to real human-produced data. In other words, the use of LLMs at scale to publish content on the Internet will pollute the collection of data to train their successors: data about human interactions with LLMs will be increasingly valuable.

## What is model collapse?

### Definition 2.1 (model collapse)

**Fig. 1:**
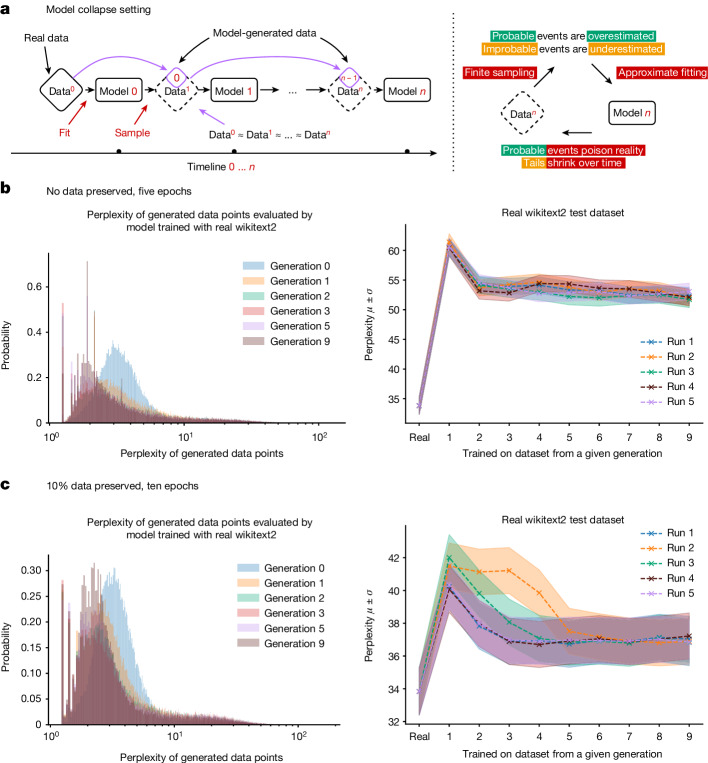
The high-level description of the feedback mechanism in the learning process. **a**, Model collapse refers to a degenerative learning process in which models start forgetting improbable events over time, as the model becomes poisoned with its own projection of reality. Here data are assumed to be human-curated and start off clean; then model 0 is trained and data are sampled from it; at step *n*, data are added to the overall data from step *n* − 1 and this combination is used to train model *n*. Data obtained with Monte Carlo sampling should ideally be statistically close to the original, provided that fitting and sampling procedures are perfect. This process depicts what happens in real life with the Internet: model-generated data become pervasive. **b**,**c**, Performance of OPT-125m models of different generations evaluated using the original wikitext2 test dataset. Shown on the left are the histograms of perplexities of each individual data training sequence produced by different generations as evaluated by the very first model trained with the real data. Over the generations, models tend to produce samples that the original model trained with real data is more likely to produce. At the same time, a much longer tail appears for later generations. Later generations start producing samples that would never be produced by the original model, that is, they start misperceiving reality based on errors introduced by their ancestors. The same plots are shown in 3D in the Supplementary Materials. On the right, average perplexity and its standard deviation are shown for each independent run. The *x* axis refers to the generation of the model. ‘Real’ refers to the ‘model 0’ trained on the original wikitext2 dataset; model 1 was trained on the data produced by model 0, model 2 was trained on data produced by model 1 and so on, with all generated datasets equal in size. We find that models trained on generated data are able to learn some of the original task, but with errors, as seen from the increase in perplexity.

Model collapse is a degenerative process affecting generations of learned generative models, in which the data they generate end up polluting the training set of the next generation. Being trained on polluted data, they then mis-perceive reality. The process is depicted in Fig. 1a. We separate two special cases: early model collapse and late model collapse. In early model collapse, the model begins losing information about the tails of the distribution; in late model collapse, the model converges to a distribution that carries little resemblance to the original one, often with substantially reduced variance.

This process occurs owing to three specific sources of error compounding over generations and causing deviation from the original model:

- **Statistical approximation error.** This is the primary type of error, which arises owing to the number of samples being finite, and disappears as the number of samples tends to infinity. This occurs because of a non-zero probability that information can get lost at every step of resampling.
- **Functional expressivity error.** This is a secondary type of error, arising owing to limited function approximator expressiveness. In particular, neural networks are only universal approximators as their size goes to infinity. As a result, a neural network can introduce non-zero likelihood outside the support of the original distribution or zero likelihood inside the support of the original distribution. A simple example of the expressivity error is if we tried fitting a mixture of two Gaussians with a single Gaussian. Even if we have perfect information about the data distribution (that is, infinite number of samples), model errors will be inevitable. However, in the absence of the other two types of error, this can only occur at the first generation.
- **Functional approximation error.** This is a secondary type of error, arising primarily from the limitations of learning procedures, for example, structural bias of stochastic gradient descent^10,11^ or choice of objective^12^. This error can be viewed as one arising in the limit of infinite data and perfect expressivity at each generation.

Each of the above can cause model collapse to get worse or better. More approximation power can even be a double-edged sword—better expressiveness may counteract statistical noise, resulting in a good approximation of the true distribution, but it can equally compound the noise. More often than not, we get a cascading effect, in which individual inaccuracies combine to cause the overall error to grow. For example, overfitting the density model causes the model to extrapolate incorrectly and assigns high-density regions to low-density regions not covered in the training set support; these will then be sampled with arbitrary frequency. It is worth noting that other types of error exist. For example, computers have limited precision in practice. We now turn to mathematical intuition to explain how the above give rise to the errors observed, how different sources can compound and how we can quantify the average model divergence.

## Theoretical intuition

Here we provide a theoretical intuition for the phenomenon of model collapse. We argue that the process of model collapse is universal among generative models that recursively train on data generated by previous generations. We quantify the sources of errors discussed in the previous section by examining two mathematical models, which prove to be simple enough to provide analytical expressions for quantities of interest, but also portray the phenomenon of model collapse: a discrete distribution in the absence of functional expressivity and approximation errors, and a multidimensional Gaussian approximation, portraying joint functional expressivity and statistical errors. We further illustrate the impact of all three jointly for a more complex setting of density estimation in Hilbert spaces in the Supplementary Materials.

The overall stochastic process we consider, which we call learning with generational data, is the following. The dataset at generation *i* is ${{\mathcal{D}}}_{i}$, comprising independent and identically distributed random variables ${X}_{j}^{i}$ with distribution *p*_*i*_, *j* ∈ {1,…, *M*_*i*_} denotes the size of the dataset. Going from generation *i* to generation *i* + 1, we aim to estimate the distribution of samples in ${{\mathcal{D}}}_{i}$, with an approximation ${p}_{{\theta }_{i+1}}$. This step is what we refer to as functional approximation, ${p}_{{\theta }_{i+1}}={{\mathcal{F}}}_{\theta }({p}_{i})$. The dataset ${{\mathcal{D}}}_{i+1}$ is then generated by sampling from ${p}_{i+1}={\alpha }_{i}{p}_{{\theta }_{i+1}}+{\beta }_{i}{p}_{i}+{\gamma }_{i}{p}_{0}$, with non-negative parameters *α*_*i*_, *β*_*i*_, *γ*_*i*_ summing to 1, that is, they represent proportions of data used from different generations. This corresponds to a mixing of data coming from the original distribution (*γ*_*i*_), data used by the previous generation (*β*_*i*_) and data generated by the new model (*α*_*i*_). We refer to this as the sampling step. For the mathematical models to come, we consider *β*_*i*_ = *γ*_*i*_ = 0, that is, data only from a single step are used, whereas numerical experiments are performed on more realistic choices of parameters.

### Discrete distributions with exact approximation

In this subsection, we consider a discrete probability distribution in absence of functional approximation and expressivity errors, that is, ${\mathcal{F}}(p)=p$. In this case, model collapse arises only because of statistical errors from the sampling step. At first, the tails (low-probability events) begin to disappear as a result of the low probability of sampling them and, over time, support of the distribution shrinks. Denoting the sample size as *M*, if we consider state *i* with probability $q\le \frac{1}{M}$, the expected number of samples with value *i* coming from those events will be less than 1. In practice, this would mean that we lose information about them. Considering more generally some state *i* with probability *q*, using standard conditional probability, we can show that the probability of losing information (that is, sampling no data at some generation) is equal to 1 − *q*, implying that the distribution must converge to a delta function positioned at some state, with the probability of ending up at a certain state equal to the probability of sampling said state from the original distribution.

This can be shown directly by considering the process $${{\bf{X}}}^{i}\to {\mathcal{F}}\,\to $$$${p}_{i+1}\to {{\bf{X}}}^{i+1}$$ as a Markov chain, as **X**^*i*+1^ only depends on **X**^*i*^. Furthermore, if all the $${X}_{j}^{i}$$ have the same value, then at the next generation, the approximated distribution will be exactly a delta function and therefore all of $${X}_{j}^{i+1}$$ will also have the same value. This implies that the Markov chain contains at least one absorbing state and therefore, with probability 1, it will converge to one of the absorbing states. This is a well-known fact, of which a proof is provided in the Supplementary Materials. For this chain, the only absorbing states are those corresponding to delta functions. As a result, as we follow the progress of model collapse, we are guaranteed to end up in a constant state, having lost all the information of the original distribution when the chain is absorbed. This argument also works in general owing to floating-point representations being discrete, making the Markov chain over the parameters of the model discrete. Thus, as long as the model parameterization allows for delta functions, we will get to it, because—owing to sampling errors—the only possible absorbing states are delta functions. On the basis of the discussion above, we see how both early model collapse, in which only the low-probability events get cut off, and late stage model collapse, in which the process begins to collapse into a single mode, must arise in the case of discrete distributions with perfect functional approximation.

### Multidimensional Gaussian

Following the discussion about discrete distributions, we now present a more generic result, which can be shown in the Gaussian approximation setting, in which each generation is approximated using the unbiased estimates of the mean and the variance. A similar result holds more generally, which we detail in the Supplementary Materials.

#### Theorem 3.1 (Gaussian model collapse)

Assume the original data are sampled from distribution ${{\mathcal{D}}}_{0}$ (not necessarily Gaussian), with non-zero sample variance. Assume *X*^*n*^ are fit recursively using the unbiased sample mean and variance estimators from the previous generation, ${X}_{j}^{n}| {\mu }_{n},{\Sigma }_{n} \sim {\mathcal{N}}({\mu }_{n},{\Sigma }_{n})$, with a fixed sample size. Then,

$${\mathbb{E}}[{{\mathbb{W}}}_{2}^{2}({\mathcal{N}}({\mu }_{n},{\Sigma }_{n}),{{\mathcal{D}}}_{0})]\to \infty ;\,{\Sigma }_{n}\,\mathop{\to }\limits^{{\rm{a}}.{\rm{s}}.}\,0\,\,{\rm{a}}{\rm{s}}\,\,n\to \infty ,$$

in which ${{\mathbb{W}}}_{2}$ denotes the Wasserstein-2 distance between the true distribution and its approximation at generation *n*.

In words, this implies that not only does the *n*th generation approximation diverge arbitrarily far from the original one but it also collapses to be zero variance as the number of generations increases, with probability 1. The results are very analogous to that seen in the discrete case, with this theorem illustrating the effect of late stage model collapse, in which the process begins to collapse to be zero variance. The early stage model collapse can also be seen and the interested reader is referred to the Supplementary Materials for a more in-depth discussion.

## Model collapse in language models

In this section, we evaluate the effect of model collapse on language models. We cover more interpretable machine learning models—VAEs and GMMs—in the Supplementary Materials. Code is publically available in ref. ^13^.

Model collapse is universal across various families of machine learning models. Yet, if small models such as GMMs and VAEs are normally trained from scratch, LLMs are different. They are so expensive to retrain from scratch that they are typically initialized with pre-trained models such as BERT^4^, RoBERTa^5^ or GPT-2 (ref. ^2^), which are trained on large text corpora. They are then fine-tuned to various downstream tasks^14^.

Here we explore what happens with language models when they are sequentially fine-tuned with data generated by other models. We can easily replicate all experiments covered in this paper with larger language models in non-fine-tuning settings to demonstrate model collapse. Given that training a single moderately large model produces twice the American lifetime’s worth of CO_2_ (ref. ^15^), we opted to not run such an experiment and instead focus on a more realistic setting for a proof of concept. Note that even the language experiments described in this paper took weeks to run. We evaluate the most common setting of training a language model—a fine-tuning setting for which each of the training cycles starts from a pre-trained model with recent data. The data here come from another fine-tuned pre-trained model. Because training is restricted to produce models that are close to the original pre-trained model, and data points generated by the models will generally produce very small gradients, the expectation here may be that the model should only change moderately after fine-tuning. We fine-tune the OPT-125m causal language model made available by Meta through Hugging Face^6^.

We fine-tune it on the wikitext2 dataset^16^. For data generation from the trained models, we use a five-way beam search. We block training sequences to be 64 tokens long; then, for each token sequence in the training set, we ask the model to predict the next 64 tokens. We go through all of the original training dataset and produce an artificial dataset of the same size. Because we go through all of the original dataset and predict all of the blocks, if the model had 0 error, it would produce the original wikitext2 dataset. Training for each generation starts with generation from the original training data. Each experiment is run five times and the results are shown as five separate runs with different randomness seeds. The original model fine-tuned with real wikitext2 data obtains 34 mean perplexity, from the zero-shot baseline of 115, that is, it successfully learns the task. Finally, to be as realistic as possible, we use the best-performing model on the original task, evaluated using the original wikitext2 validation set, as the base model for the subsequent generations, meaning that—in practice—observed model collapse can be even more pronounced. Here we consider two different settings:

- Five epochs, no original training data. Here the model is trained for five epochs starting on the original dataset but with no original data retained for subsequent runs. The overall original task performance is presented in Fig. 1b. We find that training with generated data allows us to adapt to the underlying task, losing some performance, from 20 to 28 perplexity points.
- Ten epochs, 10% of original training data preserved. Here the model is trained for ten epochs on the original dataset and with every new generation of training, a random 10% of the original data points is sampled. The overall original task performance is presented in Fig. 1c. We find that preservation of the original data allows for better model fine-tuning and leads to only minor degradation of performance.

Both training regimes lead to degraded performance in our models, yet we do find that learning with generated data is possible and models can successfully learn (some of) the underlying task. In particular, from Fig. 1 and their 3D versions in the Supplementary Materials, we see that model collapse occurs, as the density of samples with low perplexity begins to accumulate over the generations. This in turn makes it likely that, over the generations, the sampled data will similarly collapse to a delta function.

It is important to note here that the observed behaviour is in line with the general intuition established in the section ‘Theoretical intuition’. To be precise, in all experiments, generational learning is only performed on a finite (usually small) number of generations, whereas claims of the section ‘Theoretical intuition’ are mostly presented in the limit of generations going to infinity. However, as seen from experiments on VAEs and GMMs in the Supplementary Materials, convergence to delta functions and specific rates of such convergence are highly related to the specifics of the problem considered, and complete collapse may or may not occur, even after a small number of steps. This is further illustrated theoretically in the Supplementary Materials, in which potentially notable divergence from the original model can occur even after a few generations.

Figure 1b,c on the left shows histograms of individual data-point perplexities generated by the models of different generations as is evaluated by the first model developed with real wikitext2 training data. Here, over the generations, models tend to produce more sequences that the original model would produce with the higher likelihood. The observed effect is similar to that described for VAEs and GMMs in the Supplementary Materials, in which—over the generations—models started to produce samples that would be produced with higher probabilities by the original model. At the same time, we discover that generated data have much longer tails, suggesting that some of the data would never be produced by the original model—these are the errors that accumulate because of the learning with generational data.

### Example 1

Example of text outputs of an OPT-125m model affected by model collapse—models degrade over generations, for which each new generation is trained on data produced by the previous generation.

- **Input:** some started before 1360 — was typically accomplished by a master mason and a small team of itinerant masons, supplemented by local parish labourers, according to Poyntz Wright. But other authors reject this model, suggesting instead that leading architects designed the parish church towers based on early examples of Perpendicular.
- **Outputs:**
- **Gen 0:** Revival architecture such as St. John’s Cathedral in London. The earliest surviving example of Perpendicular Revival architecture is found in the 18th @-@ century Church of Our Lady of Guernsey, which dates from the late 19th century. There are two types of perpendicular churches : those.
- **Gen 1:** architecture such as St. Peter’s Basilica in Rome or St. Peter’s Basilica in Buenos Aires. There is no evidence that any of these buildings were built during the reign of Pope Innocent III, but it is possible that they may have been built during the reign of his successor, Pope Innocent.
- **Gen 5:** ism, which had been translated into more than 100 languages including English, French, German, Italian, Spanish, Portuguese, Dutch, Swedish, Norwegian, Polish, Hungarian, Slovak, Lithuanian, Estonian, Finnish, Romanian, Bulgarian, Turkish, Croatian, Serbian, Ukrainian, Russian, Kazakh, Kyrgyz.
- **Gen 9:** architecture. In addition to being home to some of the world’s largest populations of black @-@ tailed jackrabbits, white @-@ tailed jackrabbits, blue @-@ tailed jackrabbits, red @-@ tailed jackrabbits, yellow @-.

### Ablation: Repetitions

We find that data generated by language models in our experiments end up containing a large number of repeating phrases, as in Example 1. The repeating problem has been observed in nearly all text-generation models^17,18^ and, to rule this out as the cause of model collapse, we further provide numerical experiments when models are explicitly encouraged to produce non-repeating sequences with a repeating penalty of 2.0. We find that this causes the models to produce lower score continuations to avoid using repeats, which—as a result—causes the consequent models to perform even worse. Model perplexities shift across the generations towards more probable token sequences, as measured using the model trained on the original real data distribution. Further illustrations are provided in the Supplementary Materials. In particular, enforcing this for the LLM experiments causes the perplexity to double compared with the original. Models remain as susceptible to model collapse, if not more.

The described process demonstrates that fine-tuning of language models does not curb the effects of model collapse and models that are being fine-tuned are also vulnerable. We find that, over the generations, models tend to produce more probable sequences from the original data and start introducing their own improbable sequences, that is, errors.

## Discussion

We now discuss the implications of model collapse on the underlying learning dynamics of LLMs. Long-term poisoning attacks on language models are not new. For example, we saw the creation of click, content and troll farms, a form of human ‘language models’, whose job is to misguide social networks and search algorithms. The negative effect that these poisoning attacks had on search results led to changes in search algorithms. For example, Google downgraded farmed articles^19^, putting more emphasis on content produced by trustworthy sources, such as education domains, whereas DuckDuckGo removed them altogether^20^. What is different with the arrival of LLMs is the scale at which such poisoning can happen once it is automated. Preserving the ability of LLMs to model low-probability events is essential to the fairness of their predictions: such events are often relevant to marginalized groups. Low-probability events are also vital to understand complex systems^21^.

Our evaluation suggests a ‘first mover advantage’ when it comes to training models such as LLMs. In our work, we demonstrate that training on samples from another generative model can induce a distribution shift, which—over time—causes model collapse. This in turn causes the model to mis-perceive the underlying learning task. To sustain learning over a long period of time, we need to make sure that access to the original data source is preserved and that further data not generated by LLMs remain available over time. The need to distinguish data generated by LLMs from other data raises questions about the provenance of content that is crawled from the Internet: it is unclear how content generated by LLMs can be tracked at scale. One option is community-wide coordination to ensure that different parties involved in LLM creation and deployment share the information needed to resolve questions of provenance. Otherwise, it may become increasingly difficult to train newer versions of LLMs without access to data that were crawled from the Internet before the mass adoption of the technology or direct access to data generated by humans at scale.

## Online content

Any methods, additional references, Nature Portfolio reporting summaries, source data, extended data, supplementary information, acknowledgements, peer review information; details of author contributions and competing interests; and statements of data and code availability are available at 10.1038/s41586-024-07566-y.

## Supplementary information

Supplementary Information

Supplementary Data

## Data Availability

Data generation code for GMM experiments is available in ref. ^13^. Data used for VAE experiments are available in ref. ^22^. Data used for LLM experiments are available in ref. ^16^.

## Code availability

Code for all experiments is publically available in ref. ^13^.
