# Author Correction: AI models collapse when trained on recursively generated data

**DOI:** 10.1038/s41586-025-08905-3

**Published:** 2025-03-21

**Authors:** Ilia Shumailov, Zakhar Shumaylov, Yiren Zhao, Nicolas Papernot, Ross Anderson, Yarin Gal

**Affiliations:** 1 OATML, Department of Computer Science, University of Oxford, Oxford, UK; 2 Department of Applied Mathematics and Theoretical Physics, University of Cambridge, Cambridge, UK; 3 Department of Electrical and Electronic Engineering, Imperial College London, London, UK; 4 University of Toronto, Toronto, Ontario Canada; 5 Vector Institute, Toronto, Ontario Canada; 6 Department of Computer Science and Technology, University of Cambridge, Cambridge, UK; 7 School of Informatics, University of Edinburgh, Edinburgh, UK

**Keywords:** Computer science, Computational science

Correction to: *Nature* 10.1038/s41586-024-07566-y Published online 24 July 2024

In the version of the article initially published, in the “Theoretical intuition” section, the text “For the mathematical models to come, we consider *αi* = *γi* = 0” should have read “For the mathematical models to come, we consider “*βi* = *γi* = 0,” as is now amended in the HTML and PDF versions of the article.

