# Supplementary material for: AI models collapse when trained on recursively generated data
Source: Nature. 2024 Jul 24;631(8022):755–9. doi: 10.1038/s41586-024-07566-y (PMC11269175; doi:10.1038/s41586-024-07566-y)
Supplement: Supplementary file 1 — Supplementary Information [file 41586_2024_7566_MOESM1_ESM.pdf]

nature portfolio

https://doi.org/10.1038/s41586-024-07566-y

Supplementary information

AI models collapse when trained on recursively generated data

In the format provided by the authors and unedited

AI Models Collapse When Trained on Recursively Generated Data: Supplementary Material

1 Related work

In this section we are going to cover two closest concepts to *model collapse* from existing literature: catastrophic forgetting and data poisoning. Neither is able to explain the phenomenon of *model collapse* fully, as the setting is fundamentally different, but they provide another perspective on the observed phenomenon.

1.1 Continual learning and catastrophic forgetting

Unlike traditional machine learning which seeks to learn from a static data distribution, *continual learning* attempts to learn from a dynamic one, where data are supplied in a sequential fashion [1]. This tends to be task-based, where data are provided with delineated task boundaries; *e.g.*, classifying dogs from cats and recognising handwritten digits. Our work is more similar to task-free continual learning [2] where data distributions gradually change without the notion of separate tasks. Our work examines a particular scenario in which the changed data distributions arise from the model itself, as a result of training in the previous iteration. A typical challenge in continual learning is that the model forgets previous samples when learning new information; this is known as *catastrophic forgetting* [3]. A typical way of preventing it is to use regularisations (*e.g.* Memory Aware Synpass [4]) or just rely on data (*e.g.* Learning without Forgetting [5]). This has an indirect connection to our work, yet differs since the data in the process of *model collapse* are generated by different generations of models.

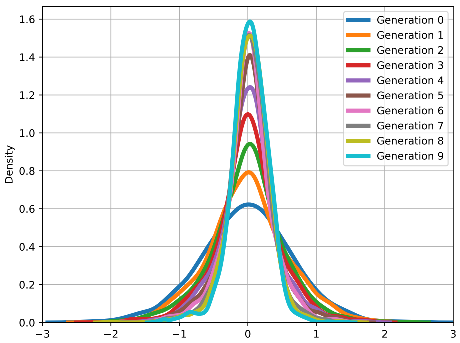

Fig. 1: Changing distribution of latents over the learning process with generated data as perceived by the original encoder. Just as with the Gaussian case described above, the tails get washed away and the model arrives at the mean representation of the underlying data.

1.2 Data poisoning

Poisoning attacks are crafted and inserted during training in order to degrade the model's performance when deployed [6]. Malicious data can be inserted into training data to induce unintended behaviors that can be activated by special triggers [7]. The early literature on data poisoning focused mainly on supervised learning, where classifiers are trained with labeled samples. But with the emergence of contrastive learning [8] and LLMs [9], more recent models are trained with large-scale web crawls, making data poisoning attacks more feasible on these untrustworthy web sources. Recent studies have demonstrated that web-scale datasets can be poisoned by introducing malicious data into a small percentage of samples [10, 11].

Example of text outputs of an OPT-125m model affected by *Model collapse* – models degrade over generations, where each new generation is trained on data produced by the previous generation.

Input: some started before 1360 — was typically accomplished by a master mason and a small team of itinerant masons, supplemented by local parish labourers, according to Poyntz Wright. But other authors reject this model, suggesting instead that leading architects designed the parish church towers based on early examples of Perpendicular

Outputs:

Gen 0: Revival architecture such as St. John's Cathedral in London. The earliest surviving example of Perpendicular Revival architecture is found in the 18th @-@ century Church of Our Lady of Guernsey, which dates from the late 19th century. There are two types of perpendicular churches : those

Gen 1: architecture such as St. Peter's Basilica in Rome or St. Peter's Basilica in Buenos Aires. There is no evidence that any of these buildings were built during the reign of Pope Innocent III, but it is possible that they may have been built during the reign of his successor, Pope Innocent

Gen 3: architecture such as St. Peter's Basilica (now part of the Church of Jesus Christ of Latter @-@ day Saints) or St. Peter's Basilica (now part of the Church of Jesus Christ of Latter @-@ day Saints). There is no evidence that any of these buildings

Gen 5: ism, which had been translated into more than 100 languages including English, French, German, Italian, Spanish, Portuguese, Dutch, Swedish, Norwegian, Polish, Hungarian, Slovak, Lithuanian, Estonian, Finnish, Romanian, Bulgarian, Turkish, Croatian, Serbian, Ukrainian, Russian, Kazakh, Kyrgyz

Gen 7: architecture in England. In an interview with The New York Times, Wright said : " I don 't think there is anything wrong with me being able to do what I want to do. It just doesn 't work for me. " He added : " I don 't know if you can call it funny,

Gen 9: architecture. In addition to being home to some of the world's largest populations of black @-@ tailed jackrabbits, white @-@ tailed jackrabbits, blue @-@ tailed jackrabbits, red @-@ tailed jackrabbits, yellow @-

2 Regularized Density Estimation in Hilbert Space

Here we consider a model of density estimation in a reproducing kernel Hilbert space (RKHS) presented by Kazashi and Nobile [12]. This choice is motivated by the similarity of *model collapse* to self-distillation; what is more, in this setting a closed form solution can be derived. To be precise, Mobahi et al. [13] analyzed the effect of multi-stage regularized regression in a RKHS – we show that similarly, in expectation, the density estimator collapses on to the topmost directions in the data.

Let $(\mathcal{X}, \mathcal{B}, \mu)$ be a measure space, and let $(\mathcal{N}_K, \langle \cdot, \cdot \rangle_K, \| \cdot \|_K)$ denote the RKHS associated with the positive definite kernel $K : \mathcal{X} \times \mathcal{X} \to \mathbb{R}$. The full assumptions and the corresponding derivations are provided in Section 3.2.1. We further define the integral operator $T_K : L^2_\mu(\mathcal{X}) \to L^2_\mu(\mathcal{X})$ by $T_K g := \int_{\mathcal{X}} K(\cdot, x) g(x) \mathrm{d}\mu(x)$, for $g \in L^2_\mu(\mathcal{X})$. Let $Y_1, \ldots, Y_M : \Omega \to \mathcal{X}$ be independent random variables that follow the distribution defined by a density $y \in \mathcal{N}_K$ with respect to μ. Similar to Kazashi and Nobile [12], we are approximating y of the form $y(\cdot) \approx \sum_{n=1}^N a_n K(x_n, \cdot)$ for $X = \{x_1, \ldots, x_N\} \subset \mathcal{X}$ a set of specifically chosen points, defining the approximation space $V_N := \operatorname{span}\{K(x_j, \cdot) \mid j = 1, \ldots, N\}$. The idealised regularised density estimation would consist of solving the following constrained optimistaion problem:

$$\arg\min_{u \in V_N} \quad \frac{1}{2}\|u\|_K^2 \qquad \text{s.t.} \quad \frac{1}{2}\|u - y\|^2_{L^2_\mu(\mathcal{X})} \leq \frac{\epsilon_n}{2}.$$

However, in the density estimation setting we do not have access to the true density, but just samples. Therefore the problem we consider is a sample approximation of the ideal problem:

$$f_n^* = \arg\min_{u \in V_N} \quad \frac{1}{2}\|u\|_K^2$$

$$\text{s.t.} \ \frac{1}{2}\|u\|^2_{L^2_\mu(\mathcal{X})} - \frac{1}{M}\sum_{m=1}^{M} u\left(Y_m^n\right) + \frac{1}{2}\left[\hat{A}^{-1/2} k_{Y^n}\right]^\top \left[\hat{A}^{-1/2} k_{Y^n}\right] \leq \frac{\epsilon_n}{2},$$

where we define $\hat{A}_{jk} \equiv \langle K(x_j, \cdot), K(x_k, \cdot)\rangle_{L^2_\mu(\mathcal{X})}$, $A_{jk} \equiv K(x_j, x_k)$, and we let positive definite $P \equiv \hat{A}^{-1/2} A \hat{A}^{-1/2}$, and $k(\cdot)_j \equiv K(x_j, \cdot)$, and similarly $k_{(Y)_j} \equiv \frac{1}{M}\sum_{m=1}^M K(x_j, Y_m)$, for $j = 1, \ldots, N$. As P is symmetric positive definite, we can write it as $P = V^\top D V$, with D diagonal. Let $d_{\min}$ and $d_{\max}$ denote its smallest and largest eigenvalues, and let $B_n = \prod_{i=0}^n (c_i D + I)^{-1}$. Now, considering the process of learning with generational data, we have $Y^{n+1} \sim f_n^*$ and $f_0^* \equiv y$, which lets us derive the following:

Theorem 2.1. Starting with $v_0 = V\hat{A}^{-1/2}[T_K y](x_{1:N})$, such that $\|v_0\| > \epsilon$ and $M \to \infty$ we have that the process does not collapse for at least $\underline{n}$ generations,

$$\underline{n} = \frac{d_{\min}}{d_{\max}}\left(\frac{\|v_0\|}{\sqrt{\epsilon}} - 1\right).$$

In this case, for $n \leqslant \underline{n}$, the average density estimate can be written exactly as

$$\mathbb{E}[f_n^*] = \left[V\hat{A}^{-1/2}k(\cdot)\right]^\top B_n \left[V\hat{A}^{-1/2}[T_K y](x_{1:N})\right]. \tag{1}$$

In particular, for d_j and d_k pair of diagonal elements of D with $d_k < d_j$, the following inequality holds:

$$\frac{B_{n-1}[k,k]}{B_{n-1}[j,j]} \geq \left(\frac{\frac{\|v_0\|}{\sqrt{\epsilon}} - 1 + \frac{d_j}{d_{\max}}}{\frac{\|v_0\|}{\sqrt{\epsilon}} - 1 + \frac{d_k}{d_{\max}}}\right)^n.$$

This, similarly to Mobahi et al. [13], tells us that the process of *model collapse* in this case progressively limits the number of basis functions to represent the density in the average case scenario, considered in the limit of infinite samples. This model, for the sake of simplicity, has been stated with $M \to \infty$. As such, Theorem 2.1 portrays the process of *model collapse* occuring due to the combination of functional expressivity and functional approximation errors. The model similarly allows for analysis in the case of finite M, but that is left for future work.

3 Theoretical Intuition: Expanded

In this section we provide a more in-depth theoretical intuition for the phenomenon of *model collapse*. We argue that the process of *model collapse* is universal among generative models that recursively train on data generated by previous generations. We illustrate this using simple mathematical models that allow for analytical expressions of quantities of interest, while also portraying the phenomenon of *model collapse*. In the main section we examined three models: a discrete distribution in the absence of functional expressivity and approximation errors; a multi dimensional Gaussian approximation, portraying joint functional expressivity and statistical errors; and density estimation in Hilbert Spaces, illustrating all three errors jointly. Here, we discuss in more depth the model of a continuous single dimensional Gaussian, a noisy multidimensional model, and density estimation in a reproducing kernel Hilbert space.

3.1 Continuous random variables

3.1.1 1D Gaussian

In this subsection, we consider learning data coming from a single dimensional Gaussian $X^0 \sim \mathcal{N}(\mu, \sigma^2)$. We estimate the density using the sample mean and variance and fitting a single dimensional Gaussian:

$$\mu_{i+1} = \frac{1}{M_i}\sum_j X_j^i; \quad \sigma_{i+1}^2 = \frac{1}{M_i - 1}\sum_j (X_j^i - \mu_{i+1})^2. \tag{2}$$

Note here, that if we were to use maximum likelihood estimation, we would instead arrive at a biased variance estimator. With these estimates, the functional approximation step simply corresponds to considering a normal distribution with these

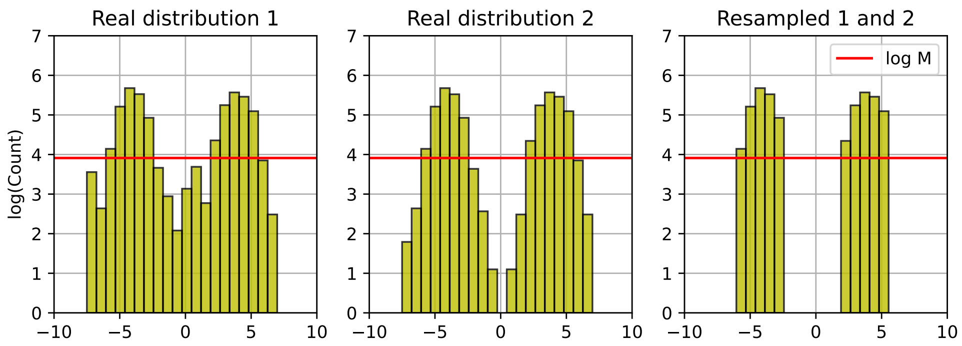

Fig. 2: Shown in the middle is a histogram plot of samples from a Gaussian mixture with means $(-4, 4)$ and variances of 1. To the left of it is a similar distribution, but with 'fatter' tails, and on the right the same histograms are shown, but with low probability events being cut off due to finite resampling. Although distributions 1 and 2 are very different, when resampled (only assuming the expected behaviour), the tails get cut off, leading to the same observed distribution. In this case this is all states with probability less than $1/M$, or equivalently, bins with $\log \texttt{Count} \leq \log M$.

parameters, which we can sample from:

$$X_j^{i+1} | \mu_{i+1},\ \sigma_{i+1} \sim \mathcal{N}(\mu_{i+1}, \sigma_{i+1}^2). \tag{3}$$

This provides us with the conditional distribution of X_j^i, which allows us to calculate the full distribution of X_j^i. From Equation (4), we see that even after the first approximation, the distribution of X_j^i is no longer normal, it follows a variance-gamma distribution [14]. However, instead of writing the probability density function at each generation, we can explicitly construct them in terms of independent random variables. In particular, it is well known [15] that μ_1 and σ_1 are independent, with $\mu_1 \sim \mathcal{N}(\mu, \frac{\sigma^2}{M_0})$ and $(M_0 - 1)\sigma_1^2 \sim \sigma^2 \Gamma(\frac{M_0 - 1}{2}, \frac{1}{2})$. In what follows we will denote with Z random variables that are distributed with $\mathcal{N}(0, 1)$ and with S^i random variables that are distributed with $\frac{1}{M_{i-1}-1}\Gamma(\frac{M_{i-1}-1}{2}, \frac{1}{2})$.

$$X_j^0 = \mu + \sigma Z_j^0; \quad X_j^1 = \mu + \frac{\sigma}{\sqrt{M_0}} Z^1 + \sigma\sqrt{S^1} Z_j^1; \quad \ldots \tag{4}$$

$$\begin{aligned} X_j^n = \mu &+ \frac{\sigma}{\sqrt{M_0}} Z^1 + \frac{\sigma}{\sqrt{M_1}}\sqrt{S^1} Z^2 + \ldots \\ &+ \frac{\sigma}{\sqrt{M_{n-1}}}\sqrt{S^1 \times \cdots \times S^{n-1}} Z^n + \sigma\sqrt{S^1 \times \cdots \times S^n} Z_j^n. \end{aligned} \tag{5}$$

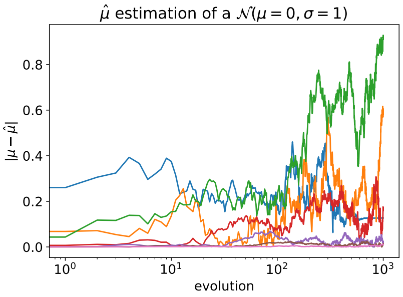

(a) Mean estimation

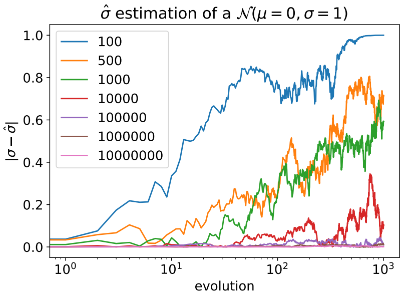

(b) Standard Deviation

Fig. 3: Recursive fitting-sampling of a 1D Gaussian with different numbers of samples drawn. We find that unless sampled a very large number of times, *i.e.* <100000, both standard deviation and mean get significantly affected. Here we report a single run; while re-running the experiment changes the initial performance, both μ and σ drift over time. The overall graph looks quite similar to that of a Gaussian random walk.

These are not joint distributions, as Z^n and S^n depend directly on Z_j^{n-1}, but when considering X_j^n on its own the formula above provides all the information about the full distribution.

The first thing we may try calculating is the variance. It is possible to find its exact value, but the mean and variance of the square root of gamma distribution are expressed in terms of gamma functions, making the result quite clunky. In what follows, we will expand everything to second order in each of $(1/M_i)$ as we assume each sample size to be large (in practice this becomes quite accurate after $M \sim 100$). We then find that

$$\frac{1}{\sigma^2}\operatorname{Var}(X_j^n) = \frac{1}{M_0} + \frac{1}{M_1} + \cdots + \frac{1}{M_{n-1}} + 1 + \mathcal{O}(2).$$

And if we were to assume that $M_i = M$ are constant, we would find that:

$$\operatorname{Var}(X_j^n) = \sigma^2\left(1 + \frac{n}{M}\right); \quad \mathbb{E}(X_j^n) = \mu.$$

This means that as $n \to \infty$, the variance diverges linearly. This is the same scaling as for a single dimensional Gaussian random walk. We can further see the similarities in numerical experiments shown on Figure 3 for a range of different sample sizes, confirming these theoretical intuitions.

Even though the variance of X_j^n diverges, it does not provide us with any information of what the corresponding estimates of μ_{n+1} and σ_{n+1}^2 are, or how far they are from the original μ and σ. In particular, we may want to consider what the distance would be between the true distribution and the approximated distribution at step $n+1$. To measure this we can consider the Wasserstein-2 distance between two

normals:

$$R_{W_2}^{n+1} := \mathbb{W}_2^2\left(\mathcal{N}(\mu,\sigma^2), \mathcal{N}(\mu_{n+1},\sigma_{n+1}^2)\right) = \|\mu_{n+1}-\mu\|^2 + \|\sigma_{n+1}-\sigma\|^2$$

Now we can calculate the risk that occurs due to finite sampling, *i.e.* what the expected value of the distance is (expanding in $1/M_i$):

$$\mathbb{E}_{\mu_{n+1},\sigma_{n+1}^2}\left[R_{W_2}^{n+1}\right] = \frac{3}{2}\sigma^2\left(\frac{1}{M_0}+\frac{1}{M_1}+\dots+\frac{1}{M_n}\right)+\mathcal{O}(2), \tag{6}$$

$$\mathrm{Var}_{\mu_{n+1},\sigma_{n+1}^2}\left[R_{W_2}^{n+1}\right] = \frac{1}{2}\sigma^4\left(\frac{3}{M_0^2}+\frac{3}{M_1^2}+\dots+\frac{3}{M_n^2}+\sum_{i\neq j}\frac{4}{M_iM_j}\right)+\mathcal{O}(3). \tag{7}$$

This result allows us to interpret exactly what occurs in this formulation of *model collapse*. To be precise, due to errors occurring from re-sampling the approximated distribution, each generation ends up corresponding to a new step in a random walk of model parameters. The risk that occurs in this model ends up diverging for a constant sample size at each generation. In order for the end distribution approximation to be accurate, and for the distance to be finite, the sampling rate M_i needs to increase superlinearly, *i.e.* one needs to collect increasingly more samples over time, perhaps quadratically. However, even in that case the expected distance after n steps remains non-zero and the only case in which it does in fact end up being 0 is when sampling is infinite at each step. Overall, this only shows us how far on average we go from the original distribution, but the process can only 'terminate' if the estimated variance at a certain generation becomes small enough, *i.e.* we effectively turn into a delta function.

Shown on Figures 4 and 5 are different runs of this process for different values of parameters of $\alpha_i, \beta_i, \gamma_i$ for different sample sizes, which was investigated numerically to see whether they can be enough to overcome *model collapse*, however even in those cases the changes are inevitable, although attenuated.

This scaling of divergence is the same as that of a single dimensional Gaussian random walk. We can further see the similarities in numerical experiments shown on Figure 3 for a range of different sample sizes, confirming these theoretical intuitions.

3.1.2 Multidimensional random variable

With the simple example of a 1D gaussian out of the way, we can now construct a lower bound on the distance of generation n distribution from the original and show that without superlinear sampling it similarly diverges in the limit of large n. A nice property of Wasserstein-2 distance is that Gaussians provide a universal lower bound for the Wasserstein distance [16]. In particular, for κ and ν probability measures on a Euclidean N-dimensional space with μ_κ and μ_ν means, Σ_κ and Σ_ν covariance matrices, we have that

$$\mathbb{W}_2^2(\kappa,\nu) \geq \|\mu_\kappa-\mu_\nu\|^2 + \mathrm{Tr}\left(\Sigma_\kappa+\Sigma_v-2\left(\Sigma_\kappa^{1/2}\Sigma_v\Sigma_\kappa^{1/2}\right)^{1/2}\right) \geq \|\mu_\kappa-\mu_\nu\|^2$$

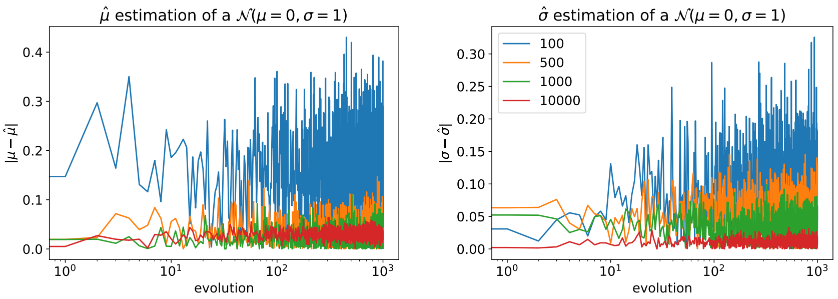

(a) Mean estimation (b) Standard Deviation

Fig. 4: Recursive fitting-sampling of a 1D Gaussian with different numbers of samples drawn. In this plot data get accumulated in a pool, from which a fixed sample is drawn. In other words, a model n gets data sampled, its output is mixed with data sampled from models $1 \dots n$, and then the mix gets sampled to fit the model $n + 1$. The uncertainty arising from all of the different modalities appearing in data causes the distribution parameters to jump around quite significantly.

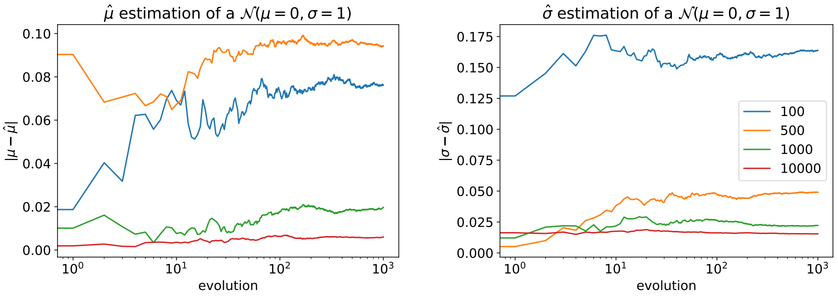

(a) Mean estimation (b) Standard Deviation

Fig. 5: Recursive fitting-sampling of a 1D Gaussian with different number of samples drawn. In this plot data are accumulated in a pool, all of which is used to fit a model. In other words, a model n gets data sampled, its output mixed with data sampled from models $1 \dots n$, and then the result is used to fit the model $n + 1$. Over time the variance in estimates reduces due to linear growth of data.

With this, instead of quantifying the distance exactly, we can instead lower bound it. The only limitation is that we are going to have to specify a functional approximation model. In order to achieve a $\mathbb{W}_2$ bound, we will be required to specify how the mean changes between generations. In the scenario where we only have access to the sample mean, we would approximate the mean of the next generation distribution as Equation (2). However, as more information arrives, or the model begins using it better, we may end up diverging from the sample mean. We would still require that the model have good performance, *i.e.* on average the mean estimate is the same. We will

also have to specify expected behaviour of the model over the the variance calculation, which once again will be chosen such that it averages out. Thus, we will adopt the following evolution over generations:

$$\mu_{i+1} = \frac{1}{M_i}\sum_j X_j^i + \varepsilon_{i+1} = \frac{\Sigma_i^{1/2}}{\sqrt{M_i}} T^{i+1} + \mu_i + \varepsilon_{i+1}; \quad \mathbb{E}_{X_j^i}(\Sigma_{i+1}) = \Sigma_i \tag{8}$$

where we define T^{i+1} to satisfy the equation above, *i.e.* $T^{i+1} = \frac{\Sigma_i^{-1/2}}{\sqrt{M_i}} \sum_j \left(X_j^i - \mu_i\right)$. With this normalisation T has mean 0 and covariance I_N and by the central limit theorem (CLT) we would have $T^{i+1}|\mu_i, \Sigma_i \xrightarrow{\mathcal{D}} \mathcal{N}(0, I_N)$, however the lower bound will not rely on this. To arrive at a lower bound for the risk, similar to that of Equation (6), we are going to have to make an assumption about the form of ε_{i+1}. Overall the result can be summarised in the following way:

Theorem 3.1 (Mean and Covariance-consistent Generalisation Error)**.** Let the model be updated between generations in a way such that its mean and covariance matrix are on average consistent, i.e. satisfy:

$$\mu_{i+1} = \frac{1}{M_i}\sum_j X_j^i + \varepsilon_{i+1}\left(X_j^i\right); \quad \mathbb{E}_{X_j^i}(\Sigma_{i+1}) = \Sigma_i; \quad \mathbb{E}[\varepsilon_{i+1}|\mu_i, \Sigma_i] = 0; \tag{9}$$

with ε_{i+1} denoting potential deviations from the usual unbiased estimators due to some implicit bias of the model or the optimisation routine. Then:

(a) If $\mathrm{Cov}(\varepsilon_{i+1}, \mu_{i+1}|\mu_i, \Sigma_i) = 0$:

$$\mathbb{E}\left[R_{W_2}^{n+1}\right] \geq \operatorname{Tr}\Sigma \sum_{i=0}^{n} \frac{1}{M_i} + \sum_{i=1}^{n+1} \mathbb{E}\left(\|\varepsilon_i\|^2\right) \tag{10}$$

(b) If exists $K > 0$ constant: $\|\varepsilon_{i+1}\| \leq K/M_i$:

$$\begin{aligned}\mathbb{E}\left[R_{W_2}^{n+1}\right] &\geq \operatorname{Tr}\Sigma \sum_{i=0}^{n} \frac{1}{M_i} + \sum_{i=1}^{n+1} \mathbb{E}\left(\|\varepsilon_i\|^2\right) - 2K\sqrt{N}\operatorname{Tr}\Sigma \sum_{i=0}^{n} \frac{1}{M_i^{3/2}} \\ &= \operatorname{Tr}\Sigma \sum_{i=0}^{n} \frac{1}{M_i} + \sum_{i=1}^{n+1} \mathbb{E}\left(\|\varepsilon_i\|^2\right) + \mathcal{O}(3/2).\end{aligned} \tag{11}$$

Let us first summarise the assumptions in more detail:

Assumptions:

[1] On average we can capture the mean to be the same as at the iteration prior:

$$\mathbb{E}[\varepsilon_{i+1}|\mu_i, \Sigma_i] = 0 \tag{12}$$

[2] Given all of X_j^i, epsilon must be constant, *i.e.* it is a function of only the data:

$$\varepsilon_{i+1} = \varepsilon_{i+1}\left(X_j^i\right) \tag{13}$$

In particular, it is dependent on μ_i and Σ_i only through the data.

[3A] The extra noise is orthogonal to the sample mean in the sense of random variables. This is effectively assuming that the noise does not contain any first moment information, *i.e.* we have:

$$\mathrm{Cov}(\varepsilon_{i+1}, T^{i+1}|\mu_i, \Sigma_i) = 0 \tag{14}$$

This may seem like a rather strong assumption, compared to the previous ones, however this property can be shown to hold true when imposing CLT on T^{i+1} in the limit of large M_i (note here that M_i can only be assumed to be **large**, and not infinite) and assuming that ε is strictly a function of moments higher than first. Furthermore, a property of this type is necessary to actually provide any information, since prior to it there would be no need to separate into an epsilon term and a sample mean term, since all could be absorbed into ε.

[3B] The extra noise is going to be assumed to be bounded and of the order larger than the sample mean deviation. To be precise we will have a constant K (not dependent on generation i), such that for all i:

$$\|\varepsilon_{i+1}\| \leq \frac{K}{M_i}. \tag{15}$$

Assumption 3A and Assumption 3B are two inherently different assumptions. Depending on the setting considered, one or the other may be more realistic. With these, we can now prove Theorem 3.1.

Proof of Theorem 3.1. With all the assumptions in place, we now have the following bound:

$$\mathbb{E}\left[R_{W_2}^{i+1}\right] \geq \mathbb{E}\left(\|\mu_{i+1} - \mu\|^2\right) \tag{16}$$

$$= \mathbb{E}\left(\|\mu_i - \mu\|^2\right) + \mathbb{E}\left(\|\varepsilon_{i+1}\|^2\right) + \frac{1}{M_i}\mathbb{E}\left((T^{i+1})^\top \Sigma_i (T^{i+1})\right) + \tag{17}$$

$$+ \frac{2}{\sqrt{M_i}}\mathbb{E}\left((\varepsilon_{i+1})^\top \Sigma_i^{1/2} T^{i+1} + (\mu_i - \mu)^\top \Sigma_i^{1/2} T^{i+1}\right) \tag{18}$$

$$= \mathbb{E}\left(\|\mu_i - \mu\|^2\right) + \frac{\mathrm{Tr}\,\Sigma}{M_i} + \mathbb{E}\left(\|\varepsilon_{i+1}\|^2\right) + \frac{2}{\sqrt{M_i}}\mathbb{E}\left((\varepsilon_{i+1})^\top \Sigma_i^{1/2} T^{i+1}\right) \tag{19}$$

(a) Now the only quantity to evaluate is

$$\frac{2}{\sqrt{M_i}}\mathbb{E}\left((\varepsilon_{i+1})^\top \Sigma_i^{1/2} T^{i+1}\right) = \frac{2}{\sqrt{M_i}} \int d\Sigma_i \, p(\Sigma_i) \,\mathrm{Tr}\left[\Sigma_i^{1/2}\,\mathrm{Cov}(\varepsilon_{i+1}, T^{i+1}|\Sigma_i)\right] = 0, \tag{20}$$

by Assumption 3A. Therefore, the overall bound would be similar to the Gaussian case, but with extra noise variance terms:

$$\mathbb{E}_{\mu_{n+1},\sigma^2_{n+1}}\left[R_{W_2}^{n+1}\right] \geq \mathrm{Tr}\,\Sigma\left(\frac{1}{M_0} + \frac{1}{M_1} + \cdots + \frac{1}{M_n}\right) + \sum_{i=1}^{n+1}\mathbb{E}\left(\|\varepsilon_i\|^2\right) \tag{21}$$

(b) Now using Assumption 3B, we can start from

$$\mathbb{E}\left[R_{W_2}^{i+1}\right] \geq \mathbb{E}\left(\|\mu_i - \mu\|^2\right) + \frac{\operatorname{Tr}\Sigma}{M_i} + \mathbb{E}\left(\|\varepsilon_{i+1}\|^2\right) + \frac{2}{\sqrt{M_i}}\mathbb{E}\left((\varepsilon_{i+1})^\top \Sigma_i^{1/2} T^{i+1}\right) \tag{22}$$

Similar to before, we need to evaluate (which we instead bound this time):

$$\begin{aligned}
\frac{2}{\sqrt{M_i}}\mathbb{E}\left((\varepsilon_{i+1})^\top \Sigma_i^{1/2} T^{i+1}\right) &= \frac{2}{\sqrt{M_i}} \int d\Sigma_i \, p(\Sigma_i) \operatorname{Tr}\left[\Sigma_i^{1/2} \operatorname{Cov}(\varepsilon_{i+1}, T^{i+1}|\Sigma_i)\right] \neq 0 \\
&\geq -\frac{2\sqrt{N}}{\sqrt{M_i}} \int d\Sigma_i \, p(\Sigma_i) \sqrt{\operatorname{Tr}\left[\Sigma_i \Sigma_{\epsilon_{i+1}}\right]} \\
&\geq -\frac{2\sqrt{N}}{\sqrt{M_i}} \sqrt{\mathbb{E}\left(\varepsilon_{i+1}^\top \Sigma_i \varepsilon_{i+1}\right)}, \\
&\geq -\frac{2\sqrt{N}}{\sqrt{M_i}} \sqrt{\frac{K^2 \operatorname{Tr}\Sigma}{M_i^2}} = \frac{-2K\sqrt{N}}{M_i\sqrt{M_i}} \sqrt{\operatorname{Tr}\Sigma},
\end{aligned}$$

where we used the Cauchy-Schwarz and Jensen inequalities. Note that this is far from optimal inequality, since instead of using the expected value of the largest eigenvalue, we instead bounded it by $\operatorname{Tr}\Sigma$. In particular, the per step bound is then:

$$\mathbb{E}\left[R_{W_2}^{i+1}\right] \geq \mathbb{E}\left(\|\mu_i - \mu\|^2\right) + \frac{\operatorname{Tr}\Sigma}{M_i} + \mathbb{E}\left(\|\varepsilon_{i+1}\|^2\right) - \frac{2K\sqrt{N}}{M_i\sqrt{M_i}}\sqrt{\operatorname{Tr}\Sigma}. \tag{23}$$

Without knowledge of the specific values of K, N or $\operatorname{Tr}\Sigma$, the best we can do is consider what this means for the bound as M_i becomes large. In particular, contribution from the last two terms will be of order at most $3/2$. As a result we recover a bound similar to all of the ones observed so far:

$$\mathbb{E}_{\mu_{n+1},\sigma^2_{n+1}}\left[R_{W_2}\right] \geq \operatorname{Tr}\Sigma\left(\frac{1}{M_0} + \frac{1}{M_1} + \cdots + \frac{1}{M_n}\right) + \mathcal{O}(3/2) \tag{24}$$

In particular, we find in the same way, that superlinear scaling would be required to minimise the lower bound on *model collapse* even in the case of more generic models of approximation. □

These results allow us to interpret the process of *model collapse*. To be precise, each generation corresponds to a new step in a random walk of model parameters. Due to errors occurring from re-sampling the approximated distribution, on average, the expected distance from the original distribution is non-zero. For constant sample size, the risk ends up diverging and in order for the limiting distribution to be accurate and the distance to be finite, the sampling rate M_i needs to increase superlinearly, *i.e.* one needs to collect superlinearly more samples over time, perhaps quadratically. However, even then, the expected distance remains non-zero unless sampling is infinite at each step. Overall, this only shows us how far on average we

depart from the original distribution, but the process can only 'terminate' if the estimated variance at a certain generation becomes small enough, *i.e.* we effectively turn into a delta function. In total, the message so far can be summarised as follows:

> *When learning on generational data, due to finite sampling, we are only able to* ***approximate*** *the original distribution. While on average we should recover the original distribution, the variance arising from this is non-zero. As a result, over the generations, the average distance of n'th generation from the original grows and can become infinite in the limit, since errors compound over time.*

3.1.3 Gaussian Model Collapse

In this subsection we briefly summarise the proof behind main theorem, which is mostly based on facts from discussions in the two subsections above.

Theorem 3.2 (Gaussian Model Collapse)**.** Let x_j^0 be fixed samples from some original distribution $\mathcal{D}_0$ (not necessarily Gaussian), with sample mean and covariance $(\hat{\mu}_0, \hat{\Sigma}_0)$ and $\hat{\Sigma}_0 \neq 0$. Assume X^n are fit recursively using the unbiased sample mean and variance estimators from the previous generation: $X_j^n|\mu_n,\ \Sigma_n \sim \mathcal{N}(\mu_n, \Sigma_n)$, with a fixed sample size. Then,

$$\mathbb{E}\left[\mathbb{W}_2^2\left(\mathcal{N}(\mu_n, \Sigma_n), \mathcal{D}_0\right)\right] \to \infty; \quad \Sigma_n \xrightarrow{\text{a.s.}} 0 \text{ as } n \to \infty,$$

where $\mathbb{W}_2^2$ denotes the Wasserstein-2 distance between the true distribution and the approximation at step n.

Proof. First note that $\mathbb{W}_2^2\left(\mathcal{N}(\mu_n, \Sigma_n), \mathcal{D}_0\right) \geq \|\mu_n - \mu_0\|^2$. Assume that $\hat{\mu}_0, \hat{\Sigma}_0$ are the estimates from samples x_j^0 from $\mathcal{D}_0$ and $\hat{\Sigma}_0 \neq 0$. Then, by triangle inequality,

$$\mathbb{W}_2^2\left(\mathcal{N}(\mu_n, \Sigma_n), \mathcal{D}_0\right) + \mathbb{W}_2^2\left(\mathcal{D}_0, \mathcal{N}(\hat{\mu}_0, \hat{\Sigma}_0)\right) \geq \frac{1}{2}\mathbb{W}_2^2\left(\mathcal{N}(\mu_n, \Sigma_n), \mathcal{N}(\hat{\mu}_0, \hat{\Sigma}_0)\right).$$

Therefore,

$$\mathbb{W}_2^2\left(\mathcal{N}(\mu_n, \Sigma_n), \mathcal{D}_0\right) \geq -\mathbb{W}_2^2\left(\mathcal{D}_0, \mathcal{N}(\hat{\mu}_0, \hat{\Sigma}_0)\right) + \frac{1}{2}\|\mu_n - \hat{\mu}_0\|^2.$$

Now, we are in the same setting as in the subsections above, and *e.g.* using the result from Theorem 3.1 with $\varepsilon = 0$, we have that

$$\mathbb{E}\left[\mathbb{W}_2^2\left(\mathcal{N}(\mu_n, \Sigma_n), \mathcal{D}_0\right)\right] \geq \frac{1}{2}\operatorname{Tr}\Sigma\left(\frac{1}{M_0} + \frac{1}{M_1} + \cdots + \frac{1}{M_n}\right) - \mathbb{W}_2^2\left(\mathcal{D}_0, \mathcal{N}(\hat{\mu}_0, \hat{\Sigma}_0)\right).$$

Since in our case M is assumed to be constant, we have

$$\mathbb{E}\left[\mathbb{W}_2^2\left(\mathcal{N}(\mu_n, \Sigma_n), \mathcal{D}_0\right)\right] \geq \frac{\operatorname{Tr}\hat{\Sigma}_0}{2M}n - \mathbb{W}_2^2\left(\mathcal{D}_0, \mathcal{N}(\hat{\mu}_0, \hat{\Sigma}_0)\right) \to \infty.$$

This proves the first claim. Proof of the second claim is heavily based on Alemohammad et al. [17], and is repeated here only for completeness. Once again, looking after the first step, such that only normal distributions are considered, we can write $X_n^i = \Sigma_{n-1}^{1/2} Z_n^i + \mu_{n-1}$, with $Z_n^i \sim \mathcal{N}(0, I)$, and denoting the sample mean as $\zeta_n = \frac{1}{M}\sum_{i=1}^M Z_n^i$. Now, noting that $\operatorname{Tr}\Sigma_n$ is a lower bounded submartingale and writing

$$\operatorname{Tr}\Sigma_n = \operatorname{Tr}\left[\left(\frac{1}{M-1}\sum_{i=1}^{M}\left(Z_n^i - \zeta_n\right)\left(Z_n^i - \zeta_n\right)^\top\right)\Sigma_{n-1}\right],$$

we see that $\operatorname{Tr}\Sigma_n \xrightarrow{\text{a.s.}} \sigma_\infty$ for some random variable σ_∞ by Doobs martingale convergence theorem [18]. Without loss of generality assume Σ_n is diagonal. Then, $\operatorname{Tr}\Sigma_n$ can be seen as a linear combination of N independent χ^2 random variables with $N-1$ degrees of freedom, and weights $\operatorname{diag}(\Sigma_{n-1})$. Writing $\operatorname{Tr}\Sigma_n = Q_n \operatorname{Tr}\Sigma_{n-1}$, where Q_n is a generalized χ^2 random variable with $N-1$ degrees of freedom and weights $\frac{\operatorname{diag}(\Sigma_{n-1})}{\operatorname{Tr}\Sigma_{n-1}}$, with $\mathbb{E}[Q_n|\Sigma_{n-1}] = 1$. Therefore, at least one of the weights is greater than $1/N$ for each n. Therefore, for any $0 < \epsilon < 1$, there exists $c > 0$ such that $\Pr(|Q_n - 1| > \epsilon) > c$. Since $|Q_n - 1| > \epsilon$ infinitely often with probability one, $\sigma_\infty = 0$ is the only random variable that satisfies $\lim_{n\to\infty}\operatorname{Tr}\Sigma_0\prod_{j=1}^n Q_j = \sigma_\infty$, and as all matrix norms are equivalent, $\Sigma_n \xrightarrow{\text{a.s.}} 0$. □

3.2 Regularised density estimation in RKHS

3.2.1 In-depth setup

In this subsection we provide the theoretical framework for the density estimation problem in a RKHS. The setting closely follows that presented in Kazashi and Nobile [12]. Let $(\mathcal{X}, \mathcal{B}, \mu)$ be a measure space, and let $(\mathcal{N}_K, \langle\cdot,\cdot\rangle_K, \|\cdot\|_K)$ denote the RKHS associated with the positive definite kernel $K : \mathcal{X}\times\mathcal{X}\to\mathbb{R}$, i.e. $K(x, x') = K(x', x)$ for all $x, x' \in \mathcal{X}$, and for any $m \in \mathbb{N}, t_j, t_k \in \mathbb{R}$, and $x_j, x_k \in \mathcal{X}, j, k = 0, \ldots, m$, we have $\sum_{j,k=0}^m t_j K(x_j, x_k) t_k \geq 0$. This kernel is allowed to be unbounded, but we assume $\int_{\mathcal{X}}\sqrt{K(x,x)}\mathrm{d}\mu(x) < \infty$ and $\int_{\mathcal{X}} K(x,x)\mathrm{d}\mu(x) < \infty$. We assume that K admits a representation

$$K(x, x') = \sum_{\ell=0}^{\infty}\beta_\ell\varphi_\ell(x)\varphi_\ell(x') \qquad x, x' \in \mathcal{X},$$

with a positive sequence $(\beta_\ell)_{\ell=0}^\infty \subset (0,\infty)$ converging to 0 , and a complete orthonormal system $\left\{\sqrt{\beta_\ell}\varphi_\ell\right\}$ of $\mathcal{N}_K$ such that the series is absolutely (point-wise) convergent and that $\{\varphi_\ell\}$ is an orthonormal system of $L^2_\mu(\mathcal{X})$. Then, the inner product for $\mathcal{N}_K$ may be represented by

$$\langle f, g\rangle_K = \sum_{\ell=0}^{\infty}\frac{\langle f,\varphi_\ell\rangle_{L^2_\mu(\mathcal{X})}\langle g,\varphi_\ell\rangle_{L^2_\mu(\mathcal{X})}}{\beta_\ell},$$

where using the notation $\langle u, w\rangle_{L^2_\mu(\mathcal{X})} := \int_{\mathcal{X}} u(x)v(x)\mathrm{d}\mu(x)$ for the $L^2_\mu(\mathcal{X})$-inner product. Similar to Kazashi and Nobile [12] we introduce the notation

$$\langle u, w\rangle_\lambda := \langle u, w\rangle_{L^2_\mu(\mathcal{X})} + \lambda\langle u, w\rangle_K \qquad \text{for } u, w \in \mathcal{N}_K,$$

where $\lambda > 0$ is a parameter and the bilinear form $\langle \cdot, \cdot \rangle_\lambda$ is an inner product on $\mathcal{N}_K$. We further define the integral operator $T_K : L^2_\mu(\mathcal{X}) \to L^2_\mu(\mathcal{X})$ by

$$T_K g := \int_{\mathcal{X}} K(\cdot, x) g(x) \mathrm{d}\mu(x), \quad g \in L^2_\mu(\mathcal{X}).$$

Let $Y_1^0, \ldots, Y_M^0 : \Omega \to \mathcal{X}$ be independent random variables that follow the distribution defined by a density $y \in \mathcal{N}_K$ with respect to μ. Similar to Kazashi and Nobile [12], we are approximating y of the form $y_\theta(\cdot) \approx \sum_{n=1}^N a_n K(x_n, \cdot)$ for $X = \{x_1, \ldots, x_N\} \subset \mathcal{X}$ a set of specifically chosen points, defining the approximation space $V_N := \operatorname{span}\{K(x_j, \cdot) \mid j = 1, \ldots, N\}$. Unlike Kazashi and Nobile [12], we are interested in a constrained problem, which provides us with a way to select the regularisation parameter. The idealised regularised density estimation would consist of solving the following constrained optimistaion problem:

$$\arg\min_{u \in V_N} \quad \frac{1}{2}\|u\|_K^2 \quad \text{s.t.} \quad \frac{1}{2}\|u - y\|^2_{L^2_\mu(\mathcal{X})} \leq \frac{\epsilon}{2}.$$

The Karush-Kuhn-Tucker (KKT) condition for this problem yields,

$$\begin{aligned} &\arg\min_{u \in V_N} \frac{\lambda}{2}\|u - y\|^2_{L^2_\mu(\mathcal{X})} + \frac{1}{2}\|u\|_K^2 \\ &\text{s.t. } \lambda \geq 0, \qquad \|u - y\|^2_{L^2_\mu(\mathcal{X})} \leq \epsilon \\ &\qquad \lambda\left(\|u - y\|^2_{L^2_\mu(\mathcal{X})} - \epsilon\right) = 0. \end{aligned}$$

However, in the density estimation setting we do not have access to the true density, but just samples. Therefore the problem we consider is a sample approximation of the ideal problem:

$$\begin{aligned} f_n^* &= \arg\min_{u \in V_N} \quad \frac{1}{2}\|u\|_K^2 \\ \text{s.t. } &\frac{1}{2}\|u\|^2_{L^2_\mu(\mathcal{X})} - \frac{1}{M}\sum_{m=1}^M u\left(Y_m^n\right) + \frac{1}{2}\left[\hat{A}^{-1/2} k_{Y^n}\right]^\top \left[\hat{A}^{-1/2} k_{Y^n}\right] \leq \frac{\epsilon_n}{2}, \end{aligned}$$

where we define $\hat{A}_{jk} \equiv \langle K(x_j, \cdot), K(x_k, \cdot) \rangle_{L^2_\mu(\mathcal{X})}$. In this case, the last term acts as a proxy for $\|y\|^2_{L^2_\mu(\mathcal{X})}$. With this, we can follow Mobahi et al. [13] and in analogy write down the the Karush-Kuhn-Tucker (KKT) condition for this problem,

$$\begin{aligned} f_n^* &= \arg\min_{u \in V_N} \lambda_n \left(\frac{1}{2}\|u\|^2_{L^2_\mu(\mathcal{X})} - \frac{1}{M}\sum_{m=1}^M u\left(Y_m^n\right)\right) + \frac{1}{2}\|u\|_K^2 \\ \text{s.t. } \lambda_n \geq 0 \quad , \quad &\frac{1}{2}\|u\|^2_{L^2_\mu(\mathcal{X})} - \frac{1}{M}\sum_{m=1}^M u\left(Y_m^n\right) \leq \epsilon_n - \frac{1}{2}\left[\hat{A}^{-1/2} k_{Y^n}\right]^\top \left[\hat{A}^{-1/2} k_{Y^n}\right] \end{aligned}$$

$$\lambda_n\left(\frac{1}{2}\|u\|^2_{L^2_\mu(\mathcal{X})}-\frac{1}{M}\sum_{m=1}^{M}u\left(Y^n_m\right)-\left(\frac{\epsilon_n}{2}-\frac{1}{2}\left[\hat{A}^{-1/2}k_{Y^n}\right]^\top\left[\hat{A}^{-1/2}k_{Y^n}\right]\right)\right)=0.$$

Then, in analogy, when $\left[\hat{A}^{-1/2}k_{Y^n}\right]^\top\left[\hat{A}^{-1/2}k_{Y^n}\right]\leq\epsilon_n$, then f^* has trivial solution $f^*=0$, which is referred to as collapse. And in what follows, the more interesting case is investigated, *i.e.* $\left[\hat{A}^{-1/2}k_{Y^n}\right]^\top\left[\hat{A}^{-1/2}k_{Y^n}\right]>\epsilon_n$, with:

$$\left[\hat{A}^{-1/2}k_{Y^n}\right]^\top\left[\hat{A}^{-1/2}k_{Y^n}\right]>\epsilon_n \iff \lambda_n>0.$$

From now on we let $c_n=1/\lambda_n$ and write down the following approximate and ideal problems:

$$f:=\underset{v\in V_N}{\arg\min}\left[\frac{1}{2}\|v-y\|^2_{L^2_\mu(\mathcal{X})}+\frac{c}{2}\|v\|^2_K\right].$$

$$f^*_n:=\underset{v\in V_N}{\arg\min}\left[\frac{1}{2}\|v\|^2_{L^2_\mu(\mathcal{X})}+\frac{c_n}{2}\|v\|^2_K-\frac{1}{M}\sum_{m=1}^{M}v\left(Y^n_m\right)\right].$$

For these, we can write down the exact solutions. For the second, that is equivalent to finding $f^*_n\in V_N$ such that

$$\langle f^*_n,v\rangle_{c_n}=\frac{1}{M}\sum_{m=1}^{M}v\left(Y^n_m\right)\qquad\text{for all } v\in V_N. \tag{25}$$

Reader is referred to Kazashi and Nobile [12], Ciarlet [19] for the equivalence and well-posedness discussion. In particular, defining $\hat{A}_{jk}\equiv\langle K\left(x_j,\cdot\right),K\left(x_k,\cdot\right)\rangle_{L^2_\mu(\mathcal{X})}$, $A_{jk}\equiv K\left(x_j,x_k\right)$, and further let $P=\hat{A}^{-1/2}A\hat{A}^{-1/2}$, with P symmetric positive definite, and let $k(\cdot)_j=K\left(x_j,\cdot\right)$, and similarly $k_{(Y)_j}=\frac{1}{M}\sum_{m=1}^{M}K\left(x_j,Y_m\right)$, for $j=1,\ldots,N$. Then, the variational problems satisfy

$$f^*_n=k(\cdot)^\top\hat{A}^{-1/2}(c_nP+I)^{-1}\hat{A}^{-1/2}k_Y,$$

$$f=k(\cdot)^\top\hat{A}^{-1/2}(c_nP+I)^{-1}\hat{A}^{-1/2}[T_Ky](x_{1:N})$$

for $Y^n\sim y\,\mathrm{d}\mu$ and by Kazashi and Nobile [12], we have that $\mathbb{E}\left[f^*_n\right]=f$.

3.2.2 Unrolling the model collapse

Now, we would like to understand what happens when we perform this approximation repeatedly. As the x_i are fixed, the result turns out to be very close to Mobahi et al. [13]. In particular, for this subsection we will denote samples at generation n as Y^n, and letting $f^*_{-1}\equiv y$, we have the following:

$$Y^{n+1}\sim f^*_n=k(\cdot)^T\hat{A}^{-1/2}(c_nP+I)^{-1}\hat{A}^{-1/2}k_{Y^n}.$$

Now, in order to proceed we need to make sure that one of ϵ_n or c_n is constant, while the other implicitly becomes a random variable, and can only be treated as such. For simplicity we choose c_n to be constant and ϵ_n defined implicitly, while c_n is chosen such that, in expectation $\mathbb{E}(\epsilon_n) = \epsilon$. In this case, we have:

$$\mathbb{E}[f_n^*] = k(\cdot)^\top \hat{A}^{-1/2} \left[\prod_{i=0}^{n} (c_i P + I)^{-1} \right] \hat{A}^{-1/2} [T_K y](x_{1:N}).$$

This follows from iteratively using Equation (25) similar to Kazashi and Nobile [12], e.g. for $n = 2$:

$$\begin{aligned}
&\langle \mathbb{E}_{Y^2,Y^1,Y^0}[f_2^*], v \rangle_{c_2} \\
&= \int_{z^1} \mathbb{E}_{Y^2|Y^1=z^1}[v(Y^2)] d\mu(z^1) \int_{z^0} f_0^*|_{Y^0=z^0}(z^1) y(z^0) d\mu(z^0) = \\
&= \int_x d\mu(x) v(x) k(x)^\top \int_{z^1,z^0} \hat{A}^{-1/2} (c_1 P + I)^{-1} \hat{A}^{-1/2} \\
&\qquad \cdot \left[k_{z_1} k(z^1)^T \hat{A}^{-1/2} (c_0 P + I)^{-1} \hat{A}^{-1/2} k_{z^0} d\mu(z^1) y(z^0) d\mu(z^0) \right] \\
&= \int_x d\mu(x) v(x) k(x)^\top \hat{A}^{-1/2} (c_1 P + I)^{-1} (c_0 P + I)^{-1} \hat{A}^{-1/2} [T_K y](x_{1:N}).
\end{aligned}$$

As P is symmetric positive definite, we can write it as $P = V^\top D V$, with D diagonal. Let $d_{\min}$ and $d_{\max}$ denote its smallest and largest eigenvalues, and let $B_n = \prod_{i=0}^n (c_i D + I)^{-1}$, and $k_{z_Y} = V \hat{A}^{-1/2} k_Y$. Now, the only thing missing is the relevant bounds on c_i, which would allow us to directly utilise results in [13]. In our case they are defined through:

$$\left(\frac{1}{2} \|f_n^*\|^2_{L^2_\mu(\mathcal{X})} - \frac{1}{M} \sum_{m=1}^{M} f_n^*(Y_m^n) - \left(\frac{\epsilon_n}{2} - \frac{1}{2} \left[\hat{A}^{-1/2} k_{Y^n} \right]^\top \left[\hat{A}^{-1/2} k_{Y^n} \right] \right) \right) = 0.$$

Now, based on Equation (25), we need to evaluate $\frac{1}{2}\|f_n^*\|^2_{L^2_\mu(\mathcal{X})}$ and $\frac{1}{2}\|f_n^*\|^2_K$:

$$\|f_n^*\|^2_{L^2_\mu(\mathcal{X})} = k_{Y^n}^\top \hat{A}^{-1/2} (c_n P + I)^{-2} \hat{A}^{-1/2} k_{Y^n}.$$

$$\|f_n^*\|^2_K = k_{Y^n}^\top \hat{A}^{-1/2} P (c_n P + I)^{-2} \hat{A}^{-1/2} k_{Y^n}.$$

This, in turn lets us calculate value of ϵ_n:

$$\begin{aligned}
\epsilon_n &= -\|f_n^*\|^2_{L^2_\mu(\mathcal{X})} - 2c_n \|f_n^*\|^2_K + \left[\hat{A}^{-1/2} k_Y \right]^\top \left[\hat{A}^{-1/2} k_Y \right] \\
&= k_{Y^n}^\top \hat{A}^{-1/2} c_n^2 P^2 (c_n P + I)^{-2} \hat{A}^{-1/2} k_{Y^n} \\
&= k_{z_{Y^n}}^\top \operatorname{diag}\left(\frac{c_n^2 d_j^2}{(c_n d_j + 1)^2} \right) k_{z_{Y^n}}
\end{aligned}$$

$$= k_{z_{Y^n}}^\top \operatorname{diag}\left(\frac{1}{(\frac{1}{c_n d_j}+1)^2}\right) k_{z_{Y^n}}.$$

Thus, in analogy to Mobahi et al. [13], we can derive the following upper and lower bounds on c_n, which we evaluate here using the expected values, based on the discussion above:

$$\frac{\sqrt{\mathbb{E}(\epsilon_n)}}{d_{max}\left(\sqrt{\mathbb{E}(k_{z_{Y^n}}^\top k_{z_{Y^n}})}-\sqrt{\mathbb{E}(\epsilon_n)}\right)} \leq c_n \leq \frac{\sqrt{\mathbb{E}(\epsilon_n)}}{d_{min}\left(\sqrt{\mathbb{E}(k_{z_{Y^n}}^\top k_{z_{Y^n}})}-\sqrt{\mathbb{E}(\epsilon_n)}\right)}$$

Therefore,

$$c_n = \frac{\sqrt{\mathbb{E}(\epsilon_n)}}{\alpha_n\left(\sqrt{\mathbb{E}(k_{z_{Y^n}}^\top k_{z_{Y^n}})}-\sqrt{\mathbb{E}(\epsilon_n)}\right)}.$$

For $\alpha_n \in [d_{\min}, d_{\max}]$. Now, as mentioned above, we set $\mathbb{E}(\epsilon_n) = \epsilon$, *i.e.* the desired accuracy is achieved in expectation. Thus, we only need to calculate $\mathbb{E}(k_{z_{Y^n}}^\top k_{z_{Y^n}})$:

$$\mathbb{E}\left(\left[\hat{A}^{-1/2}k_Y\right]^\top\left[\hat{A}^{-1/2}k_Y\right]\right) = \sum_{ij}\hat{A}_{ij}^{-1}\left(\mathbb{E}\left(K(x_i,Y)\right)\mathbb{E}\left(K(x_j,Y)\right)+\frac{1}{M}\operatorname{Cov}\left(K(x_i,Y),K(x_j,Y)\right)\right).$$

Now in order to simplify our calculations, we assume $M \to \infty$ in order to be able to drop the second term. Now this is possible, due to the second term being bounded, as a product of two kernels is a kernel. From now on working in the limit, based on the previous calculations, we know that:

$$\mathbb{E}[\hat{A}^{-1/2}k_{Y^n}] = \left[\prod_{i=0}^{n-1}(c_i P + I)^{-1}\right]\hat{A}^{-1/2}[T_K y](x_{1:N}), \tag{26}$$

and thus,

$$\begin{aligned}
&\mathbb{E}\left(K(x_{1:N},Y^n)\right)^\top \hat{A}^{-1}\mathbb{E}\left(K(x_{1:N},Y^n)\right)\\
&\quad= \left(\hat{A}^{-1/2}[T_K y](x_{1:N})\right)^\top\left[\prod_{i=0}^{n-1}(c_i P + I)^{-2}\right]\hat{A}^{-1/2}[T_K y](x_{1:N})\\
&\quad= \left(V\hat{A}^{-1/2}[T_K y](x_{1:N})\right)^\top \operatorname{diag}\left(\prod_{i=0}^{n-1}\frac{1}{(c_i d_j+1)^2}\right)V\hat{A}^{-1/2}[T_K y](x_{1:N}).
\end{aligned}$$

We use the following concise notation:

$$v_0 = V\hat{A}^{-1/2}[T_K y](x_{1:N}),$$

$$v_n = (c_{n-1}D + I)^{-1} v_{n-1} = \left(\frac{1}{\alpha_n \left(\frac{\|v_{n-1}\|}{\sqrt{\epsilon}} - 1 \right)} D + I \right)^{-1} v_{n-1},$$

with which we have $\sqrt{\mathbb{E}(k_{z_{Y^n}}^\top k_{z_{Y^n}})} = \|v_n\|$. This in turn directly coincides with that in Mobahi et al. [13], replacing D with its inverse. To be precise, the following follows exactly from Mobahi et al. [13]:

Proposition 3.1. For any $n \geq 0$, if $\|v_i\| > \sqrt{\epsilon}$ for $i = 0, \ldots, n$, then, $\|v_n\|$ is decreasing wrt n and

$$\|v_n\| \geq a^n(\kappa) \|v_0\| - \sqrt{\epsilon} b(\kappa) \frac{a^n(\kappa) - 1}{a(\kappa) - 1},$$

where,

$$a(x) \equiv \frac{(r_0 - 1)^2 + x(2r_0 - 1)}{(r_0 - 1 + x)^2}$$
$$b(x) \equiv \frac{r_0^2 x}{(r_0 - 1 + x)^2}$$
$$r_0 \equiv \frac{1}{\sqrt{\epsilon}} \|v_0\| \quad , \quad \kappa \equiv \frac{d_{\max}}{d_{\min}}.$$

Which in the same way provides a lower bound, until which no collapse occurs:

Proposition 3.2. Starting from $\|v_0\| > \epsilon$, meaningful (non-collapsing solution) training on generational data is possible at least for $\underline{n}$ generations,

$$\underline{n} \equiv \frac{\frac{\|v_0\|}{\sqrt{\epsilon}} - 1}{\kappa}.$$

And during this, the effect can be viewed as sparsification of the underlying function, in the following sense. The expected density is:

$$\mathbb{E}[f_n^*] = k(\cdot)^\top \hat{A}^{-1/2} V^\top \left[\prod_{i=0}^{n} (c_i D + I)^{-1} \right] V \hat{A}^{-1/2} [T_K y](x_{1:N}),$$

and denoting $B_n = \prod_{i=0}^{n} (c_i D + I)^{-1}$, we have the following:

Proposition 3.3. Starting from $\|v_0\| > \epsilon$ and for $n \leq \frac{\frac{\|v_0\|}{\sqrt{\epsilon}} - 1}{\kappa}$. Then for d_j and d_k any pair of diagonals of D with $d_k < d_j$, the following inequality holds:

$$\frac{B_{n-1}[k,k]}{B_{n-1}[j,j]} \geq \left(\frac{\frac{\|v_0\|}{\sqrt{\epsilon}} - 1 + \frac{d_j}{d_{\max}}}{\frac{\|v_0\|}{\sqrt{\epsilon}} - 1 + \frac{d_k}{d_{\max}}} \right)^n.$$

In total, the message so far can be summarised as follows:

> *When regularising density estimation with generational data, due to functional approximation errors, the approximated distribution gets sparser over generations, even in the limit of infinite data. As such, even with perfect information, we do not recover the original distribution, and over time model collapse occurs.*

3.3 Absorbing Markov Chain

The subsection explains a well-known fact about absorbing Markov chains, that they converge to an absorbing state with probability one. Assume that $\mathbf{X}^m$ form a Markov chain. In order to reason about this chain we need to consider the transition probabilities. In general, these correspond to our functional approximation scheme. Due to the stochastic nature of the Markov chain, we expect to have the variance go up and down. But as the variance decreases, the newly sampled data, due to its finiteness, will be more concentrated, leading in the limit to a set of *i.e.* a delta functions. This argument assumes that the approximation scheme is good and can converge to delta functions. If not, the errors in approximation may prevent the propagation of errors in stochasticity.

As discussed in the previous section, we can model the process of repeated 'sampling' and 'fitting' as a Markov chain. In this subsection, we explain how such a process can converge to a stationary state *i.e.* the absorbing state of a Markov Chain. In this derivation we follow Allan Yashinski [1]. Suppose we have an absorbing Markov Chain with r transient states $t_1, \dots, t_r$ and s absorbing states $a_1, \dots, a_s$. The whole Markov chain has $r + s$ states, ordered as follows: $t_1, \dots, t_r, a_1, \dots, a_s$. The transition matrix is then defined as

$$T = \begin{bmatrix} Q & 0_{r\times s} \\ R & I_s \end{bmatrix}, \tag{27}$$

where

- Q is an $r \times r$ matrix holds the probabilities of moving from a transient state to another transient state
- R is an $s \times r$ matrix which holds the probabilities of moving from a transient state to an absorbing state.
- $0_{r\times s}$ is the $r \times s$ matrix of all 0's. There 0's represent the probabilities of moving from an absorbing state to a transient state (which is impossible by definition).
- I_s holds the probabilities of transitioning between the absorbing states. As transition is impossible, this is just the $s \times s$ identity matrix.

We are interested in $\lim_{k\to\infty} T^k(\mathbf{X}_0)$. For a given k, the matrix becomes

$$T^k = \begin{bmatrix} Q^k & 0_{r\times s} \\ R + RQ + \cdots + RQ^{k-1} & I_s \end{bmatrix} = \begin{bmatrix} Q^k & 0_{r\times s} \\ R\sum_{i=0}^{k-1} Q^i & I_s \end{bmatrix}. \tag{28}$$

Finally, for an absorbing Markov chain with $T = \begin{bmatrix} Q & 0_{r\times s} \\ R & I_s \end{bmatrix}$,

[1] www.math.umd.edu/~immortal/MATH401/book/ch_absorbing_markov_chains.pdf

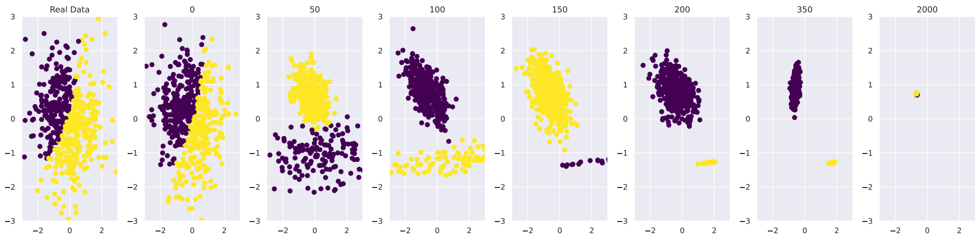

Fig. 6: An examples of GMM fitting data at iterations $\{0, 50, 100, 150, 200, 350, 2000\}$. At first the model fits data very well as is shown on the left; yet even at generation 50 the perception of the underlying distribution completely changes. At generation 2000 it converges to a state with very little variance. GMM is sampled a thousand times.

we have $\lim_{k\to\infty} T^k = \begin{bmatrix} 0_{r\times r} & 0_{r\times s} \\ R(I_r - Q)^{-1} & I_s \end{bmatrix}$.

Since in the limit the transition probabilities to transient states are zero, we end up converging to absorbing states and staying there. In the case of discrete distributions, where we can perfectly approximate a zero-variance dataset (*i.e.* a delta function), the absorbing states are delta functions centered at any non-zero probability point from the original distribution. In practice, we would like to know the expected number of steps before being absorbed, which may be large. But without knowing our fitting procedure it is impossible to calculate the matrix Q and therefore the average length of time before collapse.

4 GMM and VAE experiments

4.1 Training from scratch with GMMs and VAEs

Gaussian Mixture Models. In this subsection we evaluate the performance of Gaussian Mixture Models (GMM) [20]. The underlying task here is that a given GMM tries to separate two artificially-generated Gaussians. Figure 6 shows the progression of the GMM fitting process over time. The left-most plot shows the original two Gaussians with the ground truth labels. The next plot shows the GMM fitted on the original data with no cross-generational data used *i.e.* $\alpha_i = \gamma_i = 0$, where the error is minimal. Yet, within 50 iterations of re-sampling we arrive to a point where the underlying distribution is mis-perceived. The performance worsens over time and by iteration 2000 we arrive at a point estimate of the distribution with very little variance. The L2 distance between the original GMM and its descendants is plotted in Figure 8.

Variational Autoencoders. In this subsection we turn to Variational Autoencoders (VAE). As before, we train an autoencoder on an original data source, in this case MNIST [21], which we later sample. Here, we generate latents from a Gaussian distribution which are then used by the decoder to generate data for the subsequent generation. Figure 7 on the left shows an example of generated data using the setting described by Kingma and Welling [22].

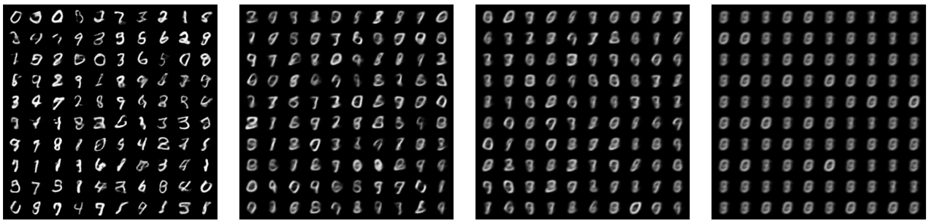

(a) Original model (b) Generation 5 (c) Generation 10 (d) Generation 20

Fig. 7: Random latent reconstructions from VAEs. No training data comes from the original distribution. Over the generations, different modes of the original distribution get entangled and generated data starts looking unimodal.

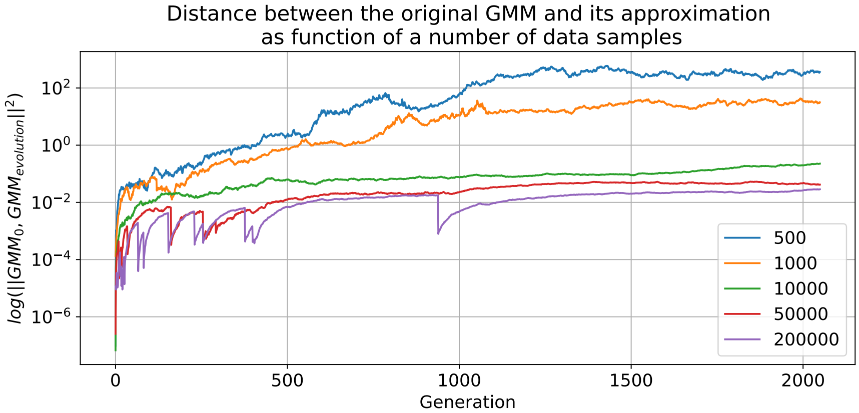

Fig. 8: Progressive fitting of a GMM with different number of samples. On the y-axis is shown the logarithm of $L2$ distance between the two GMM distributions. Over the generations the distance begins to grow and can become quite large. The jumps in the distance for large sample sizes occur due to the fixed number of iterations and precision for the expectation maximization algorithm.

Having performed the process a number of times we arrive at a representation that has very little resemblance of the original classes learned from data. On the right, one sees the generated images from generation 20, which appear to be a mix of all of the different digits. Interestingly, the original encoder perceives the generated data from its descendant with ever-growing confidence – the encoder places such data closer and closer to the mean. Figure 1 shows the density of the latent representation of the original model when presented with data generated by its descendants. As with single-dimensional Gaussians, tails disappear over time and all of the density shifts towards the mean. We find that such degradation happens consistently over multple independent runs.

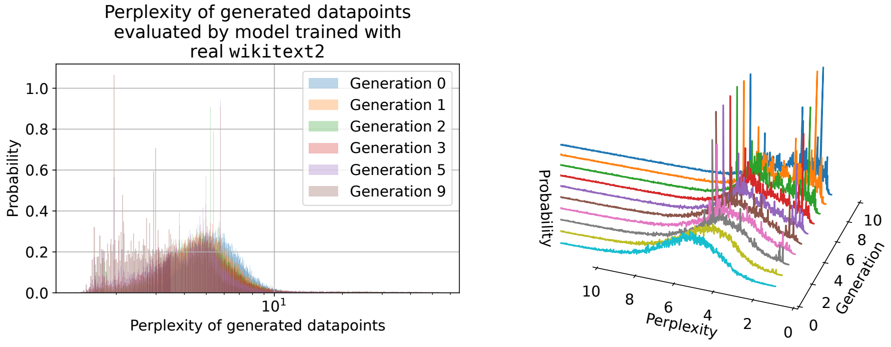

(a) Overlaid histograms

(b) 3D view

Fig. 9: Histogram of perplexities of each individual data training sequence produced by different generations as is evaluated by the very first model trained with the real data. Over the generations models tend to produce samples that the original model (trained with real data) is more likely to produce. At the same time, a much longer tail appears for later generations – later generations start producing samples that would never be produced by the original model *i.e.* they start misperceiving reality based on errors introduced by their ancestors. Models here are explicitly forced to not repeat sequences with a penalty of 2.0.

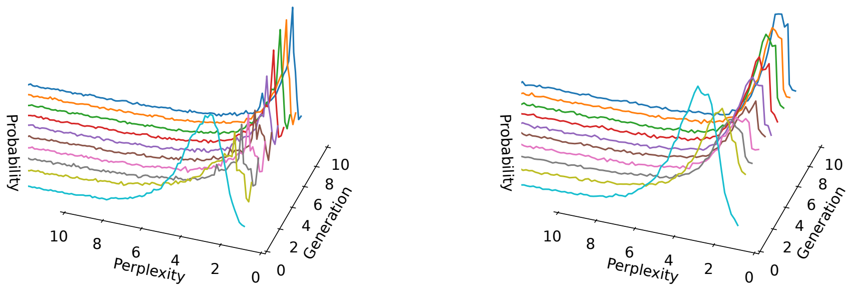

(a) Figure 1b from main text in 3D. No data preserved.

(b) Figure 1c from main text in 3D. 10% original data preserved.

Fig. 10: Histogram of perplexities of each individual data training sequence produced by different generations as is evaluated by the very first model trained with the real data. Over the generations models tend to produce samples that the original model (trained with real data) is more likely to produce. At the same time, a much longer tail appears for later generations – later generations start producing samples that would never be produced by the original model *i.e.* they start misperceiving reality based on errors introduced by their ancestors.

References

[1] Ven, G.M., Tolias, A.S.: Three scenarios for continual learning. arXiv preprint arXiv:1904.07734 (2019)

[2] Aljundi, R., Kelchtermans, K., Tuytelaars, T.: Task-free continual learning. In: Proceedings of the IEEE/CVF Conference on Computer Vision and Pattern Recognition, pp. 11254–11263 (2019)

[3] Kirkpatrick, J., Pascanu, R., Rabinowitz, N., Veness, J., Desjardins, G., Rusu, A.A., Milan, K., Quan, J., Ramalho, T., Grabska-Barwinska, A., *et al.*: Overcoming catastrophic forgetting in neural networks. Proceedings of the national academy of sciences **114**(13), 3521–3526 (2017)

[4] Aljundi, R., Babiloni, F., Elhoseiny, M., Rohrbach, M., Tuytelaars, T.: Memory aware synapses: Learning what (not) to forget. In: Proceedings of the European Conference on Computer Vision (ECCV), pp. 139–154 (2018)

[5] Li, Z., Hoiem, D.: Learning without forgetting. IEEE transactions on pattern analysis and machine intelligence **40**(12), 2935–2947 (2017)

[6] Biggio, B., Nelson, B., Laskov, P.: Poisoning attacks against support vector machines. In: Proceedings of the 29th International Coference on International Conference on Machine Learning. ICML'12, pp. 1467–1474. Omnipress, Madison, WI, USA (2012)

[7] Gu, T., Dolan-Gavitt, B., Garg, S.: Badnets: Identifying vulnerabilities in the machine learning model supply chain. arXiv preprint arXiv:1708.06733 (2017)

[8] Radford, A., Kim, J.W., Hallacy, C., Ramesh, A., Goh, G., Agarwal, S., Sastry, G., Askell, A., Mishkin, P., Clark, J., *et al.*: Learning transferable visual models from natural language supervision. In: International Conference on Machine Learning, pp. 8748–8763 (2021). PMLR

[9] Brown, T., Mann, B., Ryder, N., Subbiah, M., Kaplan, J.D., Dhariwal, P., Neelakantan, A., Shyam, P., Sastry, G., Askell, A., *et al.*: Language models are few-shot learners. Advances in neural information processing systems **33**, 1877–1901 (2020)

[10] Carlini, N., Terzis, A.: Poisoning and backdooring contrastive learning. In: International Conference on Learning Representations (2022). https://openreview.net/forum?id=iC4UHbQ01Mp

[11] Carlini, N., Jagielski, M., Choquette-Choo, C., Paleka, D., Pearce, W., Anderson, H., Terzis, A., Thomas, K., Tramèr, F.: Poisoning web-scale training datasets is practical. In: 2024 IEEE Symposium on Security and Privacy (SP), pp. 175–175. IEEE Computer Society, Los Alamitos, CA, USA (2024). https://doi.

org/10.1109/SP54263.2024.00179 . https://doi.ieeecomputersociety.org/10.1109/SP54263.2024.00179

[12] Kazashi, Y., Nobile, F.: Density estimation in rkhs with application to korobov spaces in high dimensions. SIAM Journal on Numerical Analysis **61**(2), 1080–1102 (2023)

[13] Mobahi, H., Farajtabar, M., Bartlett, P.: Self-distillation amplifies regularization in hilbert space. Advances in Neural Information Processing Systems **33**, 3351–3361 (2020)

[14] Fischer, A., Gaunt, R., Sarantsev, A.: The variance-gamma distribution: A review. Statistical Science (2024)

[15] Cochran, W.G.: The distribution of quadratic forms in a normal system, with applications to the analysis of covariance. Mathematical Proceedings of the Cambridge Philosophical Society **30**(2), 178–191 (1934) https://doi.org/10.1017/S0305004100016595

[16] Gelbrich, M.: On a formula for the l2 wasserstein metric between measures on euclidean and hilbert spaces. Mathematische Nachrichten **147**(1), 185–203 (1990)

[17] Alemohammad, S., Casco-Rodriguez, J., Luzi, L., Humayun, A.I., Babaei, H., LeJeune, D., Siahkoohi, A., Baraniuk, R.: Self-consuming generative models go MAD. In: The Twelfth International Conference on Learning Representations (2024). https://openreview.net/forum?id=ShjMHfmPs0

[18] Williams, D.: Probability with martingales (1991)

[19] Ciarlet, P.G.: Linear and nonlinear functional analysis with applications (2013)

[20] Reynolds, D.A., et al.: Gaussian mixture models. Encyclopedia of biometrics **741**(659-663) (2009)

[21] Y., L., C, C., C.J.C, B.: The MNIST Database of Handwritten Digits (1998). http://yann.lecun.com/exdb/mnist/

[22] Kingma, D.P., Welling, M.: Auto-Encoding Variational Bayes (2022)
