# Supplementary material for: AI models collapse when trained on recursively generated data
Source: Nature. 2024 Jul 24;631(8022):755–9. doi: 10.1038/s41586-024-07566-y (PMC11269175; doi:10.1038/s41586-024-07566-y)
Supplement: Supplementary file 2 — Supplementary Data [file 41586_2024_7566_MOESM2_ESM.zip › images/GMM/l2_example_lowtol.pdf]

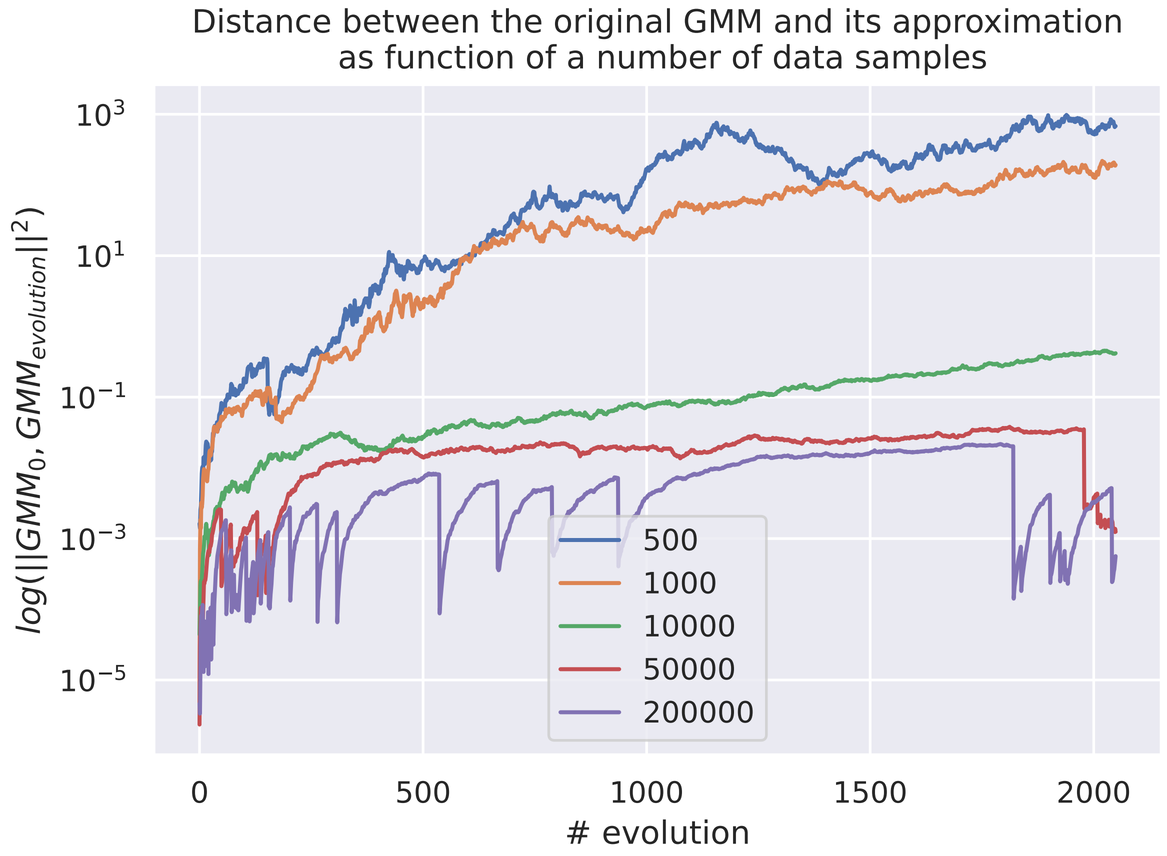
Distance between the original GMM and its approximation
as function of a number of data samples
$log(||GMM_0, GMM_{evolution}||^2)$
10^3
10^1
10^{-1}
10^{-3}
10^{-5}
0
500
1000
1500
2000
evolution
500
1000
10000
50000
200000
