# Supplementary material for: AI models collapse when trained on recursively generated data
Source: Nature. 2024 Jul 24;631(8022):755–9. doi: 10.1038/s41586-024-07566-y (PMC11269175; doi:10.1038/s41586-024-07566-y)

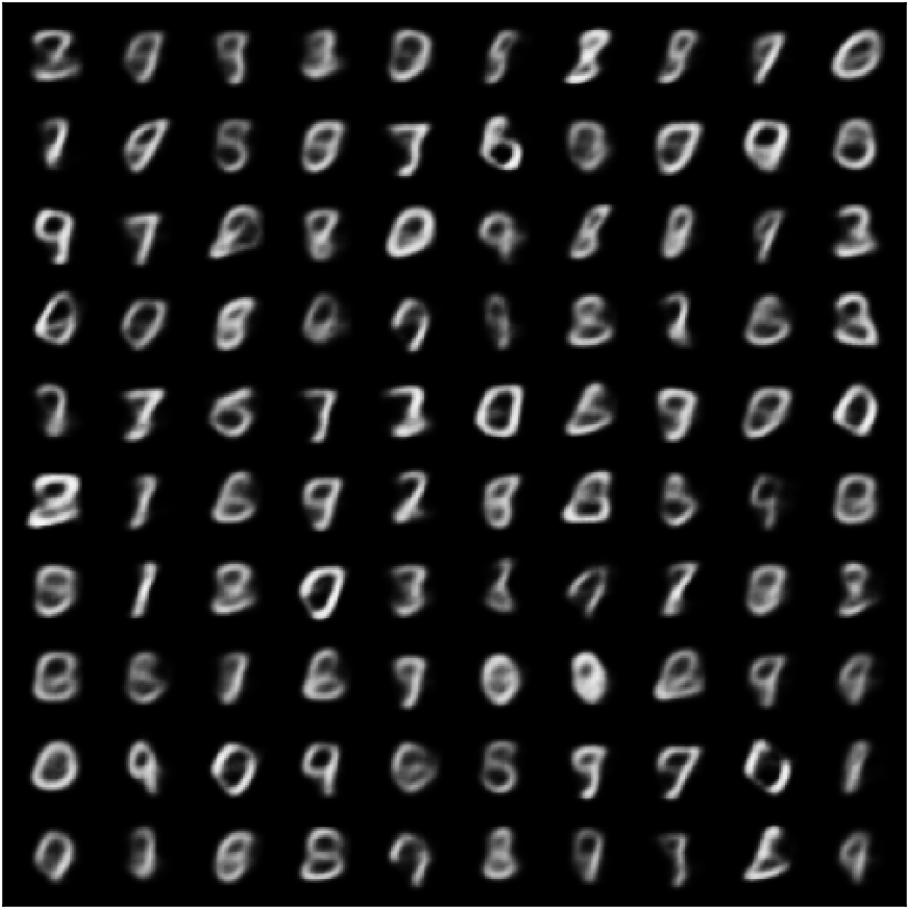

Supplement: Supplementary file 2 — Supplementary Data [file 41586_2024_7566_MOESM2_ESM.zip › images/vae/vae_gen5.pdf]
