# Supplementary figures and images for: AI models collapse when trained on recursively generated data
Source: Nature. 2024 Jul 24;631(8022):755–9. doi: 10.1038/s41586-024-07566-y (PMC11269175; doi:10.1038/s41586-024-07566-y)

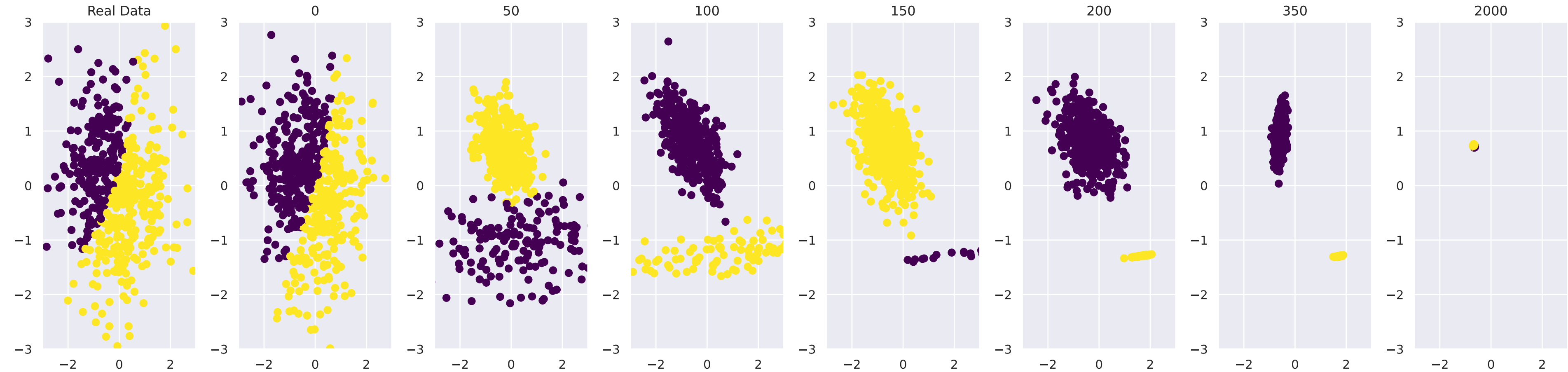

Supplement: Supplementary file 2 — Supplementary Data [file 41586_2024_7566_MOESM2_ESM.zip › images/GMM/gmm_example.pdf]

$\hat{\sigma}$  estimation of a  $\mathcal{N}(\mu = 0, \sigma = 1)$

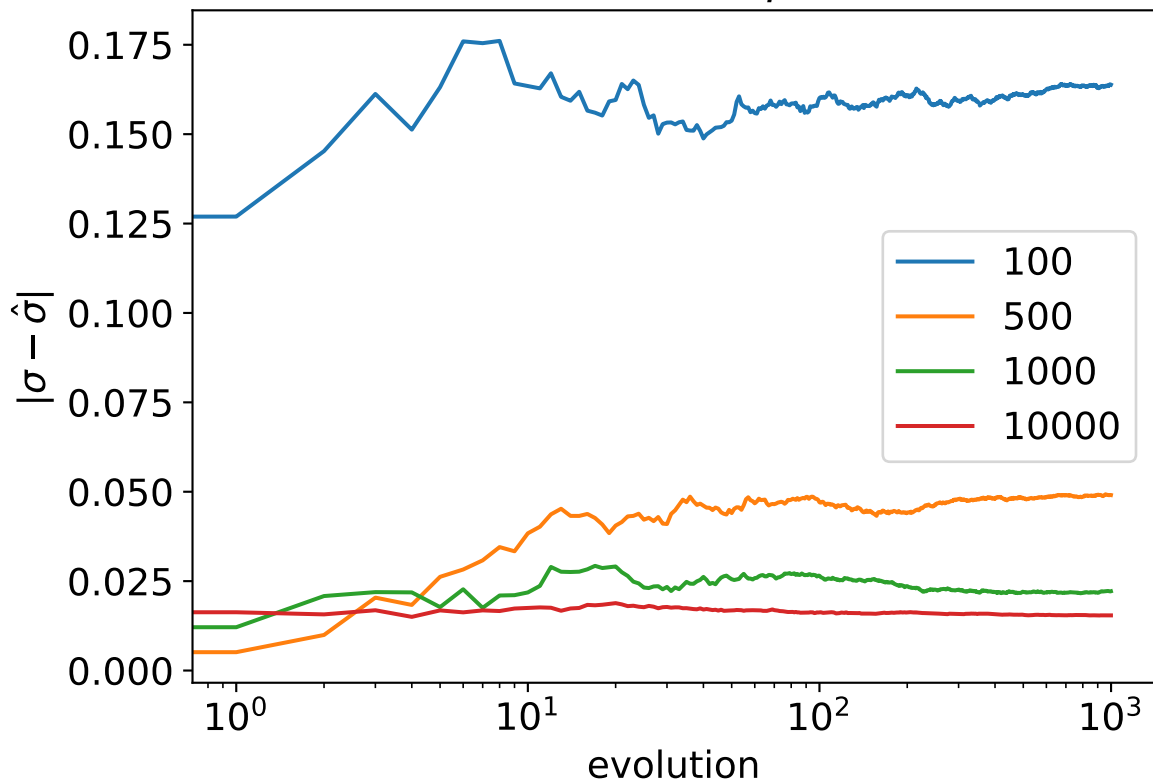

Supplement: Supplementary file 2 — Supplementary Data [file 41586_2024_7566_MOESM2_ESM.zip › images/singlegaus/alldat_single_normal_approx_sigma.pdf]

$\hat{\mu}$  estimation of a  $\mathcal{N}(\mu = 0, \sigma = 1)$

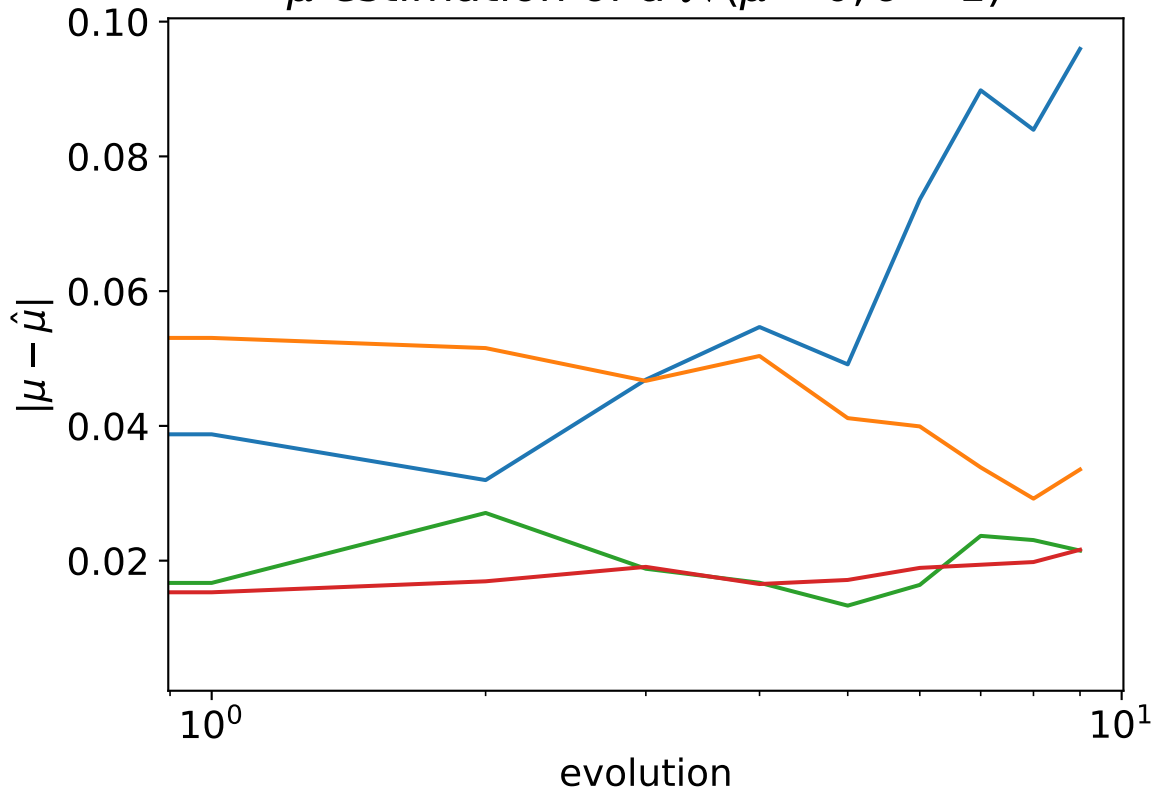

Supplement: Supplementary file 2 — Supplementary Data [file 41586_2024_7566_MOESM2_ESM.zip › images/singlegaus/alldat_single_normal_approx_mu-1.pdf]

$\hat{\mu}$  estimation of a  $\mathcal{N}(\mu = 0, \sigma = 1)$

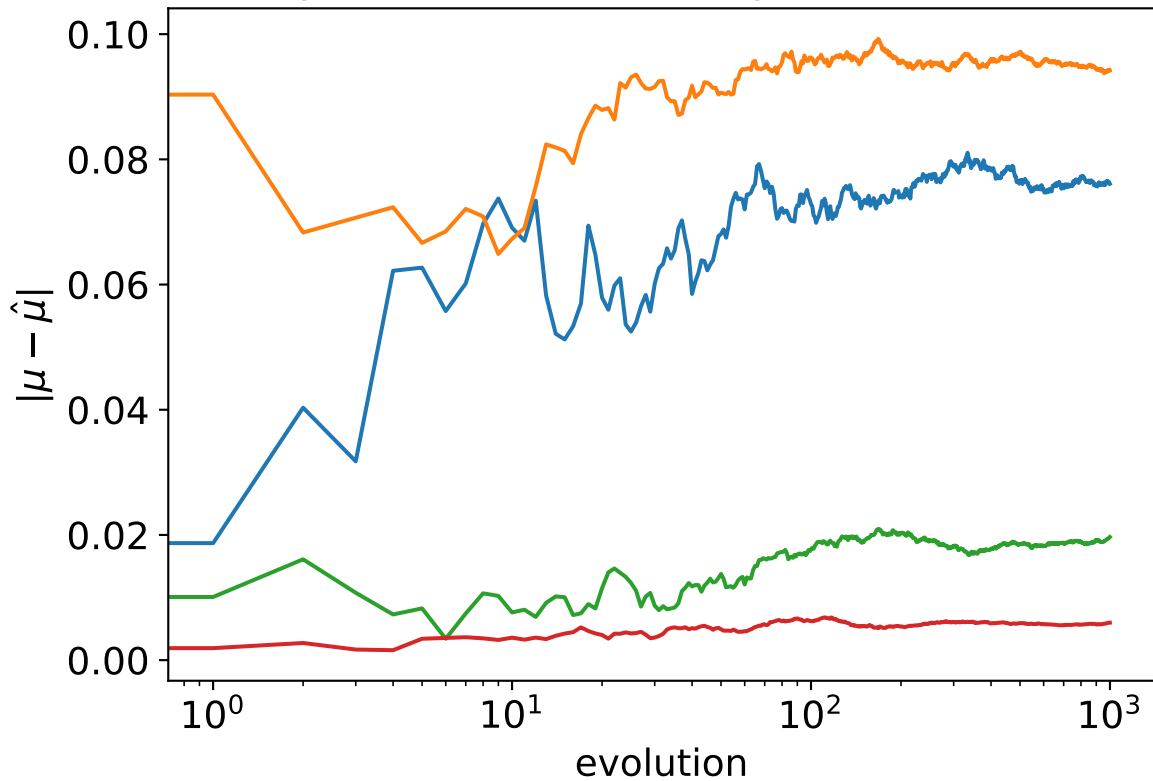

Supplement: Supplementary file 2 — Supplementary Data [file 41586_2024_7566_MOESM2_ESM.zip › images/singlegaus/alldat_single_normal_approx_mu.pdf]

$\hat{\mu}$  estimation of a  $\mathcal{N}(\mu = 0, \sigma = 1)$

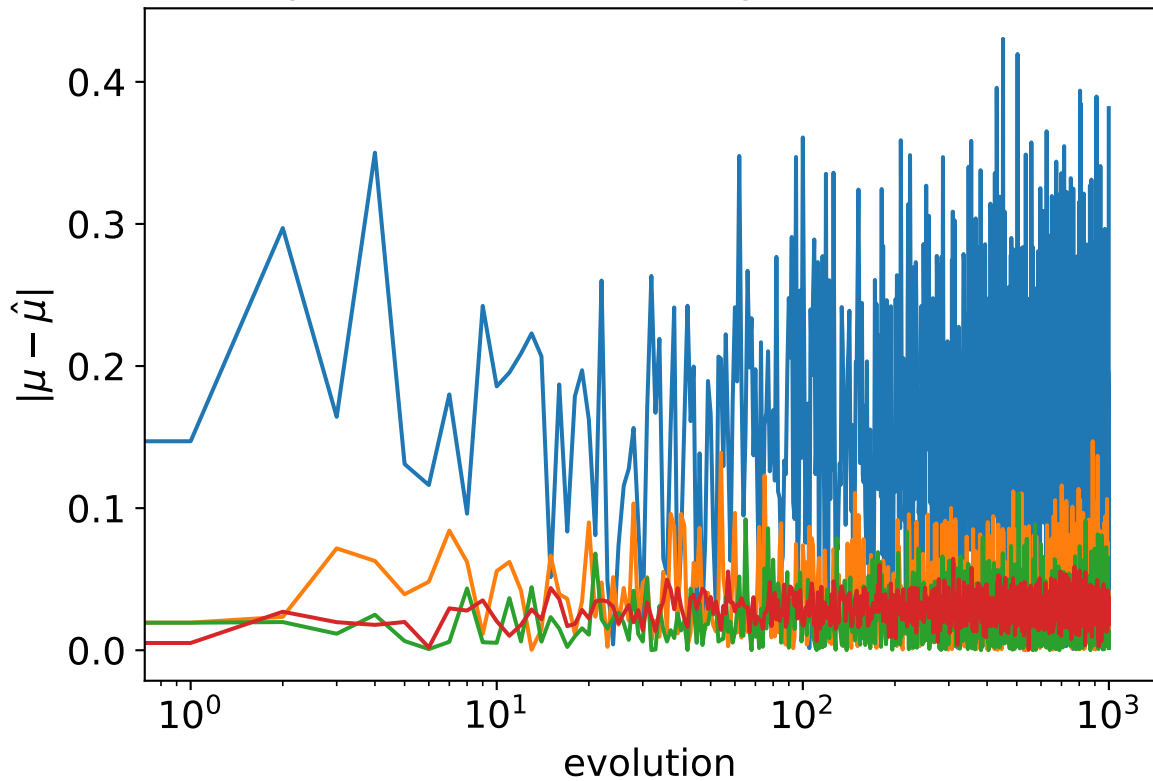

Supplement: Supplementary file 2 — Supplementary Data [file 41586_2024_7566_MOESM2_ESM.zip › images/singlegaus/alldat_samp_single_normal_approx_mu.pdf]

$\hat{\sigma}$  estimation of a  $\mathcal{N}(\mu = 0, \sigma = 1)$

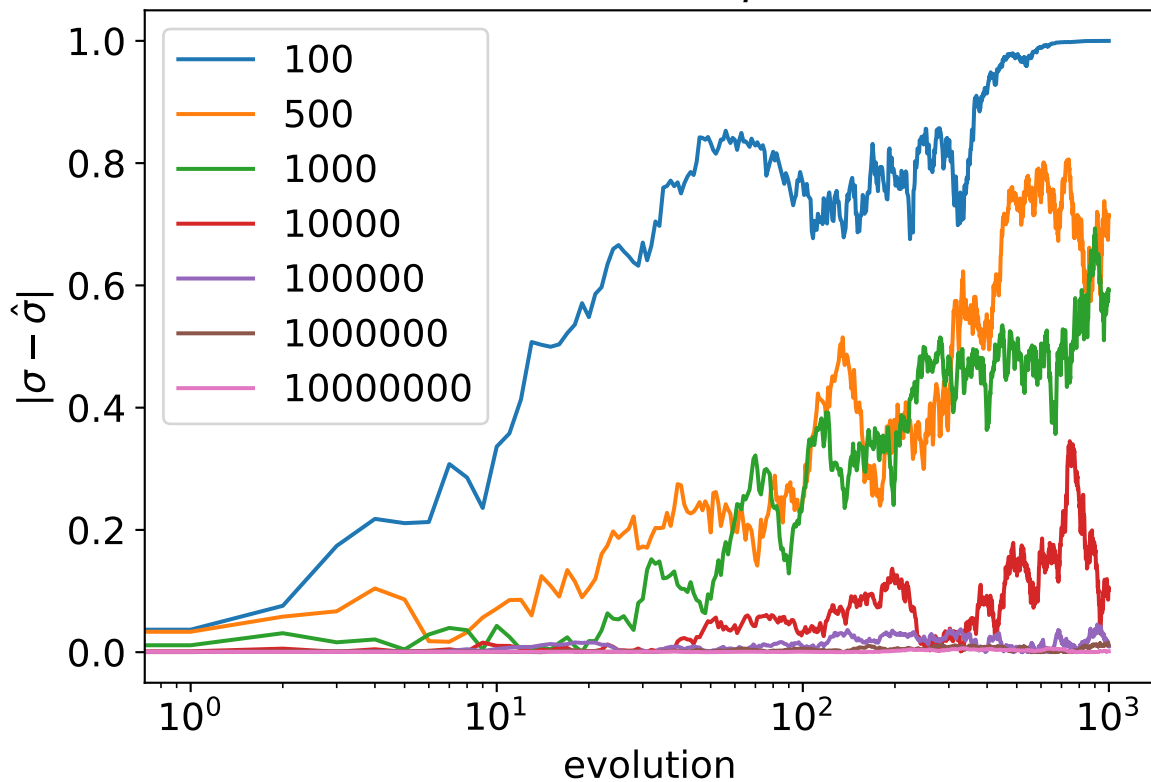

Supplement: Supplementary file 2 — Supplementary Data [file 41586_2024_7566_MOESM2_ESM.zip › images/singlegaus/single_normal_approx_sigma.pdf]

Real distribution 1

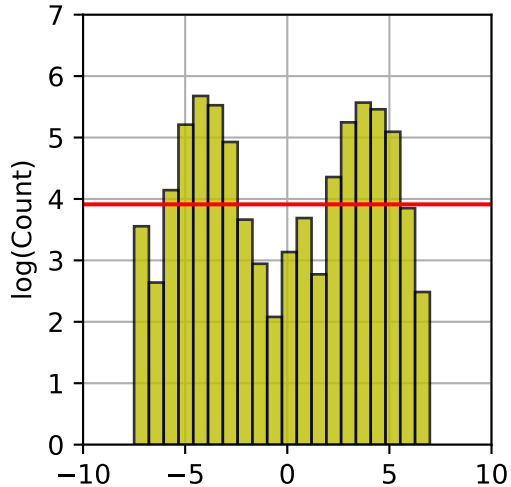

Real distribution 2

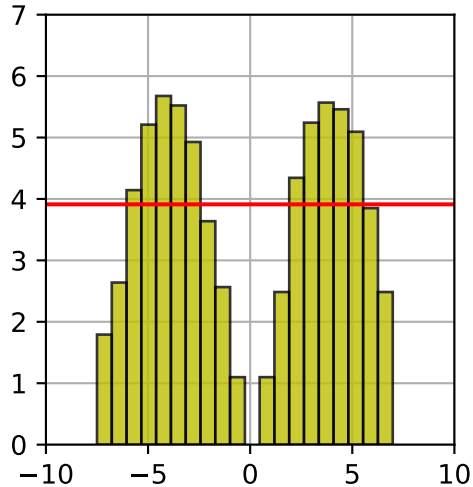

Resampled 1 and 2

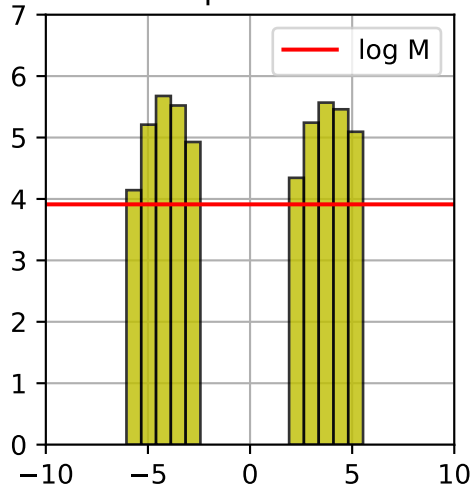

Supplement: Supplementary file 2 — Supplementary Data [file 41586_2024_7566_MOESM2_ESM.zip › images/singlegaus/hist_ex.pdf]

$\hat{\sigma}$  estimation of a  $\mathcal{N}(\mu = 0, \sigma = 1)$

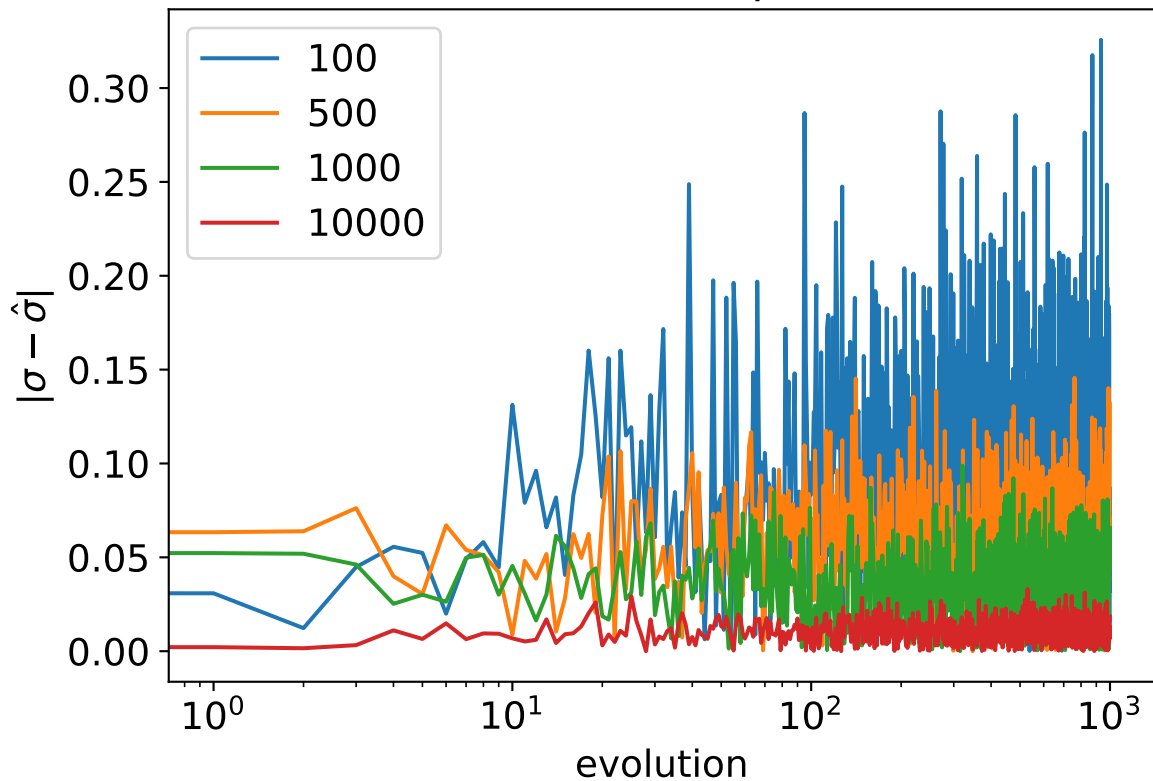

Supplement: Supplementary file 2 — Supplementary Data [file 41586_2024_7566_MOESM2_ESM.zip › images/singlegaus/alldat_samp_single_normal_approx_sigma.pdf]

$\hat{\mu}$  estimation of a  $\mathcal{N}(\mu = 0, \sigma = 1)$

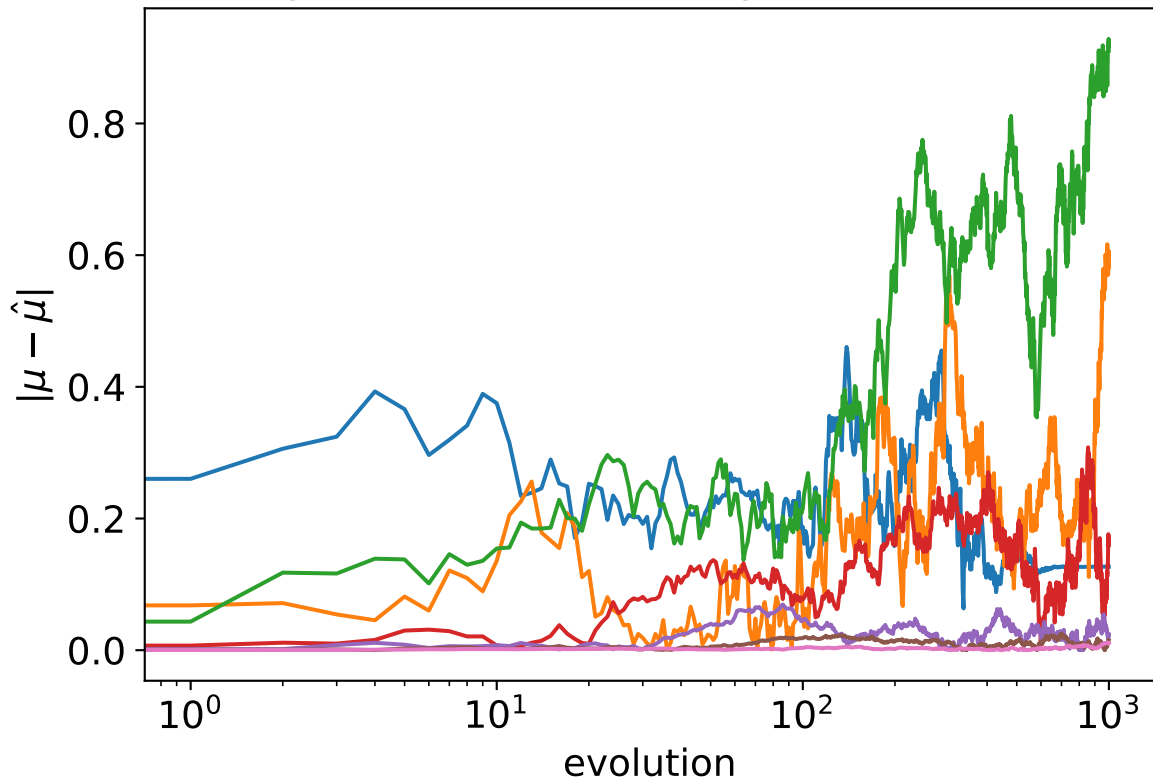

Supplement: Supplementary file 2 — Supplementary Data [file 41586_2024_7566_MOESM2_ESM.zip › images/singlegaus/single_normal_approx_mu.pdf]

$\hat{\sigma}$  estimation of a  $\mathcal{N}(\mu = 0, \sigma = 1)$

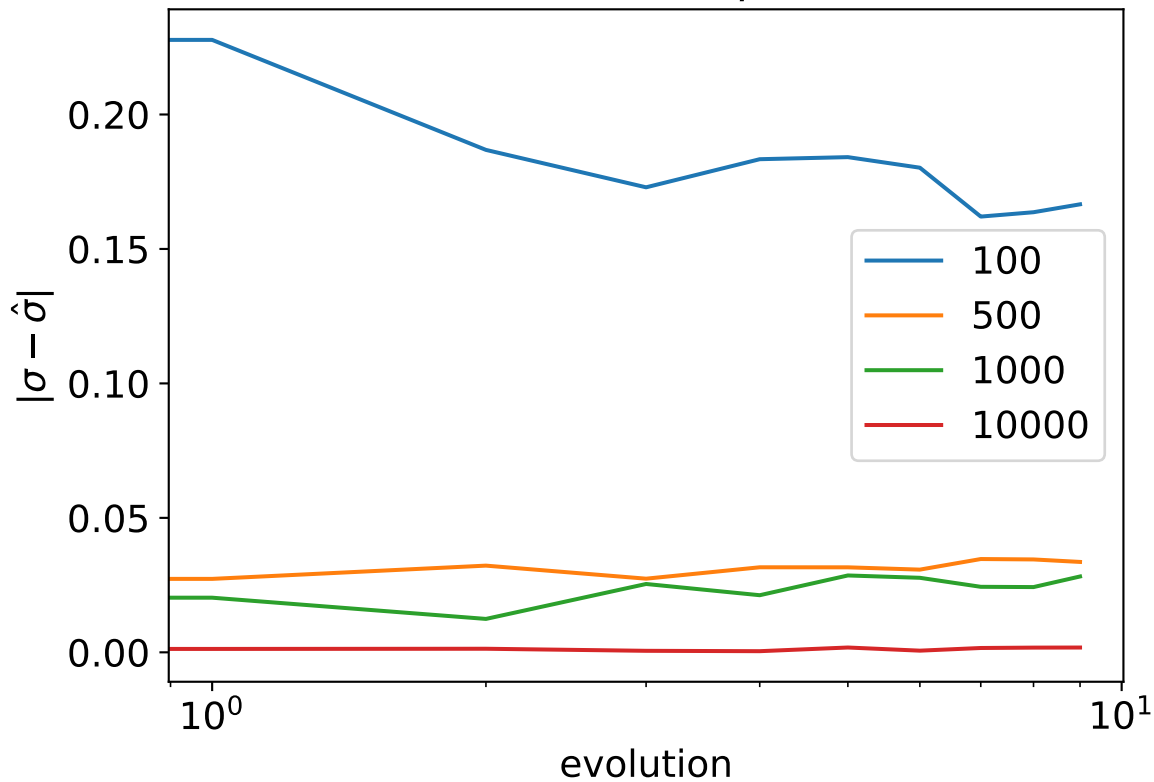

Supplement: Supplementary file 2 — Supplementary Data [file 41586_2024_7566_MOESM2_ESM.zip › images/singlegaus/alldat_single_normal_approx_sigma-1.pdf]

$\hat{\mu}$  estimation of a  $\mathcal{N}(\mu = 0, \sigma = 1)$

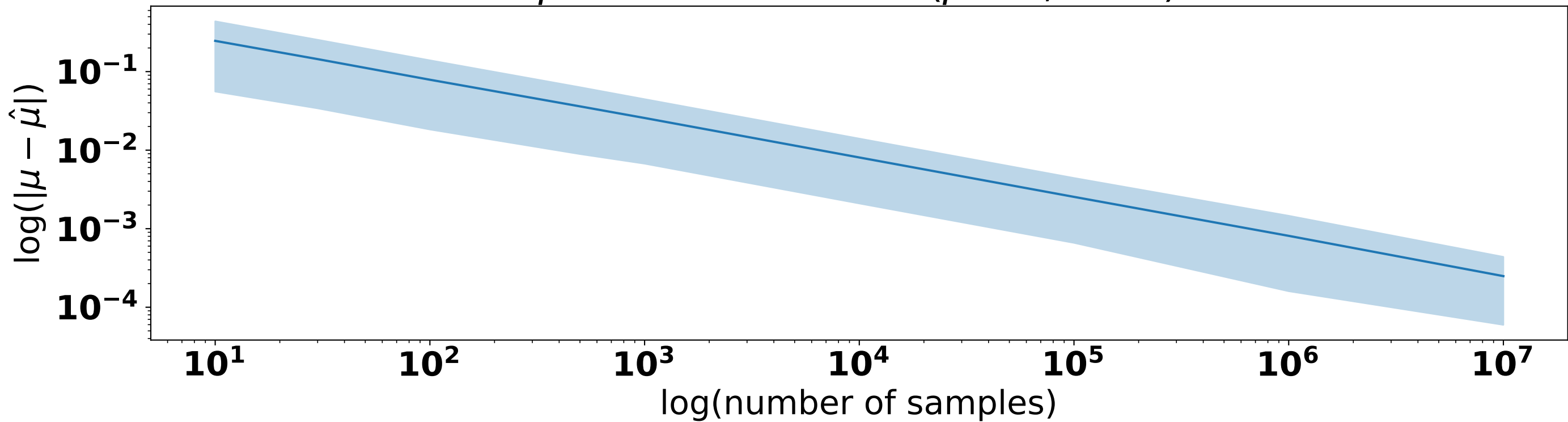

Supplement: Supplementary file 2 — Supplementary Data [file 41586_2024_7566_MOESM2_ESM.zip › images/infographics/single_normal_approx.pdf]

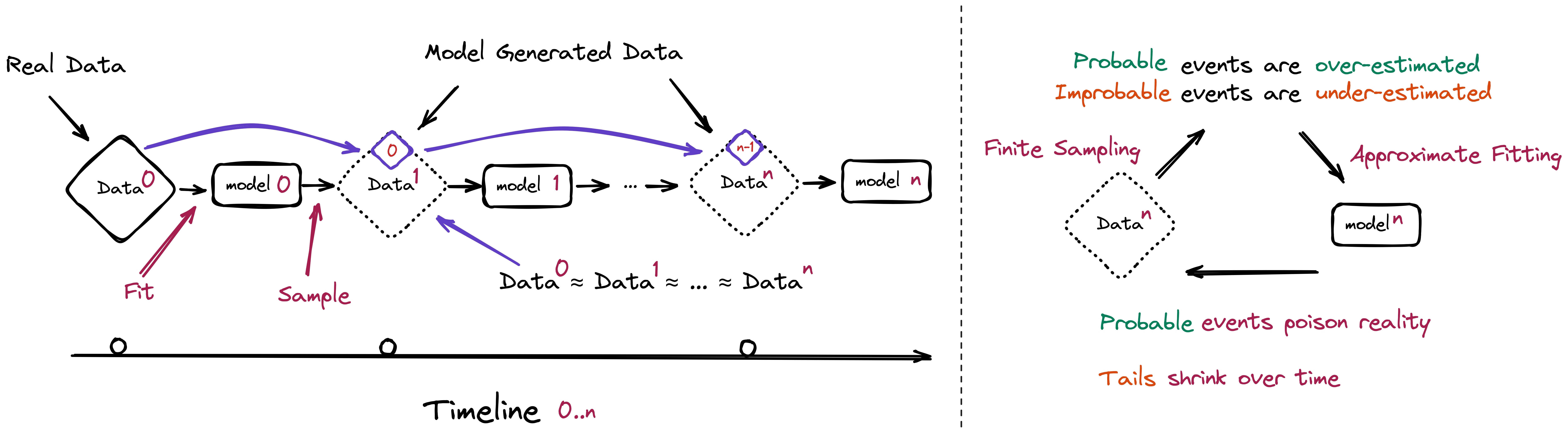

Supplement: Supplementary file 2 — Supplementary Data [file 41586_2024_7566_MOESM2_ESM.zip › images/infographics/combined_timeline.png]

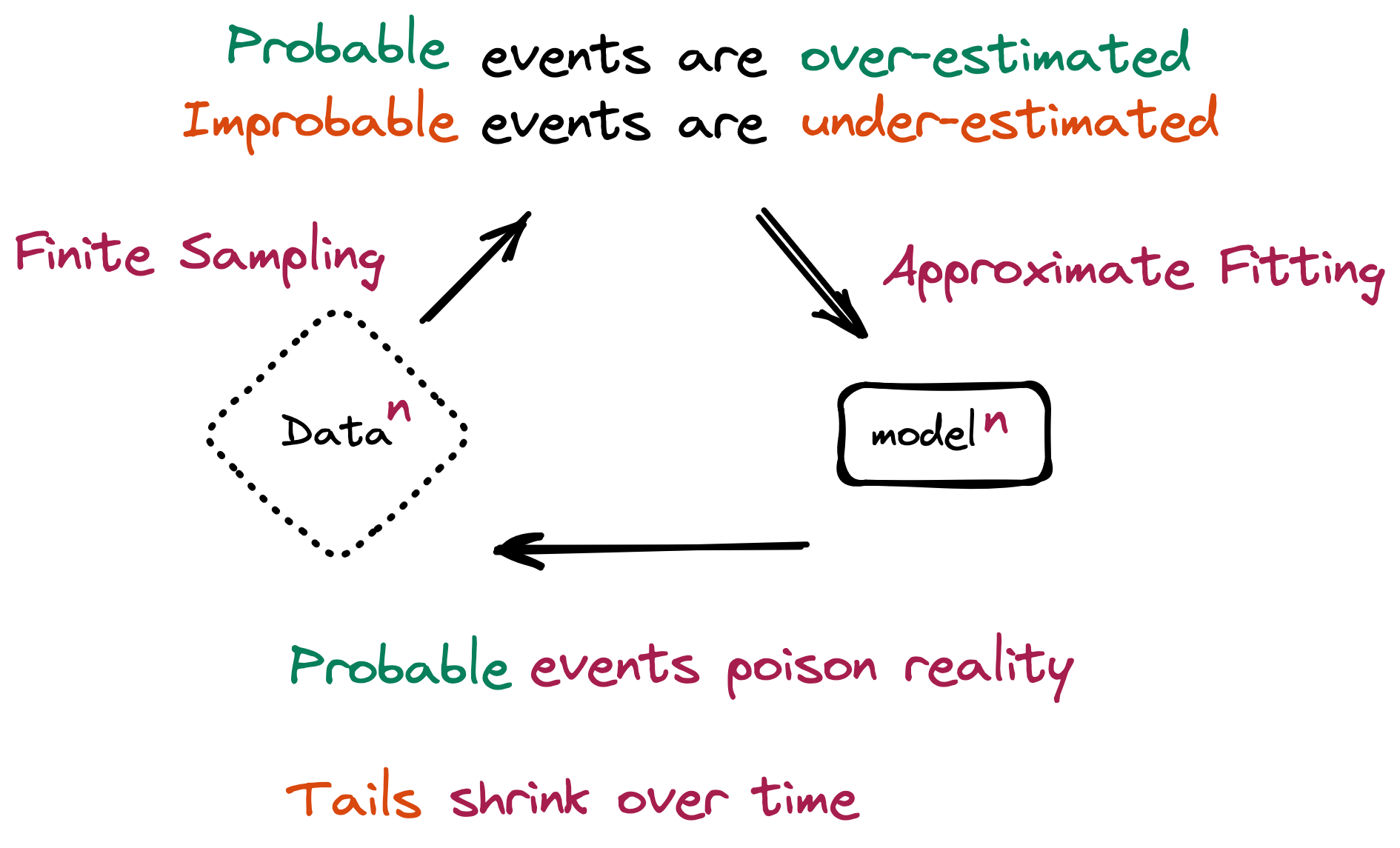

Supplement: Supplementary file 2 — Supplementary Data [file 41586_2024_7566_MOESM2_ESM.zip › images/infographics/timeline_2.png]

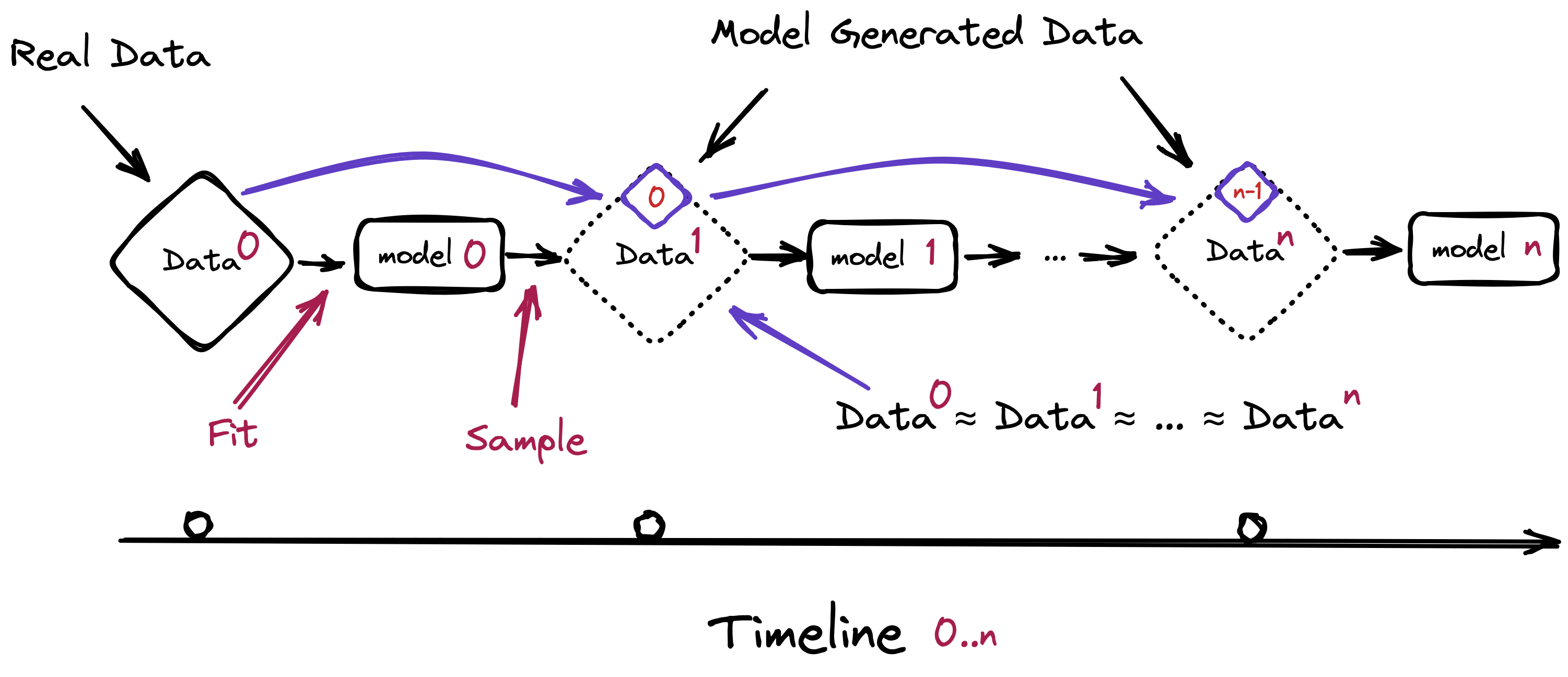

Supplement: Supplementary file 2 — Supplementary Data [file 41586_2024_7566_MOESM2_ESM.zip › images/infographics/timeline.png]

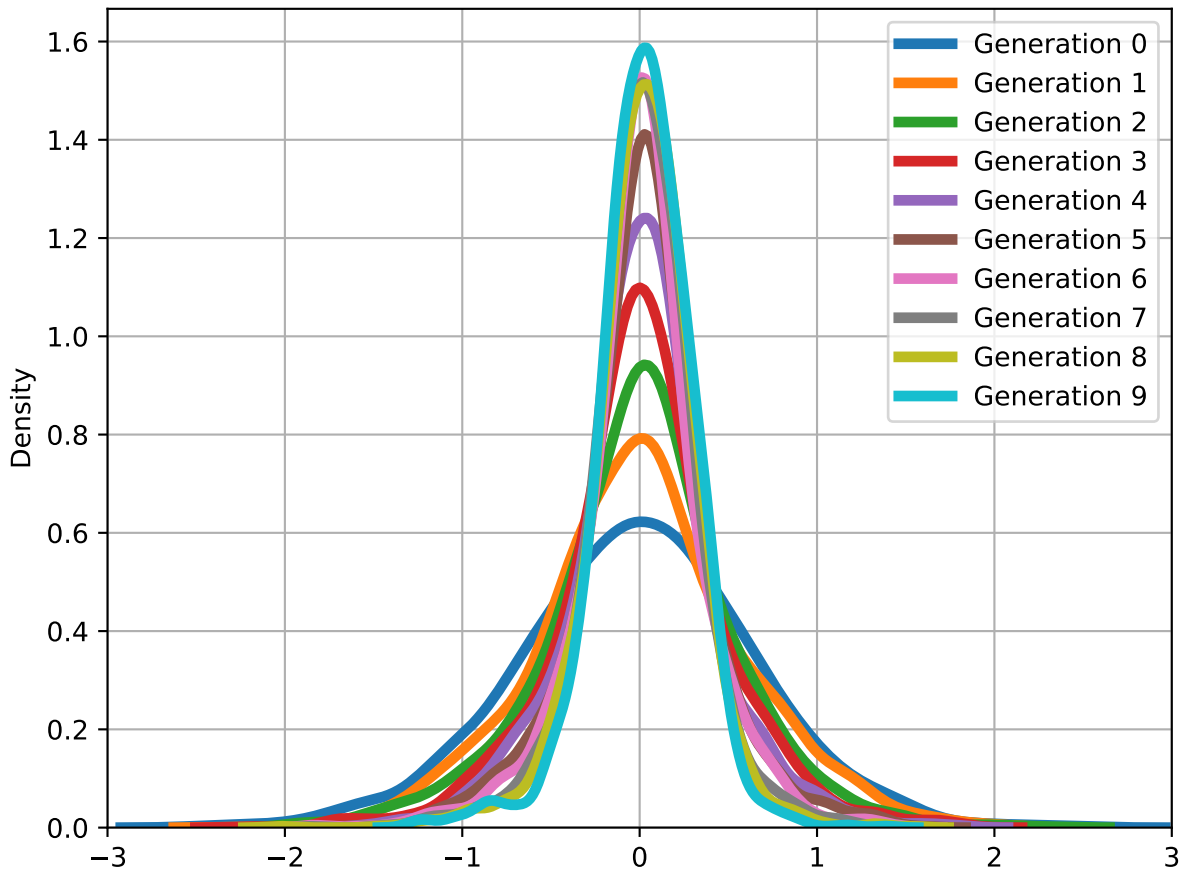

Supplement: Supplementary file 2 — Supplementary Data [file 41586_2024_7566_MOESM2_ESM.zip › images/vae/iters.pdf]

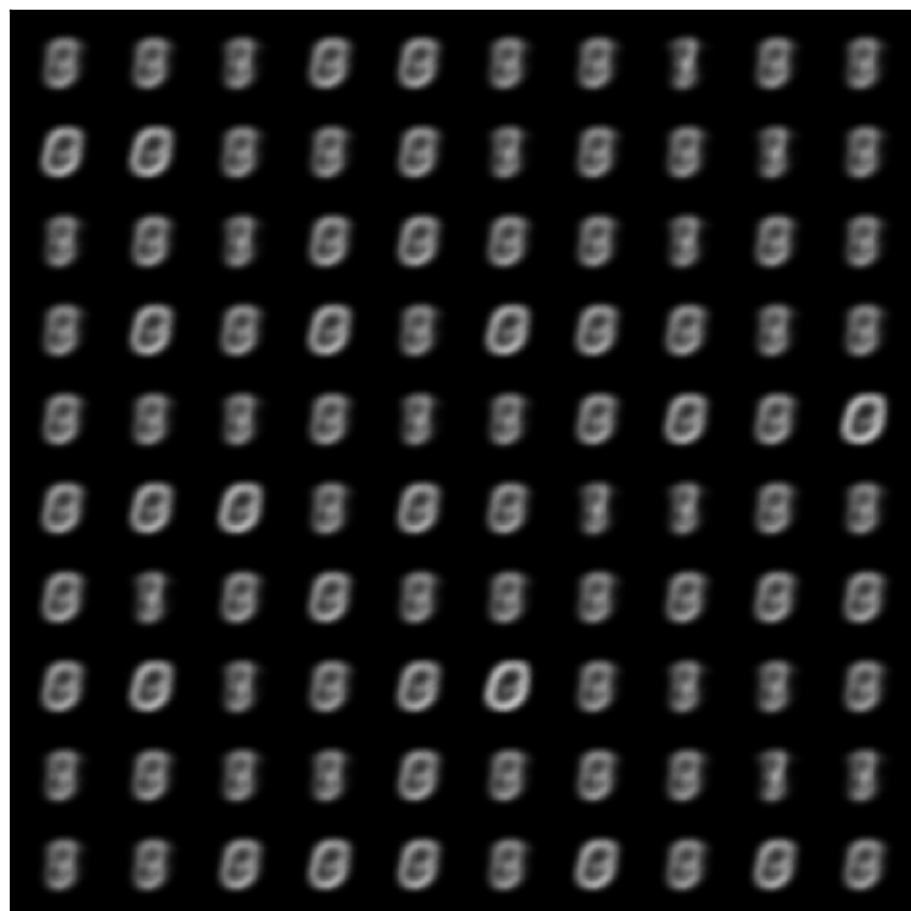

Supplement: Supplementary file 2 — Supplementary Data [file 41586_2024_7566_MOESM2_ESM.zip › images/vae/vae_epoch20.pdf]

# Perplexity of generated datapoints evaluated by model trained with real wikitext2

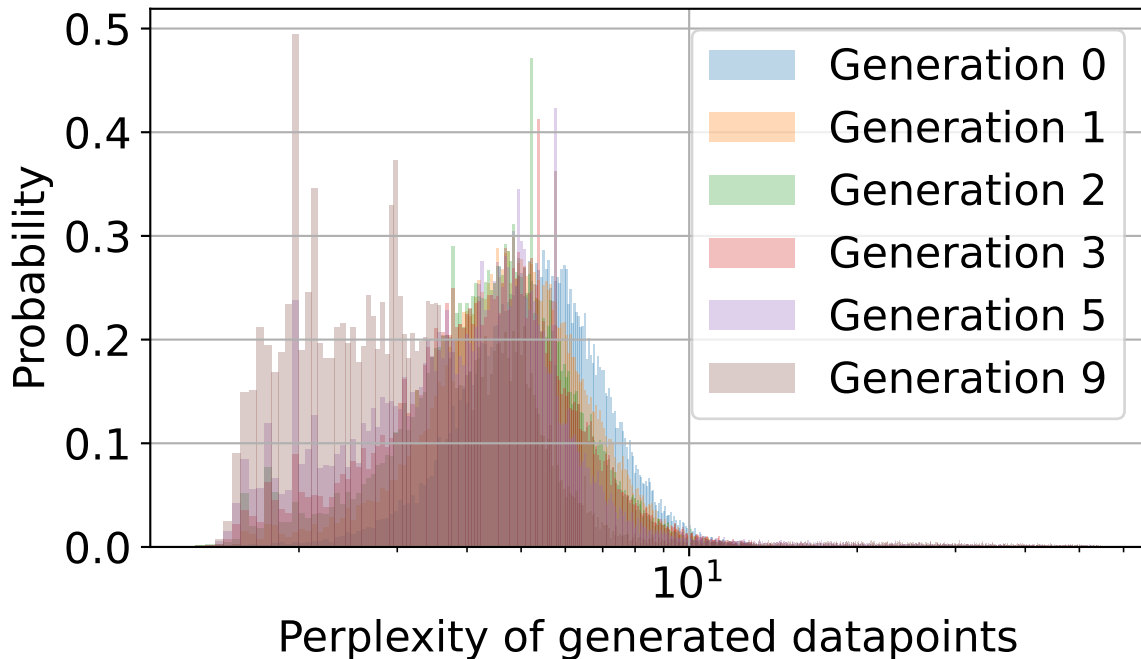

Supplement: Supplementary file 2 — Supplementary Data [file 41586_2024_7566_MOESM2_ESM.zip › images/lang/norepeat/hist.pdf]

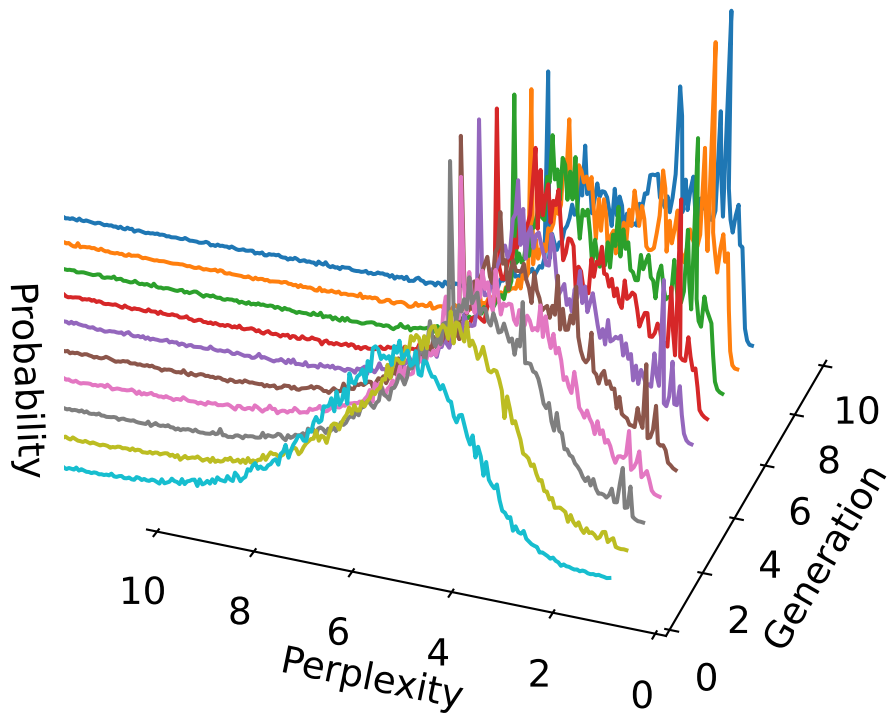

Supplement: Supplementary file 2 — Supplementary Data [file 41586_2024_7566_MOESM2_ESM.zip › images/lang/norepeat/3dhist.pdf]

# Perplexity of generated datapoints evaluated by model trained with real wikitext2

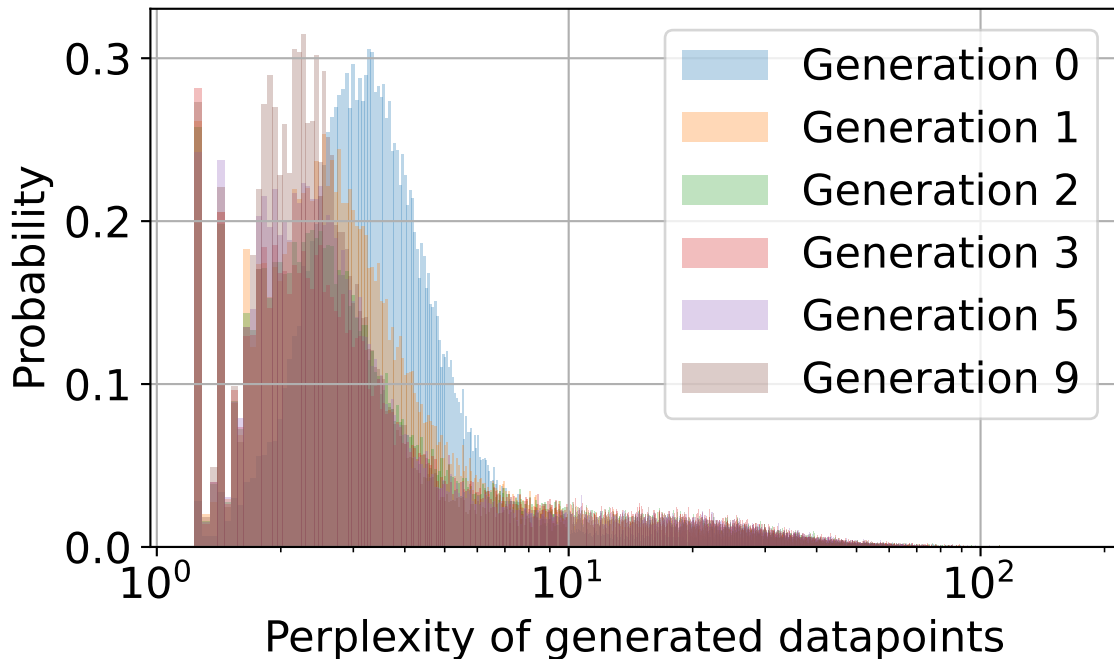

Supplement: Supplementary file 2 — Supplementary Data [file 41586_2024_7566_MOESM2_ESM.zip › images/lang/10saved/hist.pdf]

# Real wikitext2 test dataset

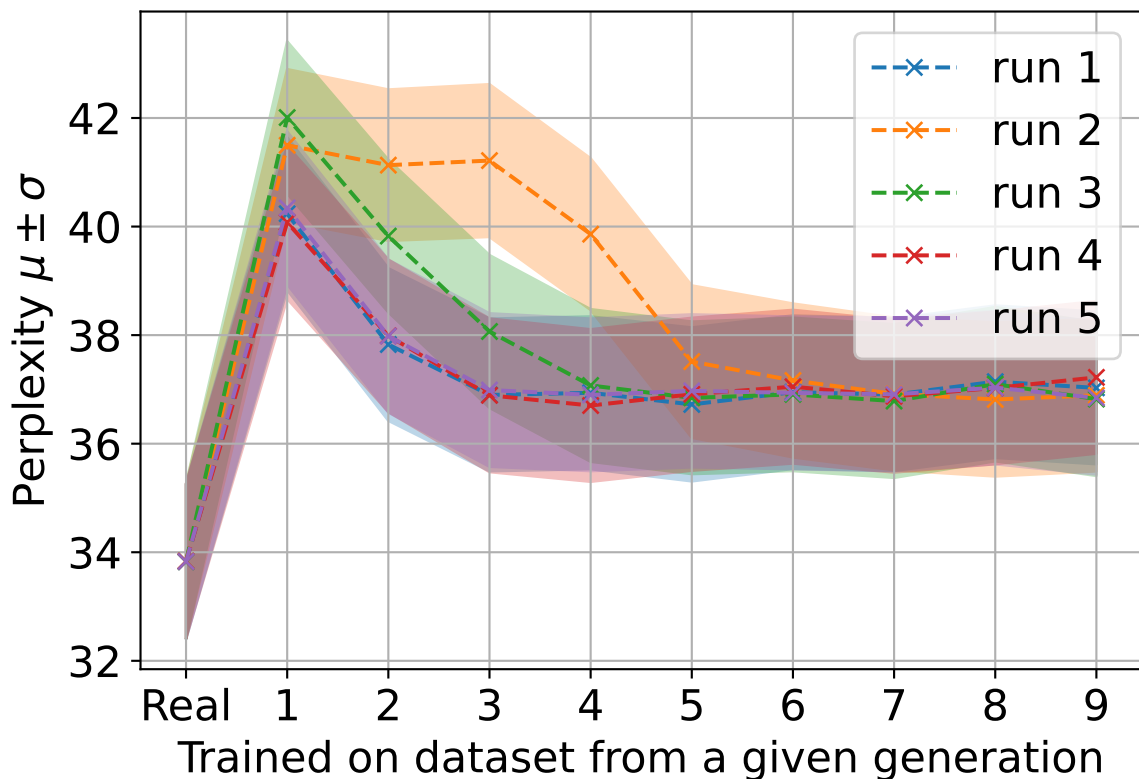

Supplement: Supplementary file 2 — Supplementary Data [file 41586_2024_7566_MOESM2_ESM.zip › images/lang/10saved/baseperf.pdf]

# OPT on Wikitext2 sampled data

## Performance of model from generation 9

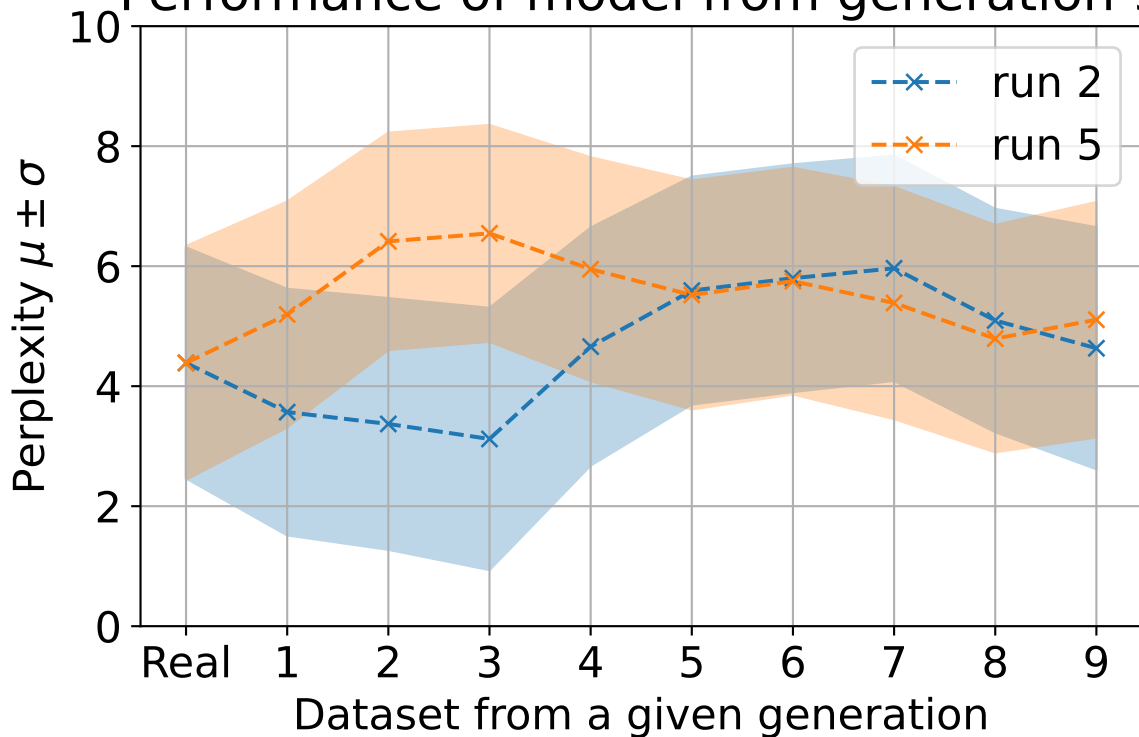

Supplement: Supplementary file 2 — Supplementary Data [file 41586_2024_7566_MOESM2_ESM.zip › images/lang/10saved/frm_9.pdf]

# OPT on Wikitext2 sampled data

## Performance of model from generation 4

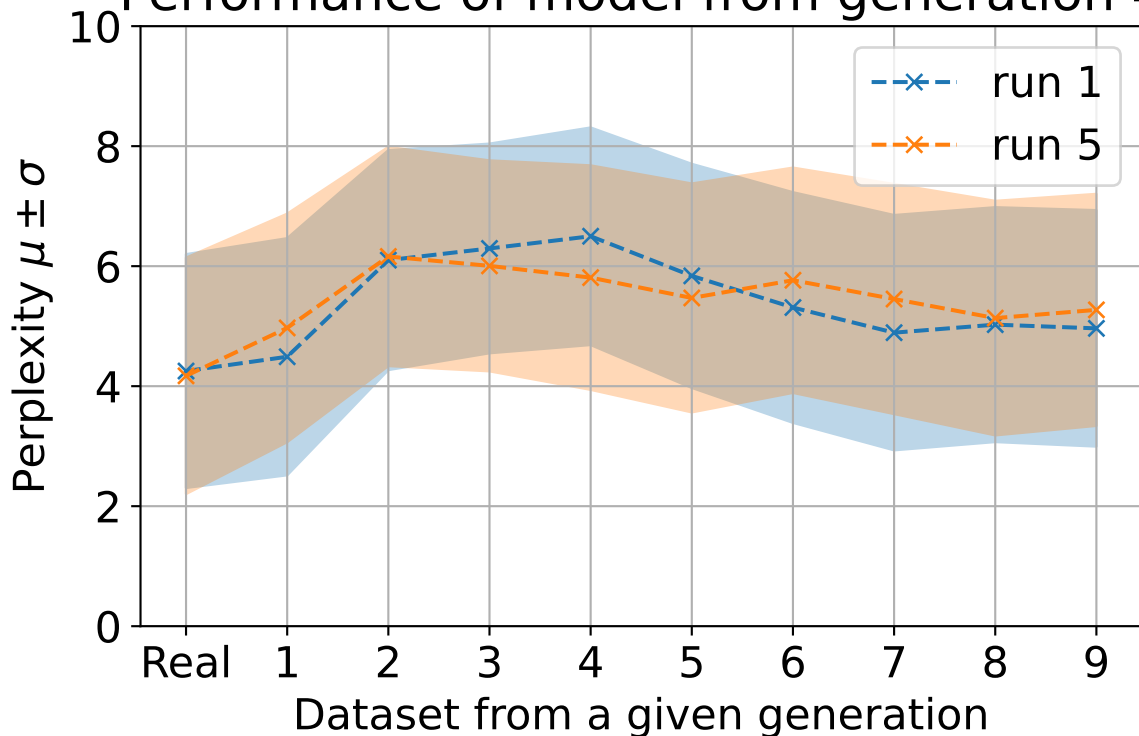

Supplement: Supplementary file 2 — Supplementary Data [file 41586_2024_7566_MOESM2_ESM.zip › images/lang/10saved/frm_4.pdf]

# OPT on Wikitext2 sampled data

## Performance of model from generation 1

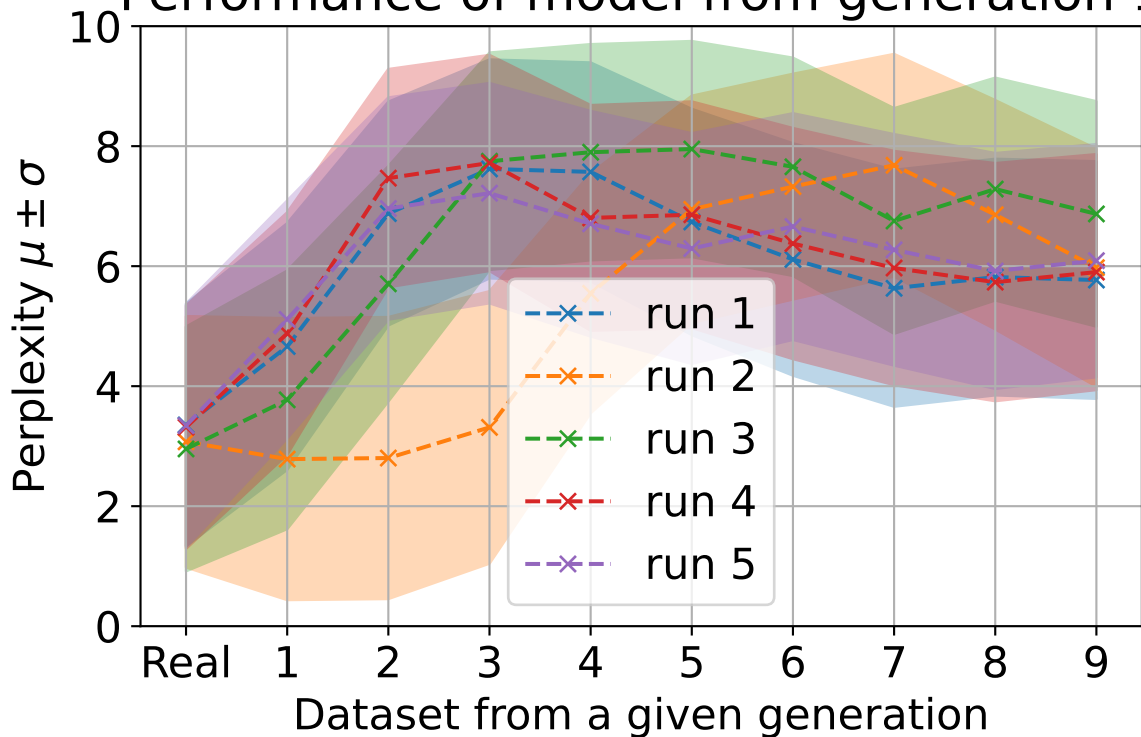

Supplement: Supplementary file 2 — Supplementary Data [file 41586_2024_7566_MOESM2_ESM.zip › images/lang/10saved/frm_1.pdf]

# OPT on Wikitext2 sampled data

## Performance of model from generation 0

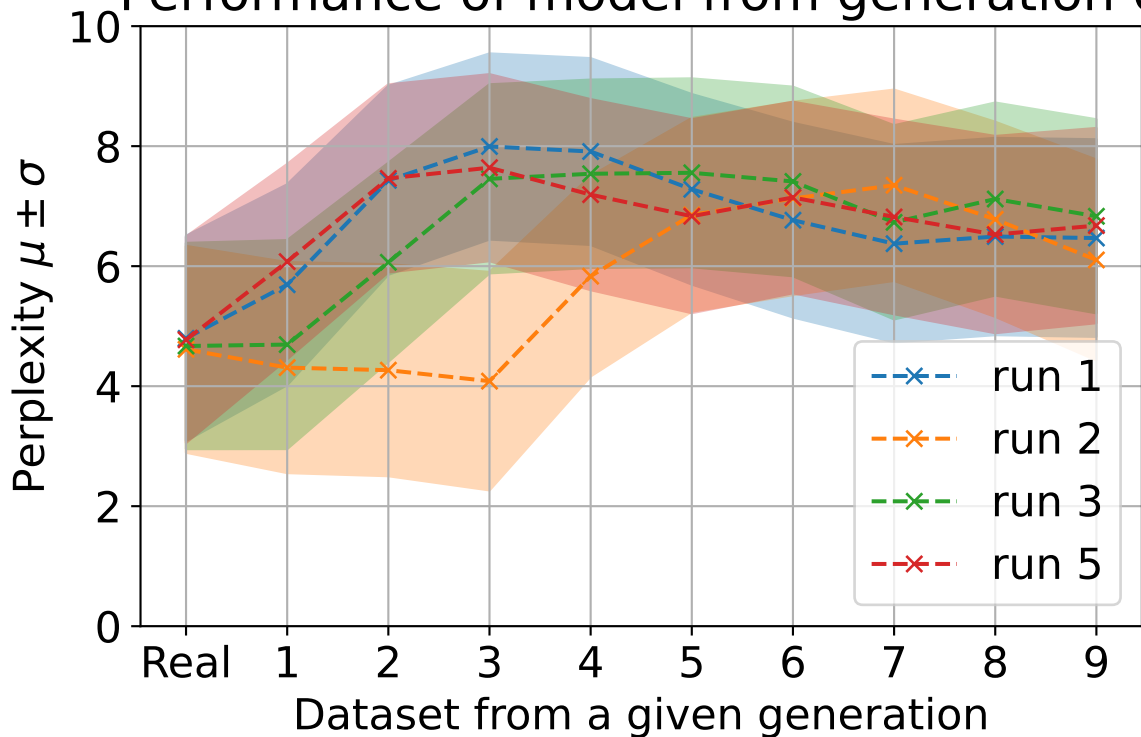

Supplement: Supplementary file 2 — Supplementary Data [file 41586_2024_7566_MOESM2_ESM.zip › images/lang/10saved/frm_0.pdf]

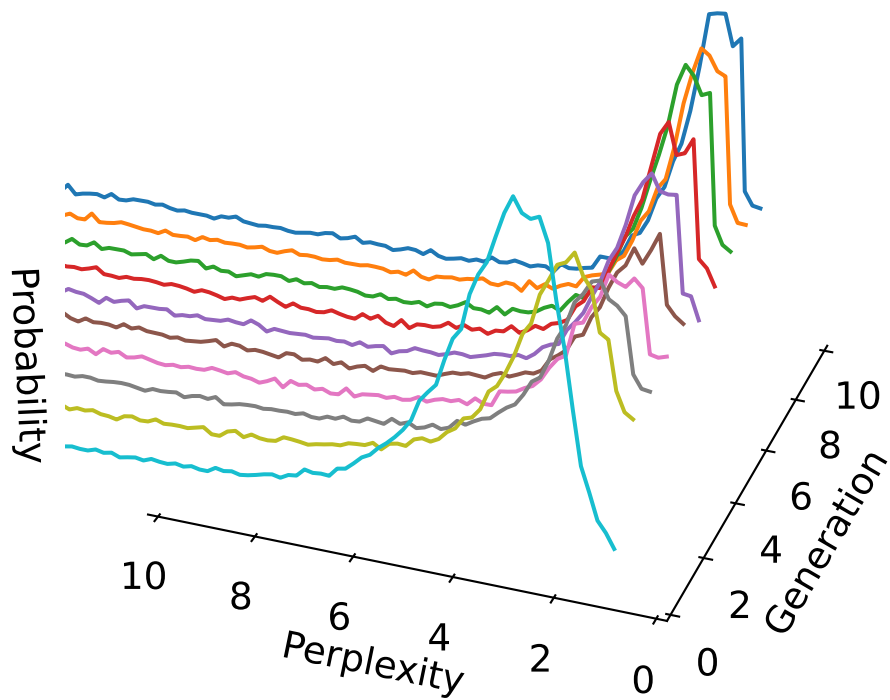

Supplement: Supplementary file 2 — Supplementary Data [file 41586_2024_7566_MOESM2_ESM.zip › images/lang/10saved/3dhist.pdf]

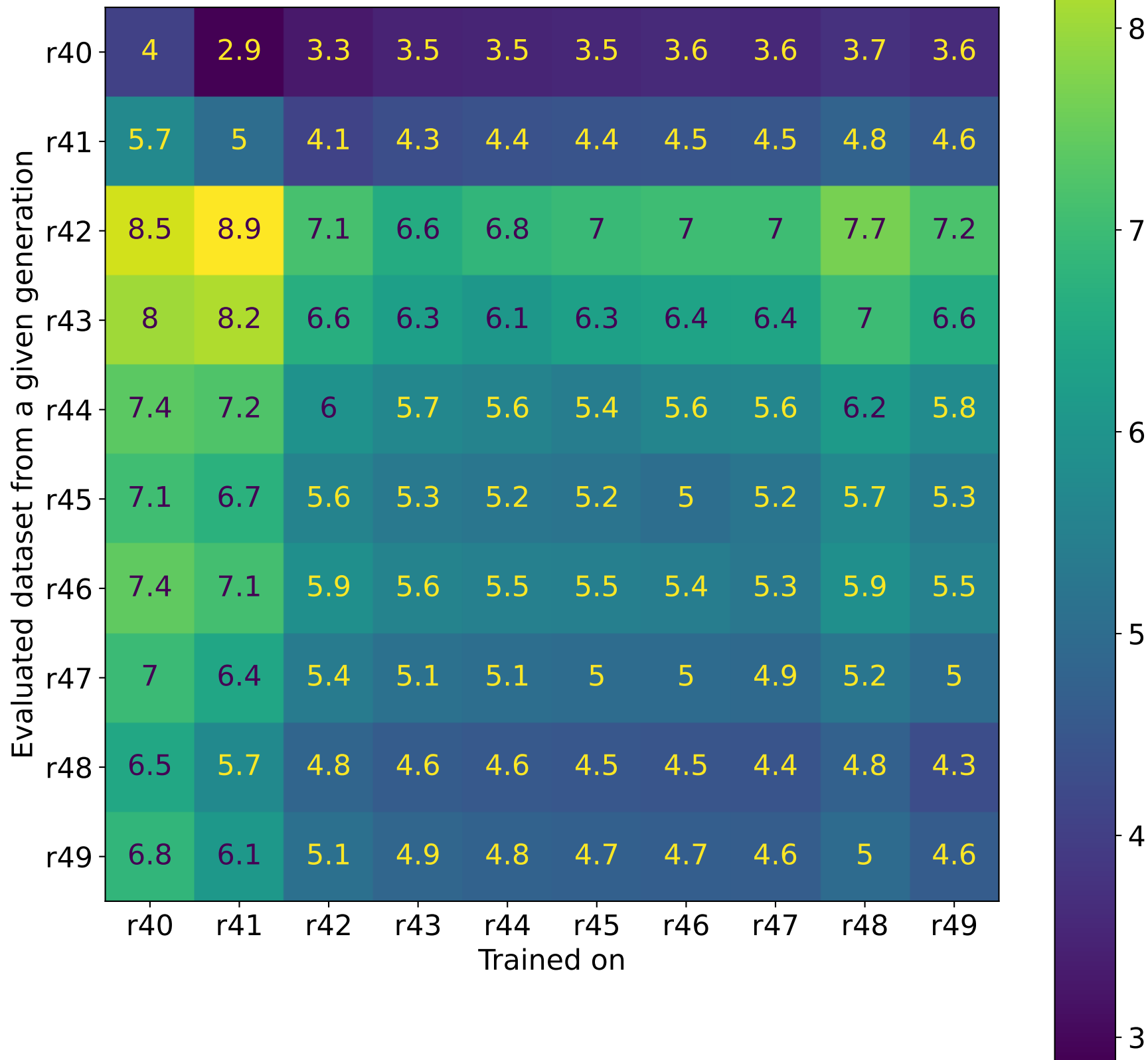

Supplement: Supplementary file 2 — Supplementary Data [file 41586_2024_7566_MOESM2_ESM.zip › images/lang/0saved/cm_4.pdf]

# Perplexity of generated datapoints evaluated by model trained with real wikitext2

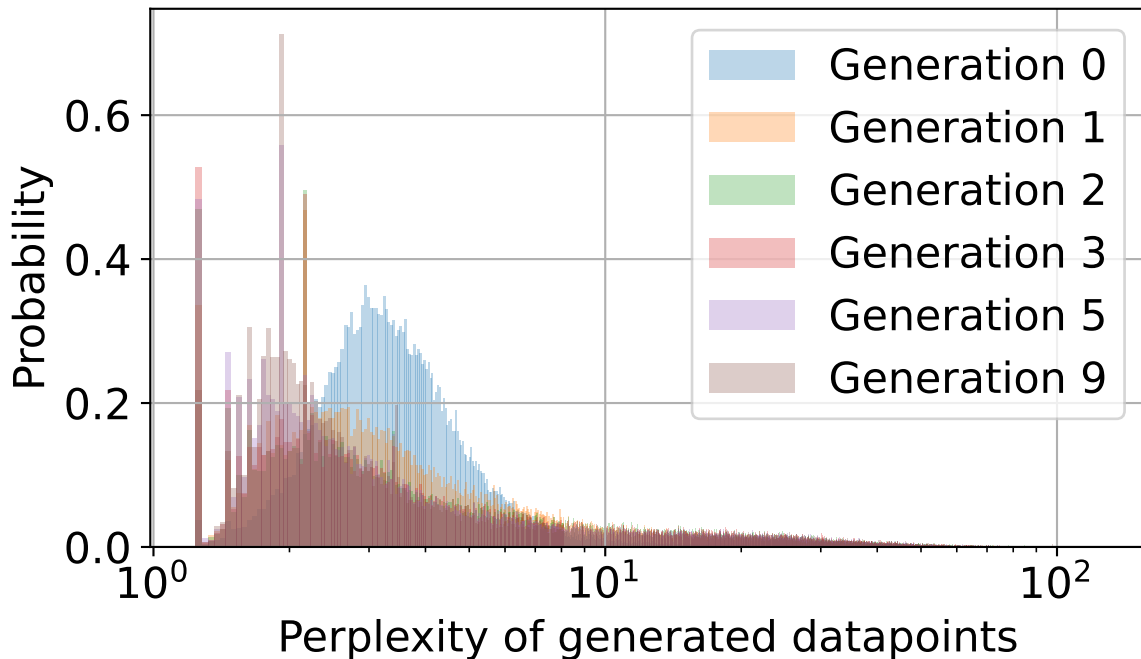

Supplement: Supplementary file 2 — Supplementary Data [file 41586_2024_7566_MOESM2_ESM.zip › images/lang/0saved/hist.pdf]

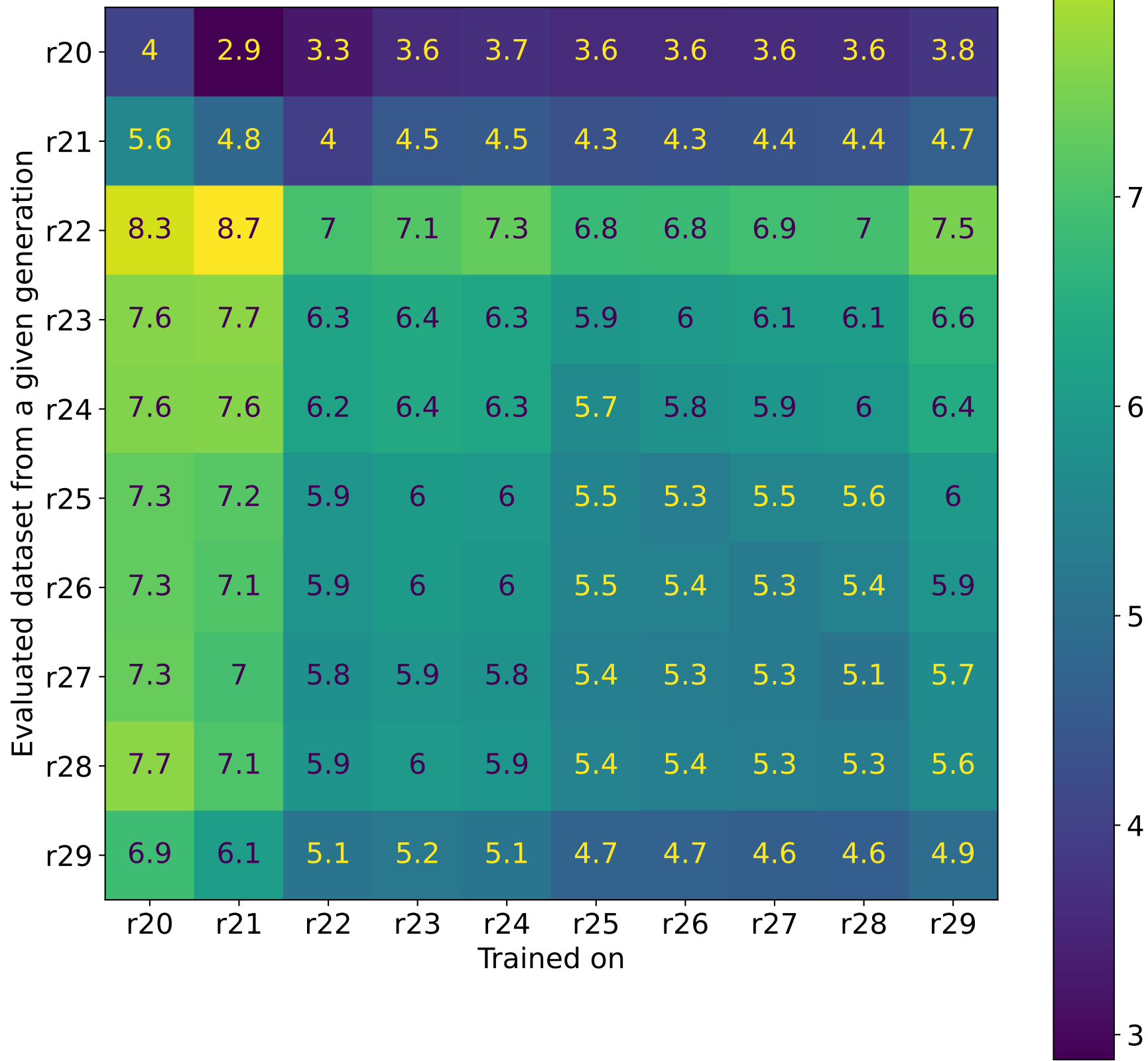

Supplement: Supplementary file 2 — Supplementary Data [file 41586_2024_7566_MOESM2_ESM.zip › images/lang/0saved/cm_2.pdf]

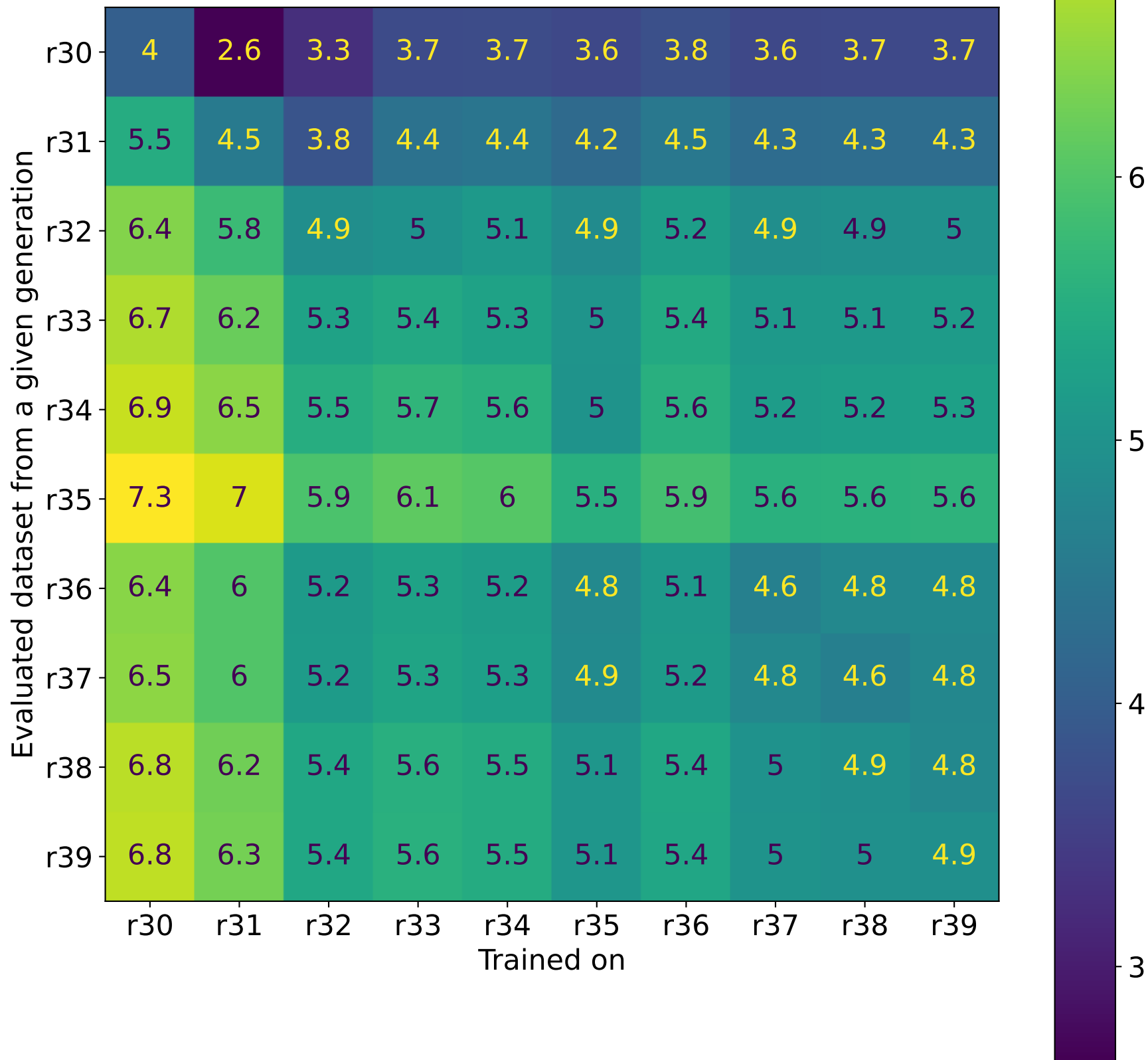

Supplement: Supplementary file 2 — Supplementary Data [file 41586_2024_7566_MOESM2_ESM.zip › images/lang/0saved/cm_3.pdf]

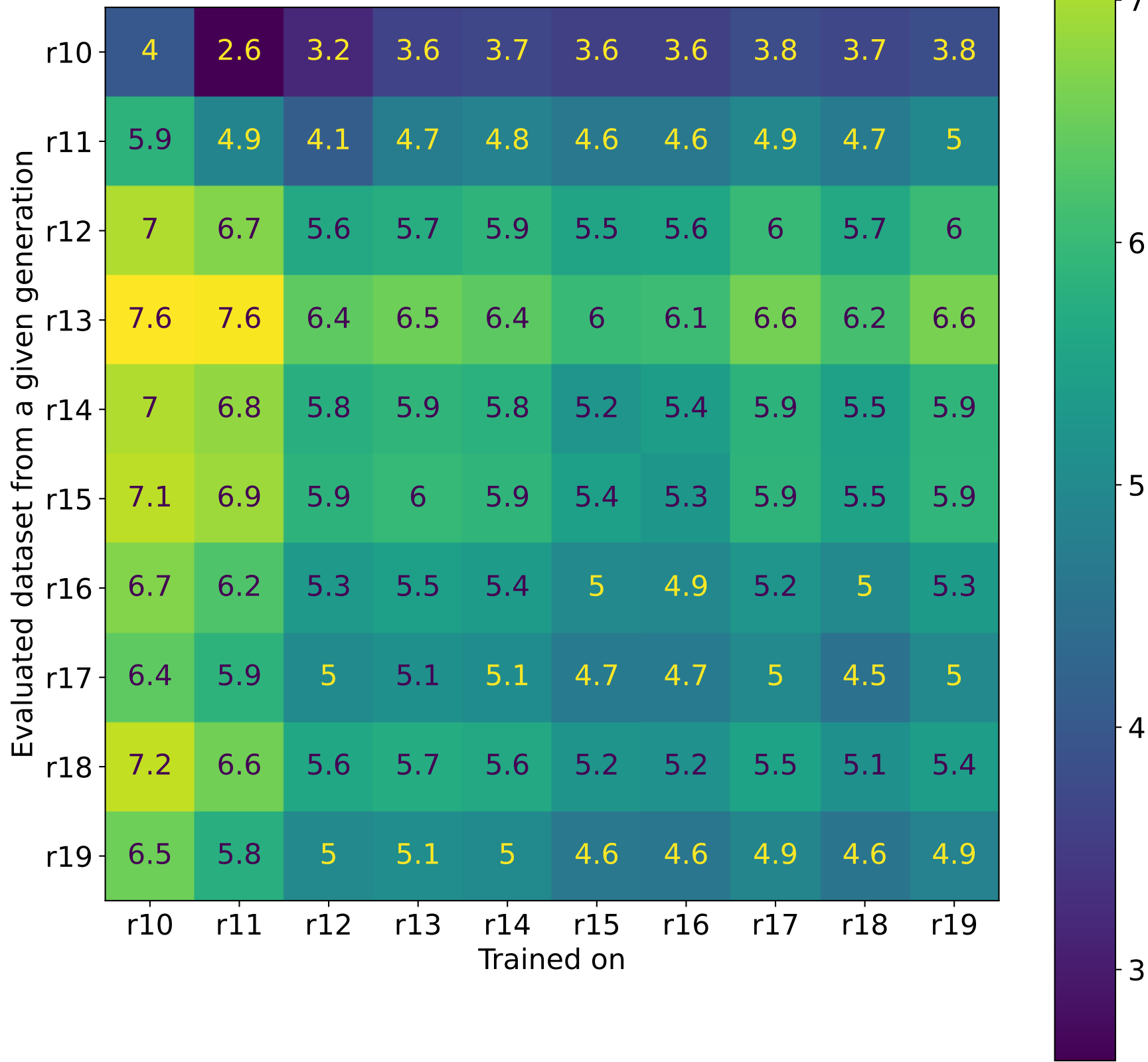

Supplement: Supplementary file 2 — Supplementary Data [file 41586_2024_7566_MOESM2_ESM.zip › images/lang/0saved/cm_1.pdf]

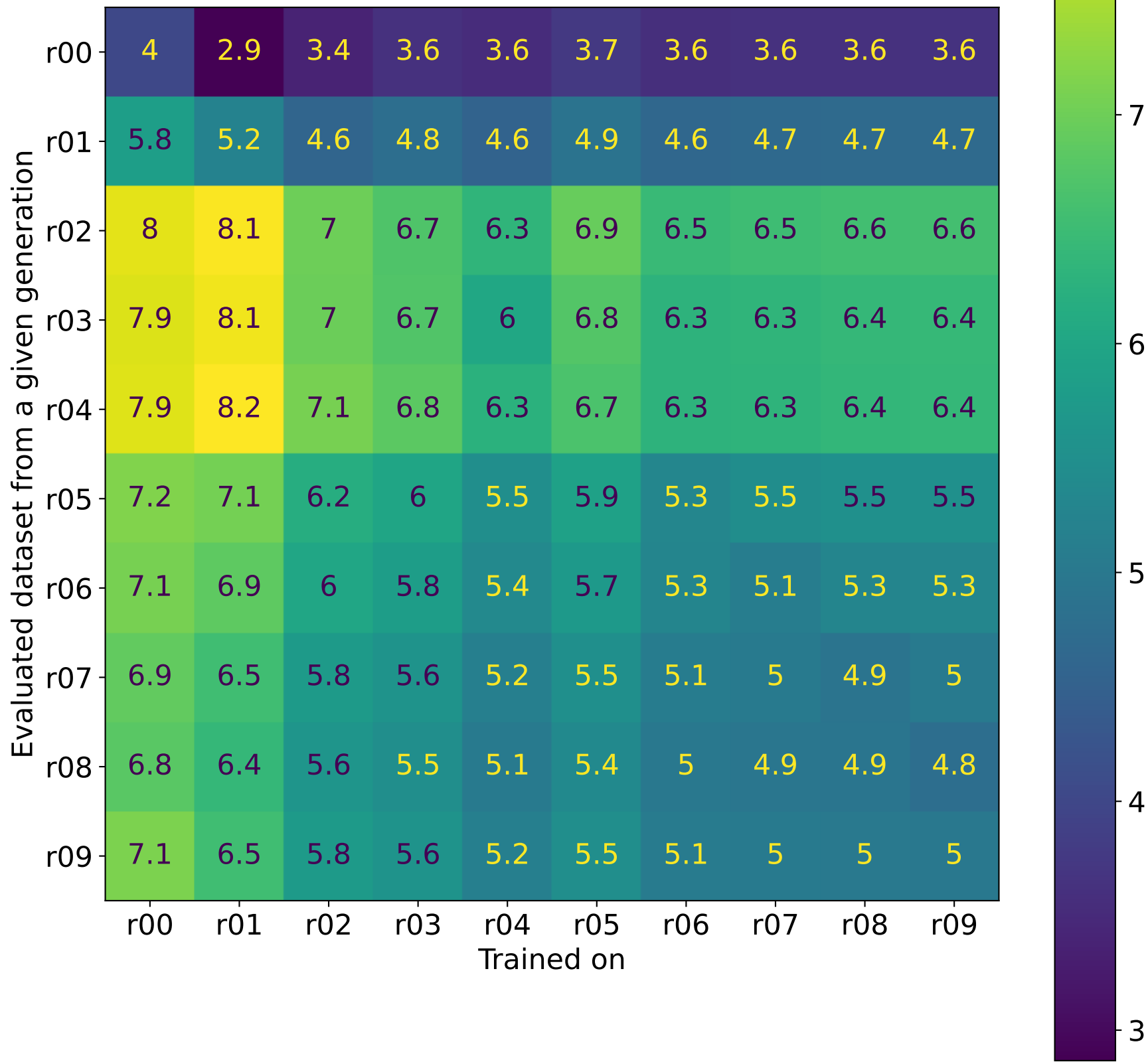

Supplement: Supplementary file 2 — Supplementary Data [file 41586_2024_7566_MOESM2_ESM.zip › images/lang/0saved/cm_0.pdf]

# Real wikitext2 test dataset

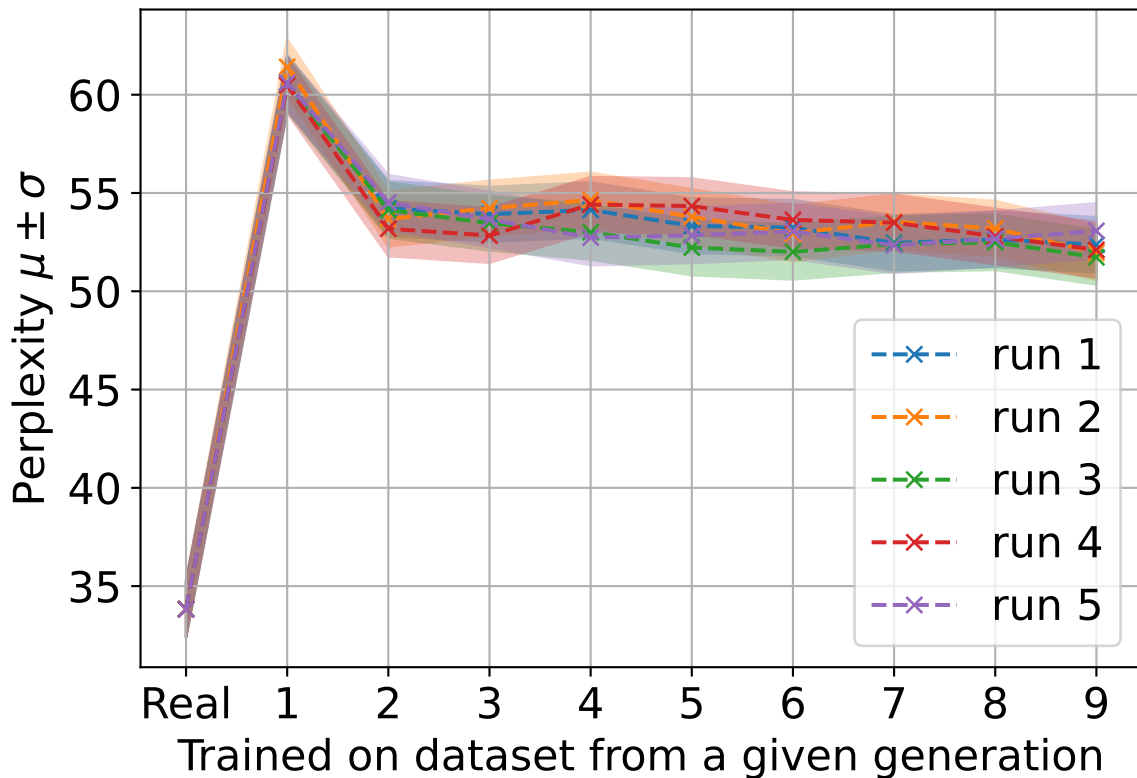

Supplement: Supplementary file 2 — Supplementary Data [file 41586_2024_7566_MOESM2_ESM.zip › images/lang/0saved/baseperf.pdf]

# OPT on Wikitext2 sampled data

## Performance of model from generation 9

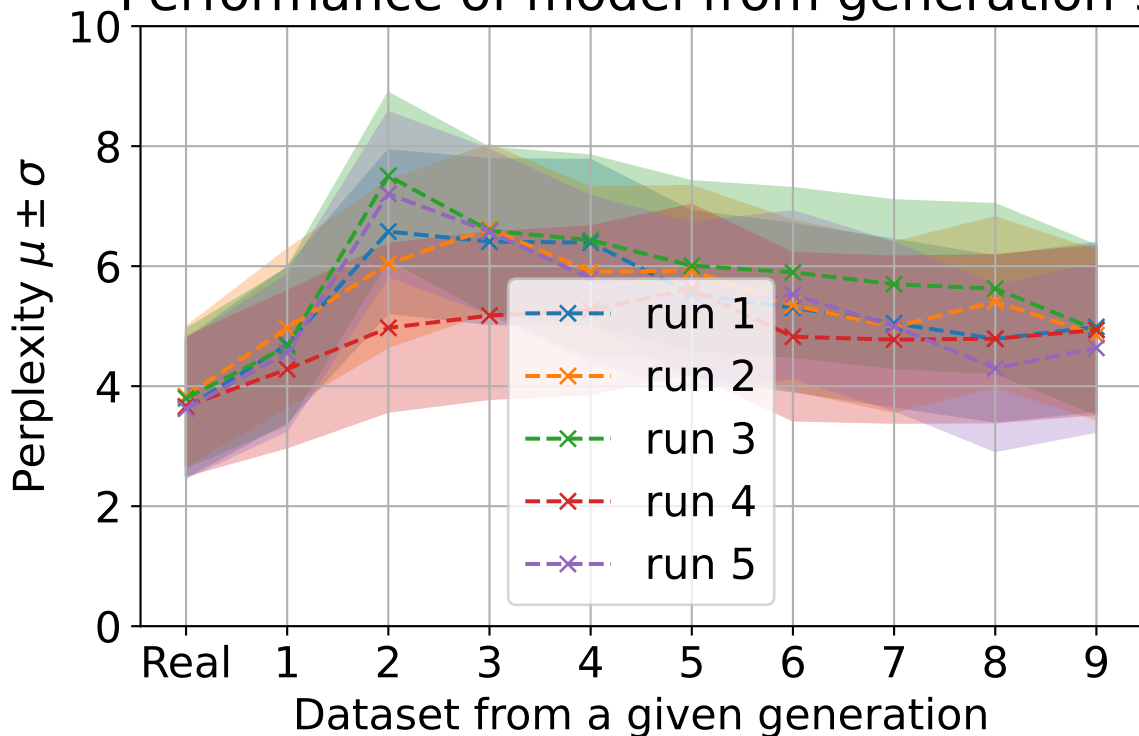

Supplement: Supplementary file 2 — Supplementary Data [file 41586_2024_7566_MOESM2_ESM.zip › images/lang/0saved/frm_9.pdf]

# OPT on Wikitext2 sampled data

## Performance of model from generation 4

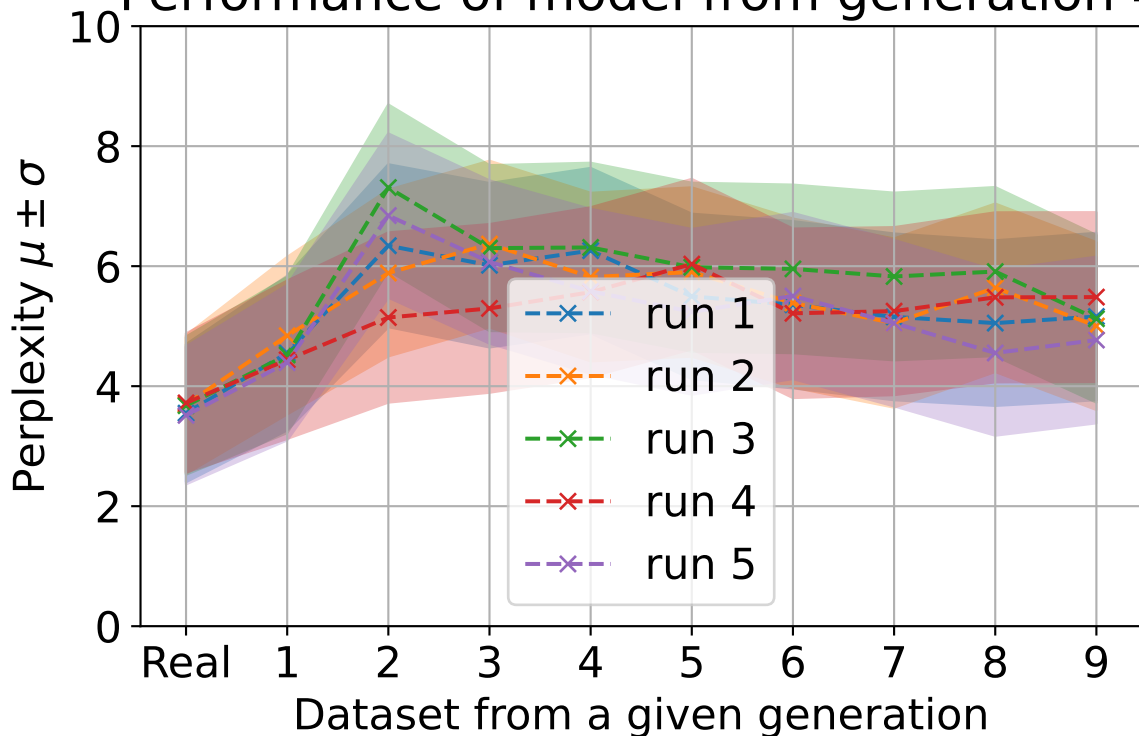

Supplement: Supplementary file 2 — Supplementary Data [file 41586_2024_7566_MOESM2_ESM.zip › images/lang/0saved/frm_4.pdf]

# OPT on Wikitext2 sampled data

## Performance of model from generation 1

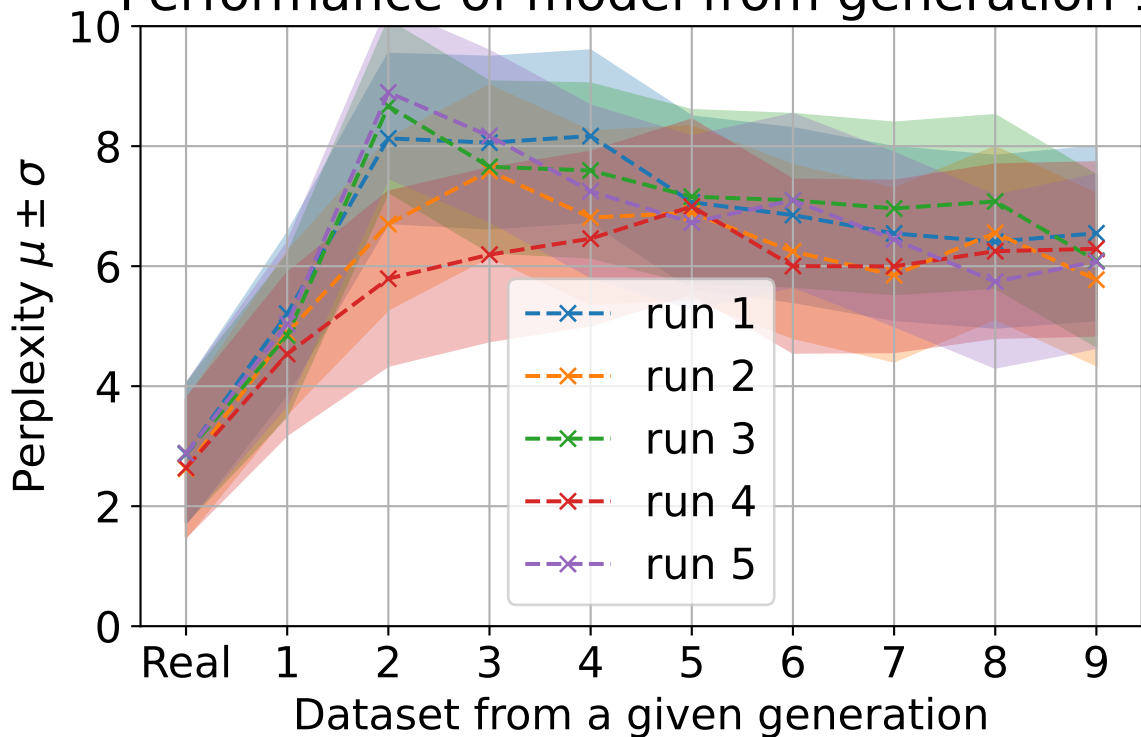

Supplement: Supplementary file 2 — Supplementary Data [file 41586_2024_7566_MOESM2_ESM.zip › images/lang/0saved/frm_1.pdf]

# OPT on Wikitext2 sampled data

## Performance of model from generation 0

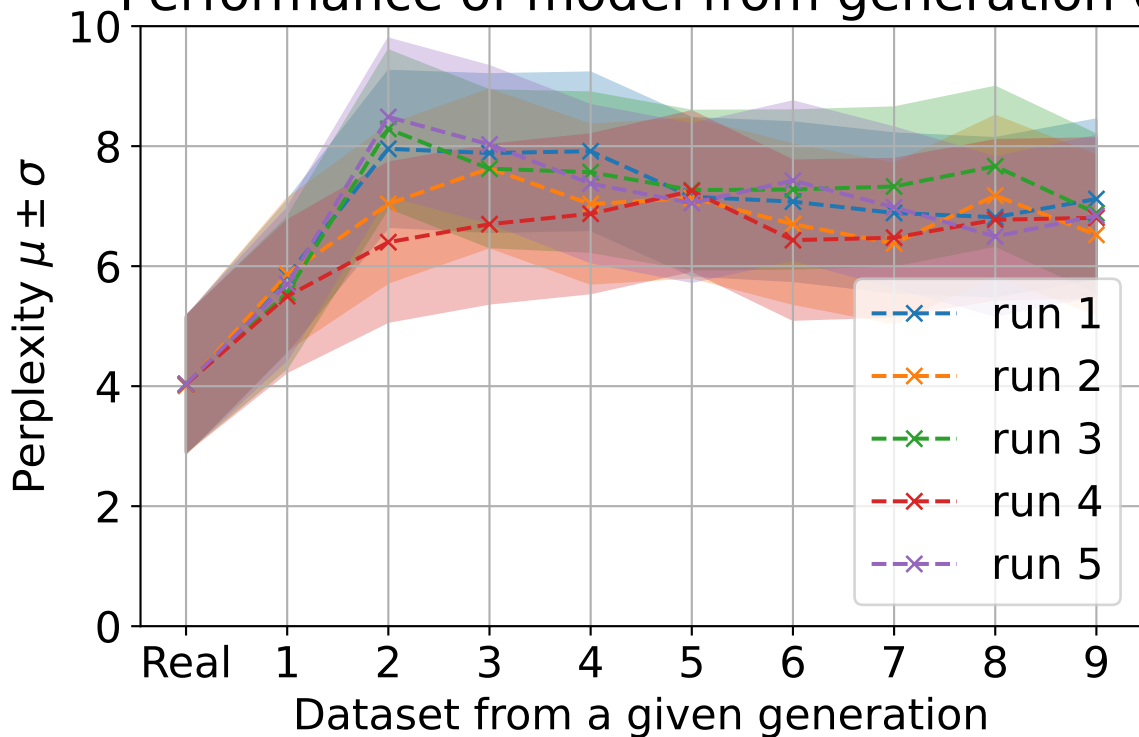

Supplement: Supplementary file 2 — Supplementary Data [file 41586_2024_7566_MOESM2_ESM.zip › images/lang/0saved/frm_0.pdf]

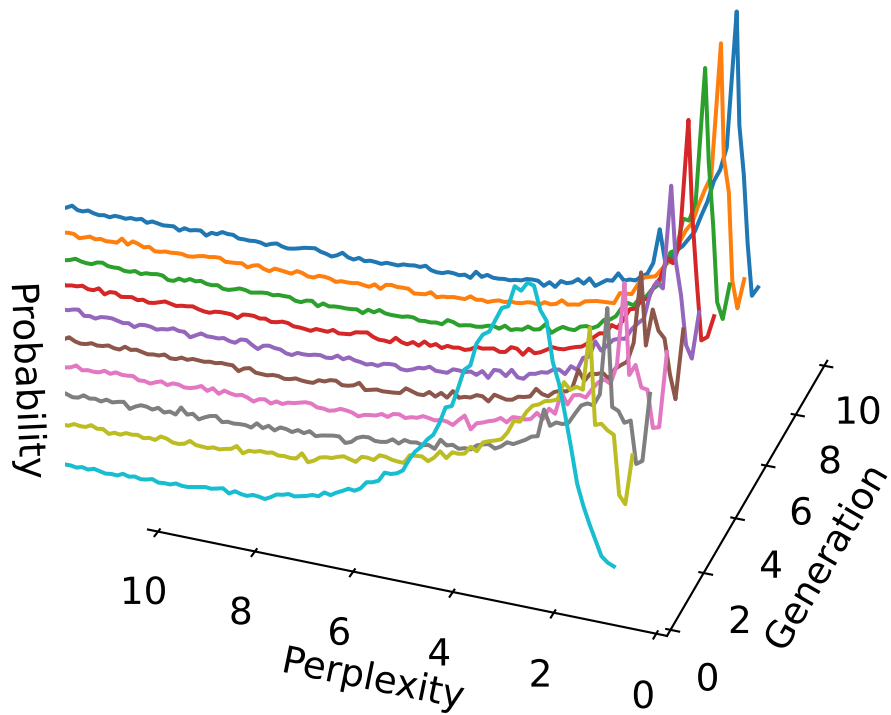

Supplement: Supplementary file 2 — Supplementary Data [file 41586_2024_7566_MOESM2_ESM.zip › images/lang/0saved/3dhist.pdf]

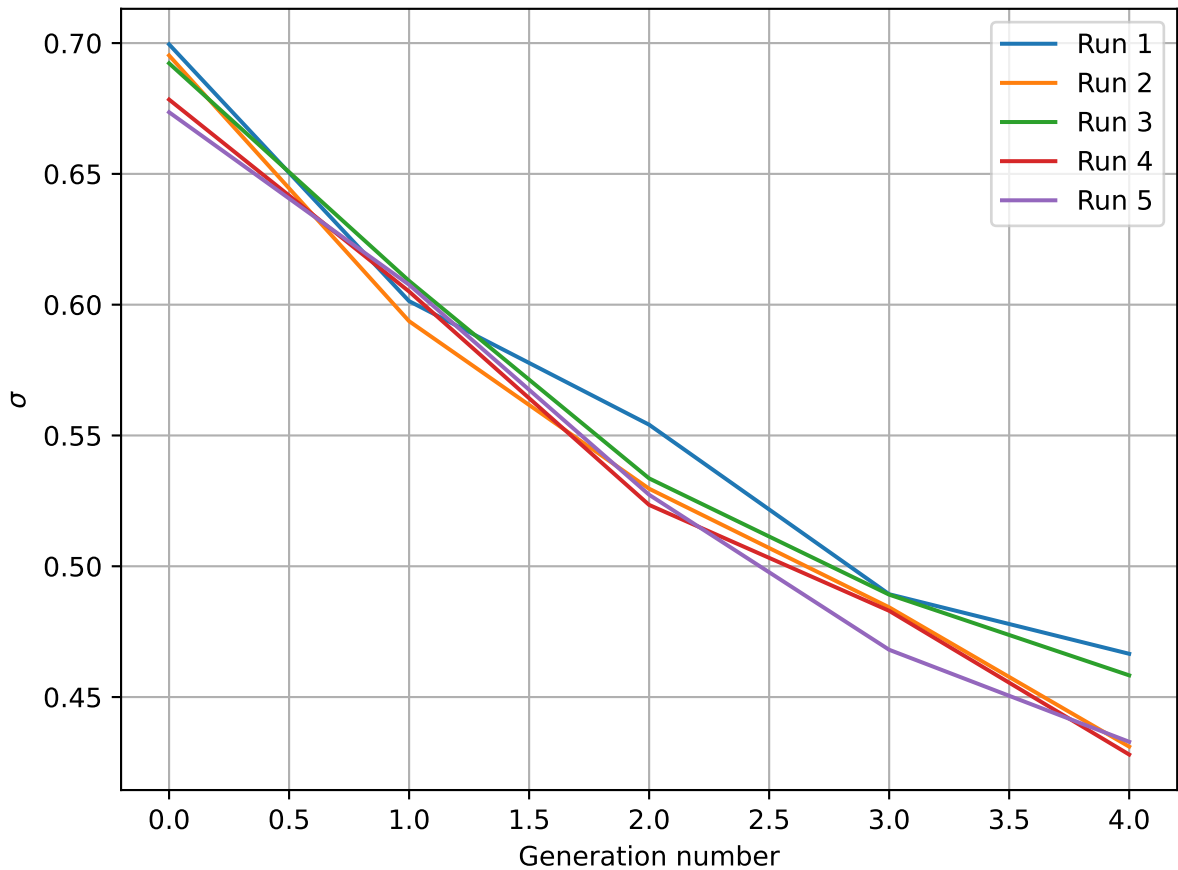

Supplement: Supplementary file 2 — Supplementary Data [file 41586_2024_7566_MOESM2_ESM.zip › images/vae/stds/vae_1.pdf]

Latents of the VAE trained on real data evaluated on generated data

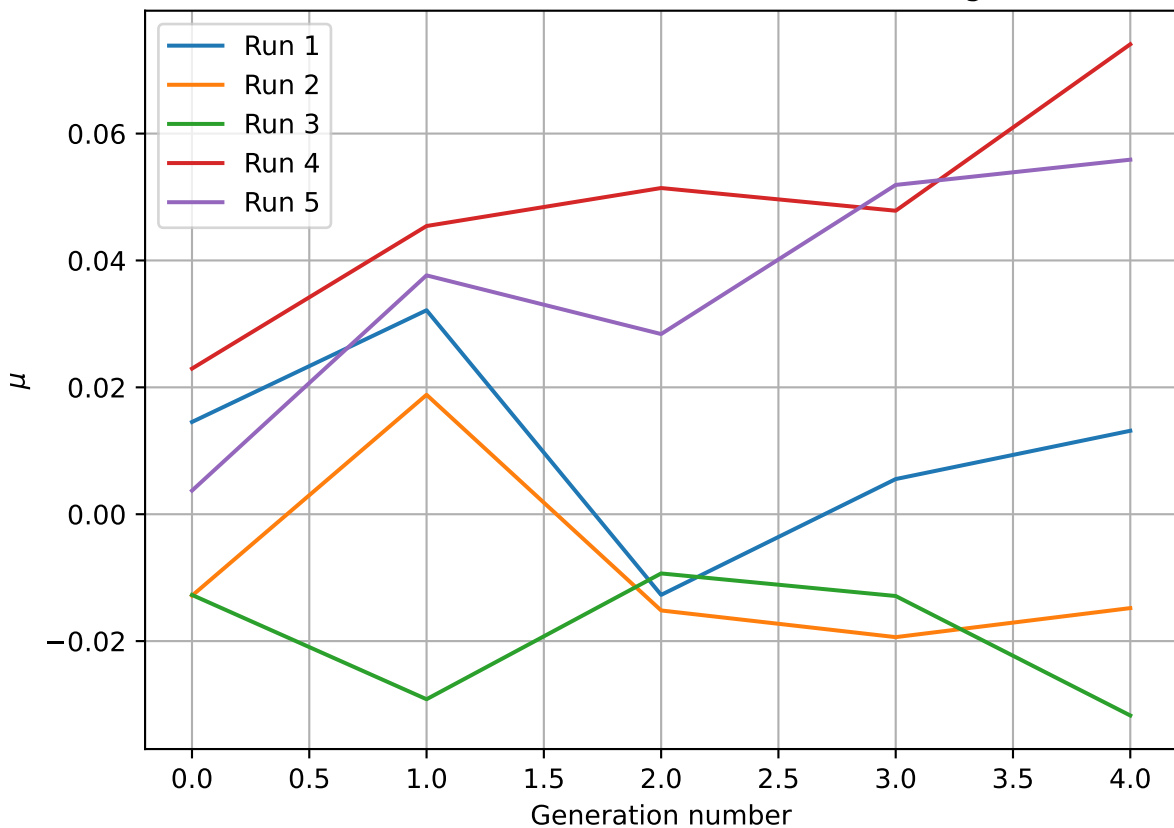

Supplement: Supplementary file 2 — Supplementary Data [file 41586_2024_7566_MOESM2_ESM.zip › images/vae/stds/vae_0.pdf]
